# Soluble amyloid-beta isoforms predict downstream Alzheimer’s disease pathology

**DOI:** 10.1186/s13578-021-00712-3

**Published:** 2021-12-11

**Authors:** Guilherme Povala, Bruna Bellaver, Marco Antônio De Bastiani, Wagner S. Brum, Pamela C. L. Ferreira, Andrei Bieger, Tharick A. Pascoal, Andrea L. Benedet, Diogo O. Souza, Ricardo M. Araujo, Bruno Zatt, Pedro Rosa-Neto, Eduardo R. Zimmer, for the Alzheimer’s Disease Neuroimaging Initiative

**Affiliations:** 1 Graduate Program in Biological Sciences: Biochemistry, Universidade Federal do Rio Grande do Sul (UFRGS), Porto Alegre, Brazil; 2 Graduate Program in Computing, Universidade Federal de Pelotas (UFPEL), Pelotas, Brazil; 3 Department of Neurology and Psychiatry, University of Pittsburgh, Pittsburgh, USA; 4 Translational Neuroimaging Laboratory, The McGill University Research Centre for Studies in Aging, 6825 LaSalle Boulevard, Verdun, QC H4H 1R3 Canada; 5 Montreal Neurological Institute, 3801 University Street, H3A 2B4 Montreal, QC Canada; 6 Department of Biochemistry, Universidade Federal do Rio Grande do Sul (UFRGS), Porto Alegre, Brazil; 7 Douglas Hospital, McGill University, 6875 La Salle Blvd-FBC room 3149, Montreal, QC H4H 1R3 Canada; 8 Department of Pharmacology, Universidade Federal do Rio Grande do Sul (UFRGS), Porto Alegre, Brazil; 9 Graduate Program in Biological Sciences: Pharmacology and Therapeutics, Universidade Federal do Rio Grande do Sul (UFRGS), Porto Alegre, Brazil

**Keywords:** Alzheimer’s disease, Amyloid-beta, Tau pathology, Neurodegeneration, Machine learning, Proteomics

## Abstract

**Background:**

Changes in soluble amyloid-beta (Aβ) levels in cerebrospinal fluid (CSF) are detectable at early preclinical stages of Alzheimer’s disease (AD). However, whether Aβ levels can predict downstream AD pathological features in cognitively unimpaired (CU) individuals remains unclear. With this in mind, we aimed at investigating whether a combination of soluble Aβ isoforms can predict tau pathology (T+) and neurodegeneration (N+) positivity.

**Methods:**

We used CSF measurements of three soluble Aβ peptides (Aβ_1﻿–38_, Aβ_1﻿–40_ and Aβ_1﻿–42_) in CU individuals (n = 318) as input features in machine learning (ML) models aiming at predicting T+ and N+. Input data was used for building 2046 tuned predictive ML models with a nested cross-validation technique. Additionally, proteomics data was employed to investigate the functional enrichment of biological processes altered in T+ and N+ individuals.

**Results:**

Our findings indicate that Aβ isoforms can predict T+ and N+ with an area under the curve (AUC) of 0.929 and 0.936, respectively. Additionally, proteomics analysis identified 17 differentially expressed proteins (DEPs) in individuals wrongly classified by our ML model. More specifically, enrichment analysis of gene ontology biological processes revealed an upregulation in myelinization and glucose metabolism-related processes in CU individuals wrongly predicted as T+. A significant enrichment of DEPs in pathways including biosynthesis of amino acids, glycolysis/gluconeogenesis, carbon metabolism, cell adhesion molecules and prion disease was also observed.

**Conclusions:**

Our results demonstrate that, by applying a refined ML analysis, a combination of Aβ isoforms can predict T+ and N+ with a high AUC. CSF proteomics analysis highlighted a promising group of proteins that can be further explored for improving T+ and N+ prediction.

**Supplementary Information:**

The online version contains supplementary material available at 10.1186/s13578-021-00712-3.

## Background

Alzheimer’s disease (AD) is the most prevalent neurodegenerative disease worldwide [1]. Its main neuropathological features involve the deposition of two proteins, amyloid-β (Aβ) and tau, into insoluble aggregates in the brain [2, 3]. Indeed, the most accepted AD theoretical model suggests that Aβ dysmetabolism triggers a cascade of downstream pathological events, including tau pathology, synaptic dysfunction, and neurodegeneration, which leads to cognitive decline and, ultimately, to dementia [4, 5].

This theoretical model relies on data derived from cross-sectional and longitudinal multicentric studies using multiple biomarkers. Currently, AD biomarkers are divided into two main classes: biofluid-based [blood and cerebrospinal fluid (CSF)] and neuroimaging [magnetic resonance imaging (MRI) and positron emission tomography (PET)] [6]. These biomarkers constitute the basis of the National Institute on Aging-Alzheimer’s Association (NIA-AA) Research Framework proposed for clinical studies, which adopted the A/T/(N) system for amyloid, tau, and neurodegeneration biomarkers [7]. In each category, biomarkers are dichotomized to indicate a normal or abnormal status [7].

Importantly, this system relies on the amyloid cascade hypothesis, i.e., the linear chain Aβ positivity (A+) → tau positivity (T+) → neurodegeneration positivity (N+) → cognitive symptoms [4, 5]. However, around 30% of cognitively unimpaired (CU) individuals are A+ but do not present any other AD pathological features [8–10]. Thus, A+, usually indexed by CSF Aβ_1﻿–42_ or PET, does not infer *per se* if an individual presents or will develop tau pathology or neurodegeneration. Therefore, it is clear that other biological processes are also critical in the progression toward clinical symptoms.

In this study, we asked (i) whether a combination of Aβ isoforms, measured in the CSF, would be capable of predicting downstream pathological biomarkers and (ii) what biological processes are related to an increase in Aβ isoforms’ prediction power over downstream AD pathology. To answer these inquiries, we aimed at predicting T+ and N+ using a combination of demographics and Aβ isoforms levels in the CSF (Aβ_1﻿–38_, Aβ_1﻿–40_, and Aβ_1﻿–42_) as input features in machine learning models (ML). We also evaluated whether CSF proteomic analyses could reveal altered biological processes heterogeneity in individuals wrongly classified in ML models.

## Methods

### ADNI description

Data used in this article are available at the Alzheimer’s Disease Neuroimaging Initiative (ADNI) database (adni.loni.usc.edu). ADNI is a longitudinal multicentric study launched in 2004, as a result of a public-private partnership, including the Foundation for the National Institutes of Health and the National Institute on Aging alongside contributors from many other sources. The study is currently in its 4th phase (ADNI1, ADNI GO, ADNI2, and ADNI3) and has recruited over 2300 participants in North America, to develop clinical, imaging, genetic, and biochemical biomarkers for the early detection and tracking of AD. More information on the study design can be found in adni.loni.esc.edu/about/.

### Eligibility criteria

In this study, data from 318 CU subjects were collected from ADNI1 and ADNI2 database. Specific criteria for inclusion in this study were the availability of CSF levels of Aβ_1﻿–38_, Aβ_1﻿–40_, and Aβ_1﻿–42_ proteins measured by 2D-ultra-performance liquid chromatography-tandem mass spectrometry (2D-UPLC-MS/MS). ADNI’s inclusion and diagnostic criteria have been described elsewhere [11].

### CSF biomarker collection and analysis

CSF Aβ_1–38_, Aβ_1–40_, and Aβ_1–42_ peptide levels were measured using the 2D-UPLC-MS/MS method (Waters^®^ XEVO-TQ-S), which had been previously described [12] and has been recently revalidated. This updated technique has been recognized as an accepted analytical reference by the Joint Committee for Traceability in Laboratory Medicine (JCTLM), in whose database it was published under the JCTLM Identification Number C12RMP1. For defining T+ and N+, p-tau (Thr-181) and t-tau levels used in this study were measured by the Elecsys^®^ immunoassay, with T+ defined as CSF p-tau (181-Thr) > 19.2 pg/mL and N+ defined as CSF t-tau > 242 pg/mL [13]. Data for the 2D-UPLC-MS/MS and Elecsys^®^ methods are available, respectively, at the ADNI database under the file names “UPENNMSMSABETA.csv” and “UPENNBIOMK9_04_19_17.csv”.

### Statistical analysis

All statistical analyses were performed in GraphPad Prism 8. Data are expressed as mean ± standard deviation (SD). Normality was evaluated using histograms and quantile plots. Because samples did not have Gaussian distributions, comparisons between groups were carried out using MannWhitney test. P-values of less than 0.05 were reported as statistically significant.

### Machine learning framework

We developed a ML framework that combines multiple techniques and models to predict T+ and N+ with the use of CSF Aβ isoform levels, demographic information and APOE ɛ4 status. The framework was coded in Python (version 3.6.8, https://www.python.org/), using the scikit-learn (version 0.20.2, https://scikit-learn.org/) and xgboost (version 0.81, https://xgboost.readthedocs.io/) libraries. The supervised ML algorithms used in our framework are composed of Logistic Regression, Naive Bayes, k-Nearest Neighbors (kNN), Support Vector Classifier (SVC), Decision Trees, Random Forest, Gradient Boosting, XGBoost, and AdaBoost.

As input features for our framework, we used Aβ peptide levels (Aβ_1﻿–38_, Aβ_1﻿–40_, and Aβ_1﻿–42_), demographic information (age, sex and years of education), and APOE ɛ4 status. For feature selection, we evaluated all possible feature combinations, generating 1023 subsets. For each feature subset, we performed the nested cross-validation (CV) technique. Here, we used the nested CV since we needed to train different ML models together with its hyperparameter optimization. The nested CV has an inner CV loop nested in outer CV. The inner loop is composed of a 2-fold CV, and it is responsible for model selection and hyperparameter tuning, which is similar to a validation set. The outer loop, however, is composed of a 5-fold CV and it is used for error estimation, as a test set. The nested cross-validation uses the area under de curve (AUC) metric to select the best hyperparameters and models. Then, an independent test set is used to test the overall performance of the best model and to generate the AUC result. The hyperparameters evaluated for each ML algorithm used in this work are shown in Table 1. After obtaining the AUC results for tuned ML algorithms with the nested cross-validation, only the model that presented the best performance is chosen for each feature subset. Among all these models, we selected the best one and then extracted the AUC for the independent test set.

**Table 1:** Hyperparameters evaluated for the machine learning models

Algorithm	Fixed parameters	Iterated parameters
Logistic Regression	solver: lbfgs max_iter: 250 penalty: l2	C: [0.0001, 0.001, 0.01, 0.1, 1, 10, 100, 1000]
Naive Bayes	–	-
kNN	algorithm: ball_tree leaf_size: 50	n_neighbors: [1,2,3,4,5,6,7,8,9] p: [1,2]
SVC	–	for kernels: [rbf, poly, sigmoid] C: [−4, −3, −2, −1, 0, 1, 2, 3] for kernel: linear gamma: [0.00001, 0.0001, 0.001, 0.01, 0.1] C: [0.0001, 0.001, 0.01, 0.1, 1, 10, 100, 1000]
Decision Trees	–	max_depth: [1,2,3,4,5,6,7,8,9] criterion: [gini, entropy]
Random Forest	–	max_depth: [3,4,5,8,10] n_estimators: [5, 20, 50, 100, 200, 500, 1000]
Gradient Boosting	–	max_depth: [3,4,5,8,10] leargning_rate: [0.01, 0.05, 0.1, 0.2] n_estimators: [5, 20, 50, 100, 200, 500, 1000]
XGBoost	–	max_depth: [6,7,8] leargning_rate: [0.01, 0.025, 0.05, 0.075, 0.1] n_estimators: [5, 20, 50, 100, 200, 500, 1000]
AdaBoost	–	learning_rate: [0.25, 0.5, 1.0, 1.25, 1.5] n_estimators: [20, 50, 100, 150, 200]

kNN: k-Nearest Neighbors; SVC: Support Vector Classifier

### CSF proteomics analysis

Processed CSF proteomics data were collected from the ADNI database. Samples were measured using the LC/MS-MRM method [12]. Proteins and peptides were selected based upon their previous detection in CSF, relevance to AD, and previous results from the Rules Based Medicine (RBM) multiplex immunoassay analysis of ADNI CSF. The final MRM panel consisted of 567 peptides representing 221 proteins. From these 567 peptides, 320 were detectable in > 10% of ADNI samples and are available in the file “CSFMRM.csv”.

From the previously included CU individuals, only 76 presented CSF proteomics data in the ADNI database and were included in further analyses. CSF proteomics analysis was performed comparing T− (n = 55) and T+ (n = 21) individuals and N− (n = 57) and N+ (n = 19). All proteomic analyses were implemented in an R statistical environment. Differentially expressed analysis was computed for T−/T+ and N−/N+ groups independently, using the LIMMA (version 3.46.0) package [14], and considering FDR-adjusted p-value < 0.05 as differentially expressed proteins (DEP) criteria. Finally, functional enrichment analyses of gene ontology (GO) biological processes and KEGG pathways were computed and visualized using the clusterProfiler (version 3.18.1) and Goplot (version 1.0.2) packages [15, 16].

## Results

### Sample characteristics

We included 318 CU individuals from ADNI, whose CSF had been analyzed with 2D-UPLC-MS/MS. Characteristics of the ADNI cohort and the different A, T, and N status of samples are provided in Table 2. Population characteristics were compared between positive and negative group status for each of the above-mentioned biomarker categories. A+ and T+ showed significantly more APOE ɛ4 carriers than Aβ negative (A−) and tau negative (T−) groups. As already observed in previous studies, APOE ɛ4 carriers are associated with decreased Aβ_1﻿–42_ and elevated p-tau in the CSF [14, 15]. T+ and N+ presented elevated age, when compared with T− and neurodegeneration negative (N−) groups, respectively. No significant differences were observed in sex, years of education, Mini-Mental State Examination (MMSE), and Alzheimer’s Disease Assessment Scale-Cognitive Subscale (ADAS-Cog) among groups.

**Table 2:** Sample characteristics

Characteristic	CU	A−	A+	T−	T+	N−	N+
Number of individuals	318	60	50	52	58	67	43
Sex (% female)	50%	51.67%	48%	50%	50%	50.75%	48.84%
Age (y)	75.66 ± 5.22	75.37 ± 5.67	76.01 ± 4.67	73.87 ± 4.54	77.26 ± 5.32^b***^	74.46 ± 4.66	77.52 ± 5.55^c**^
Education (y)	15.73 ± 2.83	15.42 ± 2.68	16.1 ± 2.99	15.77 ± 2.77	15.69 ± 2.91	15.57 ± 3.1	15.98 ± 2.37
MMSE	29.08 ± 1.03	28.98 ± 1.1	29.2 ± 0.95	29.13 ± 0.93	29.03 ± 1.12	29.01 ± 1.05	29.19 ± 1.01
ADAS-Cog	6.42 ± 2.92	6.09 ± 2.91	6.81 ± 2.92	6.18 ± 2.85	6.64 ± 2.99	6.22 ± 2.69	6.73 ± 3.27
APOE ε4 carriers (%)	24.55%	11.67%	40%^a***^	15.38%	32.76% ^b*^	19.40%	32.56%

CU: Cognitively Unimpaired; A+: Amyloid-beta positive; A−: Amyloid-beta negative; T+: Tau positive; T−: Tau negative; N+: Neurodegeneration positive; N−: Neurodegeneration negative; y: year; MMSE: Mini-Mental State Examination; ADAS-Cog: Alzheimer’s Disease Assessment Scale-Cognitive subscale. Statistical differences for numerical characteristics were tested using t test. Statistical differences for sex and APOE status were tested using Fisher’s exact test. (^*^p < 0.05, ^**^p ≤ 0.01, ^***^p ≤ 0.001)

^a^ significantly different from A−, ^b^ significantly different from T−, ^c^ significantly different from N−﻿

### Changes in Aβ soluble isoforms in T+ and N+ CU individuals

**Fig. 1:**
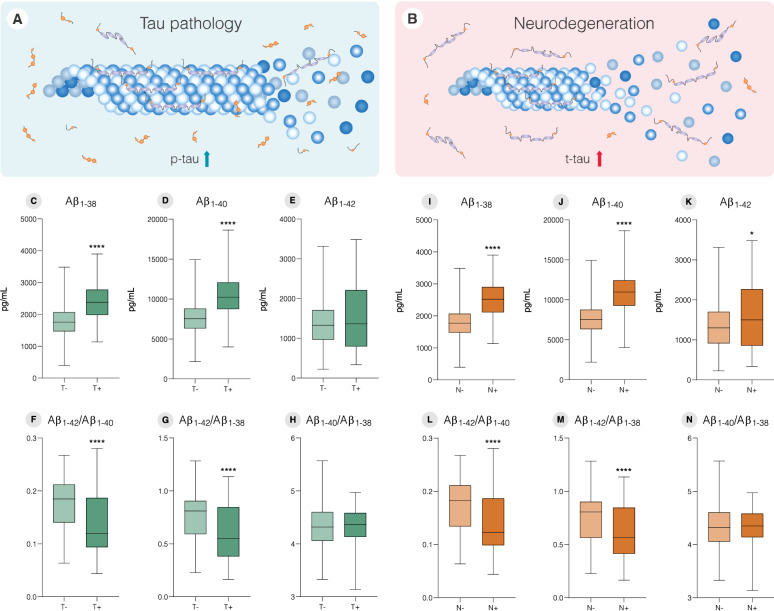
Aβ isoforms levels discriminate tau pathology positivity (T+) and neurodegeneration positivity (N+) in CU individuals. **A** T+ defined as CSF p-tau > 19.2 pg/mL. **B** N+ defined as CSF t-tau > 242 pg/mL. **C** Aβ_1−38_, **D** Aβ_1−40_ and **E** Aβ_1−42_ levels for T− and T+ individuals. **F** Aβ_1−42_/Aβ_1−40_, **G** Aβ_1−42_/Aβ_1−38_ and **H** Aβ_1−40_/Aβ_1−38_ ratios for T− and T+ individuals. **I** Aβ_1−38_, **J** Aβ_1−40_ and **K** Aβ_1−42_ levels for N− and N+ individuals. **L** Aβ_1−42_/Aβ_1−40_, **M** Aβ_1−42_/Aβ_1−38_ and **N** Aβ_1−40_/Aβ_1−38_ ratios for N− and N+ individuals. Boxplots are displayed as median (center line) and extend from the 25th to 75th percentiles. The whiskers go down to the smallest value and up to the largest. Statistical differences were tested using Mann-Whitney test (*p ≤ 0.05, ***p ≤ 0.001, ****p ≤ 0.0001)

Figure 1 compares Aβ isoform levels and their respective ratios between T+ and T− (Fig. 1a), and N+ and N− (Fig. 1b). When comparing T status, T+ group presented higher levels of Aβ_1﻿–38_ (Fig. 1c, T− = 1764 ± 496.1 pg/mL, T+ = 2411 ± 566.95 pg/mL, p < 0.0001) and Aβ_1﻿–40_ (Fig. 1d, T− = 7617 ± 2052 pg/mL, T+ = 10,424 ± 2529 pg/mL, p < 0.0001). Additionally, a decrease in Aβ_1﻿–42_**/**Aβ_1﻿–40_ (Fig. 1f, T− = 0.1749 ± 0.05, T+ = 0.1381 ± 0.06, p < 0.0001) and Aβ_1﻿–42_**/**Aβ_1﻿–38_ ratios (Fig. 1 g, T− = 0.7610 ± 0.22, T+ = 0.6014 ± 0.25, p < 0.0001) was observed in T+ individuals. However, we did not observe any significant difference in Aβ_1﻿–42_ levels (Fig. 1e, T− = 1353 ± 559.4 pg/mL, T+ = 1492 ± 784 pg/mL, p = 0.41) and Aβ_1﻿–40_**/**Aβ_1﻿–38_ ratio (Fig. 1 h, T− = 4.354 ± 0.42, T+ = 4.329 ± 0.35, p = 0.60) between T+ and T− groups.

For N+ individuals, Aβ_1﻿–38_ (Fig. 1i, N− = 1760 ± 469.6 pg/mL, N+ = 2503 ± 567.2 pg/mL, p < 0.0001), Aβ_1﻿–40_ (Fig. 1j, N**−** = 7593 ± 1945 pg/mL, N+ = 10,838 ± 2503 pg/mL, p < 0.0001), and Aβ_1–42_ (Fig. 1k, N− = 1328 ± 565.1 pg/mL, N+ = 1575 ± 778.8 pg/mL, p = 0.03) measures were significantly elevated when compared to N−, along with a decrease in Aβ_1﻿–42_**/**Aβ_1﻿–40_ ratio (Fig. 1l, N− = 0.1720 ± 0.05, N+ = 0.1411 ± 0.05, p < 0.0001) and Aβ_1﻿–42_**/**Aβ_1﻿–38_ ratio (Fig. 1m, N− = 0.7483 ± 0.23, N+ = 0.6146 ± 0.25, p < 0.0001). By contrast, Aβ_1﻿–40_**/**Aβ_1﻿–38_ ratio (Fig. 1n, N− = 4.350 ± 0.41, N+ = 4.336 ± 0.35, p = 0.78) does not differ between N+ and N− groups.

To test whether single Aβ isoforms or its ratios can predict downstream AD pathological processes in CU individuals, we used logistic regression models. The AUC results for predicting T+ and N+ individuals are shown in Table 3. Among all results, Aβ_1–38_ and Aβ_1–40_ seem to be the most reliable features to predict T+, with an AUC of 0.811 for both Aβ isoforms. For predicting N+, Aβ_1﻿–38_ and Aβ_1﻿–40_ showed similar results, with AUCs of 0.847 and 0.855, respectively. On the other hand, Aβ_1﻿–42_ presented an AUC of 0.580 for predicting N+ and 0.529 for T+.

**Table 3:** AUC results for predicting T+ and N+ in CU individuals using single Aβ isoforms or its ratios

Prediction	Aβ_1−38_	Aβ_1−40_	Aβ_1−42_	Aβ_1−42_/Aβ_1−40_	Aβ_1−42_/Aβ_1−38_	Aβ_1−40_/Aβ_1−38_
T+	0.811	0.811	0.529	0.693	0.682	0.484
N+	0.847	0.855	0.580	0.663	0.652	0.479

T+: Tau positive; N+: Neurodegeneration positive; Aβ: Amyloid-beta

### Machine learning framework

**Fig. 2:**
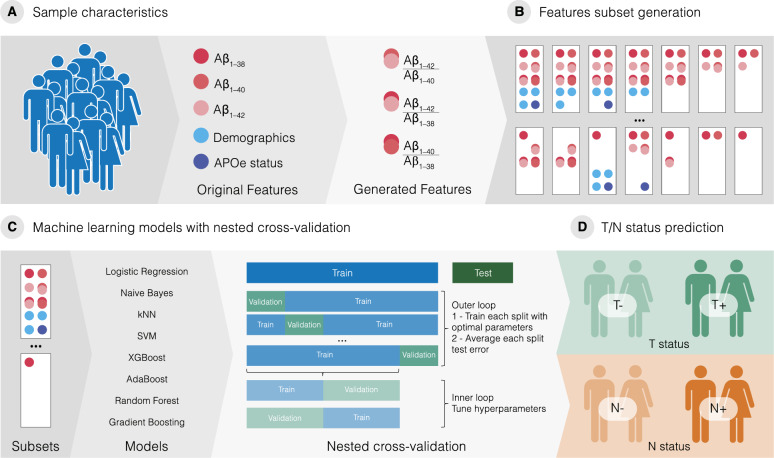
Machine learning framework for predicting tau pathology and neurodegeneration. **A** Cognitively unimpaired (CU) individual’s cerebrospinal fluid (CSF) levels of Aβ_1–38_, Aβ_1–40_ and Aβ_1–42_, demographics data and APOE ε4 status were used for feature generation. **B** All possible combinations of features were generated using the feature set. **C** The subsets were used for generating tuned machine learning models validated with nested cross-validation aiming to (**D**) identify tau pathology (T+) and neurodegeneration (N+) positivity

Aiming at better predictive models, we proposed a ML framework, which is presented in Fig. 2. Aβ isoforms in the CSF (Aβ_1﻿–38_, Aβ_1﻿–40_, and Aβ_1﻿–42_; measured by 2DUPLCMS/MS), APOE ɛ4 carrier status, and demographic information (age, sex, and years of education) were used as input features. Besides, for feature generation, Aβ isoforms were used either alone or combined in ratios (Fig. 2a). In the feature subset generation step (Fig. 2b), all possible combinations of features were created (1023 different subsets). Then, for each subset, two models were selected using the nested CV technique (Fig. 2c): one for T+ prediction and another to predict N+ (Fig. 2d).

In our ML framework, to choose the best model for each subset to classify T+ and N+, we evaluated the use of the following ML algorithms: Logistic Regression, Naïve Bayes, kNN, SVC, Decision Trees, Random Forest, Gradient Boosting, XGBoost, and AdaBoost within the nested CV technique. For each subset, the best model was defined based on the model’s AUC obtained from the validation set. The top 1 model among the 1023 models (one for each subset) was evaluated using an independent test set and was defined as the best model to predict T+ or N+.

### Tau pathology positivity prediction

**Fig. 3:**
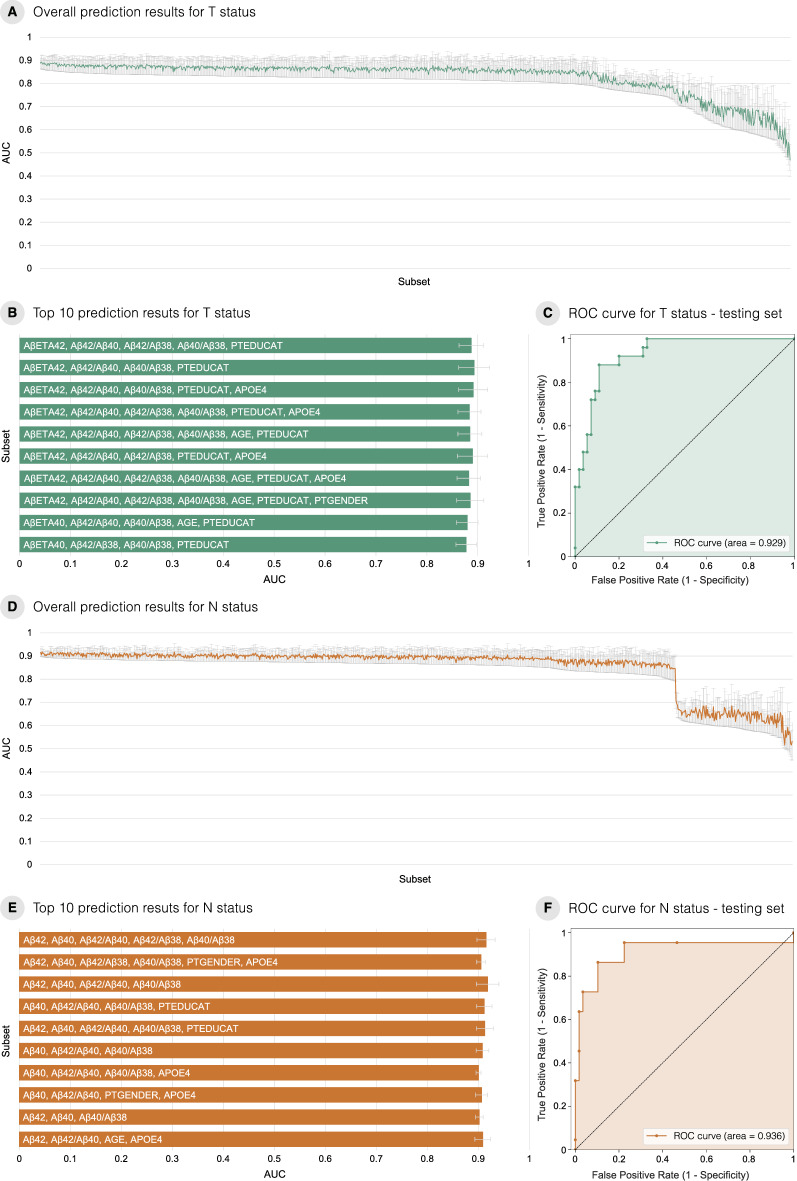
Results for predicting tau pathology (T) and neurodegeneration (N) status. **A** Area under the ROC curve (AUC) results (vertical axis) for all 1023 subsets to predict T status ordered by AUC – standard deviation (SD). **B** AUC results (horizontal axis) for the top 10 models (vertical axis) to predict T status. **C** ROC curve for the best model to predict T status using the independent test set. **D** AUC results (vertical axis) for all 1023 subsets to predict N status ordered by AUC – SD. **E** AUC results (horizontal axis) for the top 10 models (vertical axis) to predict N status. **F** ROC curve for the best model to predict N status using the independent test set

From our proposed ML framework, 1023 tuned ML models were generated for predicting T+ (Additional file 1). Figure 3a shows the AUC results for predicting T+ horizontally ordered by AUC – SD. In Fig. 3b, the best 10 models are ranked. Among the 10 models, all of them presented similar results, ranging from 0.877 to 0.887.

The top 1 model was a logistic regression model using Aβ_1﻿–42_, Aβ_1﻿–42_/Aβ_1﻿–40_, Aβ_1﻿–42_/Aβ﻿_1﻿–38_, Aβ_1﻿–40_/Aβ_1﻿–38_, and years of education as input features. The AUC result obtained for the validation set was 0.881 ± 0.024. For the independent test set, we achieved an AUC of 0.929 (Fig. 3c).

### Neurodegeneration positivity prediction

For N+ prediction, we generated another 1023 models using the same method (Additional file 2). The AUC results for the N+ predictions are shown in Fig. 3d horizontally ordered by AUC – SD. The best 10 models were ranked and plotted on the graph represented in Fig. 3e. The best 10 models presented similar results, ranging from 0.909 to 0.915.

A kNN generated the best results, which had Aβ_1–42_, Aβ_1﻿–40_, Aβ_1﻿–42_/Aβ_1﻿–40_, Aβ_1﻿–42_/Aβ_1﻿–38_, and Aβ_1﻿–40_/Aβ_1﻿–38_ as input features. The AUC result for the validation set for this model was 0.915 ± 0.018. The independent test set achieved an AUC of 0.936 (Fig. 3f).

### CSF proteomics of T+ and N+ CU individuals

**Fig. 4:**
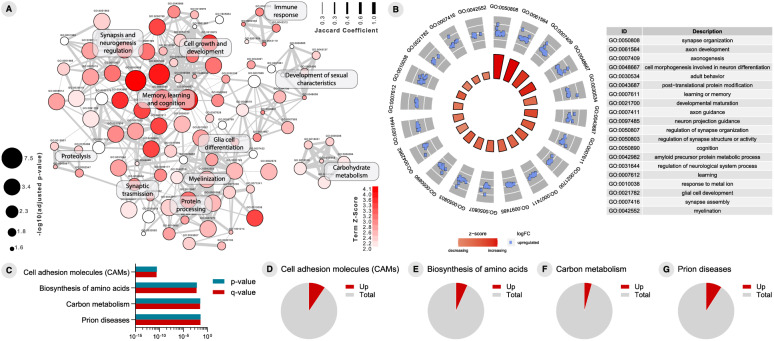
Proteome analyses results of cerebrospinal fluid (CSF) cells between T− and T+ cognitively unimpaired (CU) individuals. **A** Gene ontology (GO) network of enriched terms were constructed from differentially expressed proteins mapping the node sizes to GO term significance and edge width to shared protein proportions (Jaccard coefficient). **B** Radial plot of top 20 enriched GO terms. **C** Enriched pathways obtained from functional enrichment of KEGG terms. **D–G** Pie charts of enriched KEGG pathways showing the proportion of proteins upregulated in T+ vs. T− comparison

**Fig. 5:**
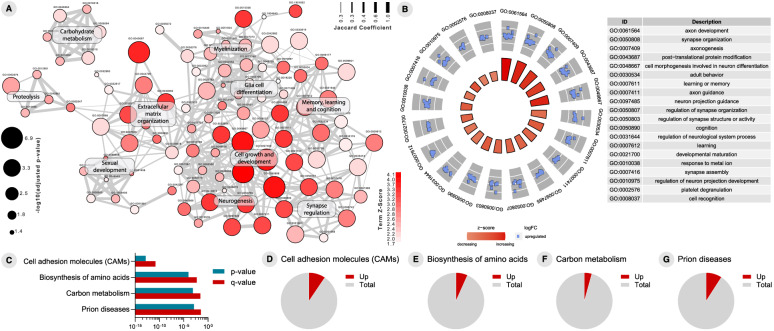
Proteome analyses results of cerebrospinal fluid (CSF) cells between N− and N+ cognitively unimpaired (CU) individuals. **A** Gene ontology (GO) network of enriched terms were constructed from differentially expressed proteins mapping the node sizes to GO term significance and edge width to shared protein proportions (Jaccard coefficient). **B** Radial plot of top 20 enriched GO terms. (**C**) Enriched pathways obtained from functional enrichment of KEGG terms. **D–G** Pie charts of enriched KEGG pathways showing the proportion of proteins upregulated in N+ vs. N− comparison

To address T+ and N+ CU individuals’ functional changes in biological processes, we performed CSF-based proteomics analyses. A total of 112 DEPs were observed in the CSF of CU T+ compared to T− subjects (Additional file 3). The enrichment analysis of GO biological processes in T+ individuals evidenced processes related to myelinization, synapse and neurogenesis regulation, immune response, carbohydrate metabolism, memory and learning, and glial cell differentiation (Fig. 4a). Figure 4b depicts top 20 GO terms enriched in T+ subjects compared to T−. To identify the most affected pathways related to changes in proteomics profile of T+, we performed an enrichment analysis using canonical pathways described in the KEGG pathway database [17]. This revealed a significant enrichment of 112 DEPs in 4 signaling pathways: “cell adhesion molecules”, “biosynthesis of amino acids”, “carbon metabolism”, and “prion disease” (Fig. 4c–g). Regarding proteomics analysis of N+, we identified 123 DEPs when compared to N− individuals (Additional file 4). Of note, T+ and N+ subjects share 101 DEPs. Functional enrichment analyses revealed an overlap of enriched GO terms in N+ individuals and T+ individuals (Fig. 5a). Synapse organization, learning and memory processes, and APP metabolic processes are among the top 20 GO terms enriched in N+ (Fig. 5b). Interestingly, the same 4 KEGG pathways enriched for T+ were found enriched for N+ individuals (Fig. 5c–g).

### CSF proteomics analysis for ML wrong predictions

Because Aβ isoforms predicted T+ and N+ outcomes with an AUC of up to 0.936, we next aimed, with a second proteomics analysis, at identifying differences in biological processes occurring in CU individuals that were wrongly classified by our ML model in the test set. First, we stratified the ML predictions for T+ in false-positive (n = 17), false-negative (n = 23), true-positive (n = 51), and true-negative (n = 147). Proteomic analyses for N+ prediction model was not carried out, since few wrong predictions were generated, leading to a small sample size.

**Fig. 6:**
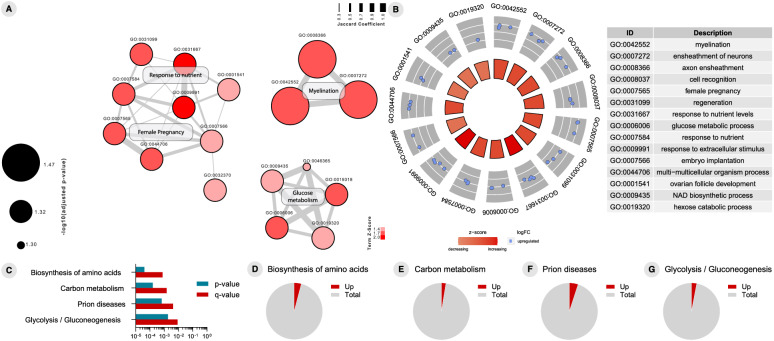
Proteome analyses results of cerebrospinal fluid (CSF) cells between true positive (TP) and false positive (FP) predictions for tau pathology positivity (T+) in cognitively unimpaired (CU) individuals. **A** Gene ontology (GO) network of enriched terms were constructed from differentially expressed proteins mapping the node sizes to GO term significance and edge width to shared protein proportions (Jaccard coefficient). **B** Radial plot of top 15 enriched GO terms. **C** Enriched pathways obtained from functional enrichment of KEGG terms. **D–G** Pie charts of enriched KEGG pathways showing the proportion of proteins upregulated in TP vs. FP comparison

**Fig. 7:**
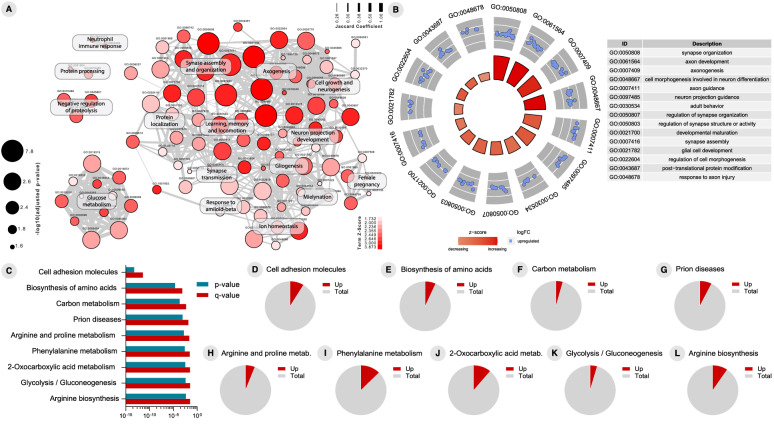
Proteome analyses results of cerebrospinal fluid (CSF) cells between true negative (TN) and false negative (FN) predictions for tau pathology positivity (T+) in cognitively unimpaired (CU) individuals. **A** Gene ontology (GO) network of enriched terms were constructed from differentially expressed proteins mapping the node sizes to GO term significance and edge width to shared protein proportions (Jaccard coefficient). **B** Radial plot of top 15 enriched GO terms. (**C**) Enriched pathways obtained from functional enrichment of KEGG terms. **D–L** Pie charts of enriched KEGG pathways showing the proportion of proteins upregulated in TN vs. FN comparison

We identified 17 upregulated DEPs between true-positive and false-positive (Fig. 6a) and 67 upregulated DEPs between true-negative and false-negatives for T+ individuals (Fig. 7a). Interestingly, enrichment analysis of GO biological processes revealed that processes related to myelinization, and glucose metabolism are enriched when comparing false-positive and true-positive predictions for T+ (Fig. 6a, b). When considering the false-negative and true-negative predictions for T+, DEPs related to glucose metabolism, synapse transmission, gliogenesis, and axogenesis appeared among the enriched GO terms (Fig. 7a, b). Finally, to recognize the most affected pathways related to changes in proteomics profile of individuals that were wrongly predicted, we performed an enrichment analysis using canonical pathways described in the KEGG pathway database. This revealed a significant enrichment of DEPs in pathways including “biosynthesis of amino acids”, “glycolysis/gluconeogenesis”, “carbon metabolism”, “cell adhesion molecules”, and “prion disease” (Figs. 6c–g and 7c–l).

## Discussion

In the present study, we demonstrated that ML models using combined Aβ soluble isoforms can predict downstream AD pathological processes, T+ and N+, in CU individuals with better results than Aβ isoforms independently. In the generated models, a higher AUC was achieved for predicting N+ when comparing with the T+. Our proteomics analysis identified several biological processes and signaling pathways altered at pre-symptomatic phase of AD. These findings are especially relevant when considering that AD pathological processes initiate around 20–30 years before the occurrence of the first clinical symptoms [18–22]. Finally, we identified DEPs among individuals wrongly classified as T+ by ML that can be further explored to improve prediction performance of the models.

The notion that Aβ triggers tau hyperphosphorylation and neurodegeneration has been corroborated by multiple experimental studies [23–26]. In fact, Höglund and colleagues demonstrated that CU individuals with amyloidosis presented increased levels of p-tau181 and t-tau in the CSF [27]. However, the diagnostic value of Aβ_1﻿–42_ has been explored in the literature delivering, though, only modest accuracy for AD prediction [28, 29]. Accordingly, here we demonstrated a poor AUC of 0.580 for N+ and 0.529 for T+ prediction modeled using the Aβ_1﻿–42_ isoform by itself, the most used CSF biomarker in the diagnosis of AD. *Per se*, the poorly explored isoform Aβ_1﻿–38_ (AUC of 0.847) along with Aβ_1﻿–40_ (AUC of 0.811) were the most accurate predictors for both T+ and N+, respectively. In clinical studies, the Aβ_1﻿–42_/Aβ_1﻿–38_ ratio has been capable of significantly discriminating AD from other forms of dementia [30–32] and shown to be negatively correlated with CSF p-tau levels in AD patients [31]. Additionally, a slight increase in Aβ_1–38_ levels was found in a disease-specific manner in the CSF of AD subjects [32, 33]. Nevertheless, a meta-analysis pointed no significant difference in Aβ_1–38_ levels between AD individuals and control group after comparing eight studies [34]. Cullen and colleagues more recently demonstrated that higher CSF Aβ_1﻿–38_ levels are negatively associated with cognitive decline and risk of developing AD [35]. In this context, it is evident that the potential of this isoform to add information in the preclinical stage of the disease remains under-explored.

In this work, we showed that a logistic regression model could predict T+ using multiple input features, with an AUC of 0.929. It has been demonstrated that Aβ dysmetabolism is capable of triggering the conversion from a normal to a toxic state of tau-dependent synaptic dysfunction [23]. As well, a synergistic interaction between Aβ and tau pathology is likely to occur in AD, rather than the sum of their independent effects [36–38]. Bilgel and colleagues showed that a higher baseline amyloid load in CU individuals was associated with steeper cognitive decline [39]. In parallel, we hereby demonstrated that amyloid isoforms levels can predict N+ in CU individuals with an AUC of 0.936 using a kNN model. The combination of Aβ isoforms, especially those including smaller Aβ isoforms, seems to help to deliver the best results to predict N+. Indeed, limited *in vivo* evidence shows significant correlations between Aβ_1﻿–42_ levels in the CSF and neurodegeneration in CU individuals [27]. On the other hand, the importance of Aβ_1﻿–42_ isoform as a toxic amyloid specie has been extensively demonstrated [23–26]. In the context of isoform production, literature indicates that Aβ_1﻿–38_ is partially formed by cleavage of the Aβ_1﻿–42_ isoform [40]. Also, it seems that no further cleavage of Aβ_1﻿–38_ occurs, resulting in a “more stable” isoform of Aβ, easier to detect [40]. One could argue that a more prominent amyloid dysmetabolism, with higher rates of cleavage of Aβ_1﻿–42_ into Aβ_1﻿–38_, might be a crucial process that seems to drive tau pathology and neurodegeneration. However, the already described [41] faster turnover of Aβ_1﻿–42_ might be accounting for its poor predictive value in our model. Accordingly, our model shows an important role for less explored Aβ isoforms as indicators of emerging tau pathology and neurodegeneration. In addition to CSF, AD blood biomarkers have been gaining attention in recent years [42]. Due to their scalability, blood biomarkers will generate large datasets highly suited for ML prediction models.

Aβ isoforms used in combination seems key for predicting T+ and N+, but do not completely explain all the aspects of AD downstream events. Thus, it is believed that simultaneous phenomena, that account for AD heterogeneity, are taking place in the brains of these individuals. In this context, CSF proteomics has been increasingly applied in the attempt to discover novel biomarkers for AD. However, it is mainly focused in comparing CU and AD individuals [43, 44]. Here, we showed Aβ pathology-dependent changes at protein level occurring in the CSF of CU individuals. Similarly, Whelan and colleagues performed a multiplex proteomics analysis in the CSF of CU A+ and A− patients and found two DEPs significantly altered: Chitinase 3-like protein (YKL-40) and SPARC-related modular calcium binding protein 2 (SMOC2) [45]. The great number of DEPs between CU T+ and T− subjects identified in our study allowed the further determination of biological processes and signaling pathways significantly enriched in these individuals. Additionally, significant differences in DEPs and its associated biological processes and signaling pathways were observed when comparing right and wrong ML predictions for T+. Interestingly, DEPs identified in other studies comparing CU and AD were also found in our analysis of ML wrong predictions for T+ [44]. In specific, YKL-40, SOD1, PKM, and glucose metabolism related proteins are among the DEPs found in both studies. The degree of similarity between studies seems to highlight a robust pattern of change rather than a cohort-specific effect. These results might shed light to key proteins that can be further explored to improve ML performance for predicting T+ and N+.

## Conclusions

Our findings indicate that the use of ML models with Aβ isoforms as input features might help to predict individuals with AD downstream pathology. In addition, CSF proteomics analysis highlighted a promising group of proteins potentially driving tau pathology, which can be further explored for improving future T+ and N+ prediction. Finally, the combination of methodologies used here—ML and proteomics—may help to further understand AD pathology heterogeneity.

## Supplementary Information

**Additional file 1.** Machine learning results for predicting tau pathology positivity (T+). Table containing features, AUC and standard deviation results for all 1023 models for predicting tau pathology positivity.

**Additional file 2.** Machine learning results for predicting neurodegeneration positivity (N+). Table containing features, AUC and standard deviation results for all 1023 models for predicting neurodegeneration positivity.

**Additional file 3.** Differentially expressed proteins (DEPs) in the cerebrospinal fluid (CSF) of cognitively unimpaired (CU) tau pathology positive (T+) compared to negative (T−) subjects. Table containing Protein ID, p-value, adjusted p-value, t-value and logFC for differentially expressed proteins in the cerebrospinal fluid of cognitively unimpaired tau pathology positive compared to negative subjects.

**Additional file 4. **Differentially expressed proteins (DEPs) in the cerebrospinal fluid (CSF) of cognitively unimpaired (CU) neurodegeneration positive (N+) compared to negative (N−) subjects. Table containing Protein ID, p-value, adjusted p-value, t-value and logFC for differentially expressed proteins in the cerebrospinal fluid of cognitively unimpaired neurodegeneration positive compared to negative subjects

## Data Availability

Data used in preparation of this article were obtained from the Alzheimer’s Disease Neuroimaging Initiative (ADNI) database. The dataset supporting the conclusions of this manuscript is available at the ADNI website (http://adni.loni.usc.edu/).

## Abbreviations

2D-UPLC-MS/MS: 2D-ultra-performance liquid chromatography-tandem mass spectrometry
A+: Amyloid-beta positivity
A−: Aβ negative
AD: Alzheimer's disease
ADAS-Cog: Alzheimer's Disease Assessment Scale-Cognitive Subscale
ADNI: Alzheimer's Disease Neuroimaging Initiative
AUC: Area under the curve
Aβ: Amyloid-beta
CSF: Cerebrospinal fluid
CU: Cognitively unimpaired
DEP: Differentially expressed proteins
GO: Gene ontology
JCTLM: Joint Committee for Traceability in Laboratory Medicine
ML: Machine learning
MMSE: Mini-Mental State Examination
MRI: Magnetic resonance imaging
N+: Neurodegeneration positivity
N−: Neurodegeneration negative
NIA-AA: National Institute on Aging-Alzheimer's Association
p-tau: Phosphorylated tau
PET: Positron emission tomography
RBM: Rules Based Medicine
SD: Standard deviation
SMOC2: SPARC-related modular calcium binding protein 2
T+: Tau pathology positivity
T−: Tau pathology negative
t-tau: Total tau
YKL-40: Chitinase 3-like protein
