# Supplementary material for: Soluble amyloid-beta isoforms predict downstream Alzheimer’s disease pathology
Source: Cell Biosci. 2021 Dec 11;11:204. doi: 10.1186/s13578-021-00712-3 (PMC8665586; doi:10.1186/s13578-021-00712-3)
Supplement: Supplementary file 1 — Additional file 1. Machine learning results for predicting tau pathology positivity (T+). Table containing features, AUC and standard deviation results for all 1023 models for predicting tau pathology positivity. [file 13578_2021_712_MOESM1_ESM.docx]

Additional file 1

M**achine learning results for predicting tau pathology positivity (T+).**

| **Features** | **Acc** | **Stdev** |
| --- | --- | --- |
| Aβ42, Aβ42/Aβ40, Aβ42/Aβ38, Aβ40/Aβ38, PTEDUCAT | 0.887 | 0.024 |
| Aβ42, Aβ42/Aβ40, Aβ40/Aβ38, PTEDUCAT | 0.893 | 0.030 |
| Aβ42, Aβ42/Aβ40, Aβ40/Aβ38, PTEDUCAT, APOE4 | 0.891 | 0.029 |
| Aβ42, Aβ42/Aβ40, Aβ42/Aβ38, Aβ40/Aβ38, PTEDUCAT, APOE4 | 0.884 | 0.023 |
| Aβ42, Aβ42/Aβ40, Aβ42/Aβ38, Aβ40/Aβ38, AGE, PTEDUCAT | 0.884 | 0.024 |
| Aβ42, Aβ42/Aβ40, Aβ42/Aβ38, PTEDUCAT, APOE4 | 0.890 | 0.030 |
| Aβ42, Aβ42/Aβ40, Aβ42/Aβ38, Aβ40/Aβ38, AGE, PTEDUCAT, APOE4 | 0.882 | 0.023 |
| Aβ42, Aβ42/Aβ40, Aβ42/Aβ38, Aβ40/Aβ38, AGE, PTEDUCAT, PTGENDER | 0.885 | 0.027 |
| Aβ40, Aβ42/Aβ40, Aβ40/Aβ38, AGE, PTEDUCAT | 0.880 | 0.021 |
| Aβ40, Aβ42/Aβ38, Aβ40/Aβ38, PTEDUCAT | 0.877 | 0.020 |
| Aβ42, Aβ40, Aβ42/Aβ40, Aβ40/Aβ38, PTEDUCAT | 0.887 | 0.030 |
| Aβ42, Aβ42/Aβ40, Aβ42/Aβ38, AGE, PTEDUCAT | 0.887 | 0.031 |
| Aβ42, Aβ40, Aβ38, Aβ42/Aβ40, Aβ40/Aβ38, PTEDUCAT | 0.877 | 0.021 |
| Aβ42, Aβ42/Aβ40, AGE, PTEDUCAT, APOE4 | 0.886 | 0.031 |
| Aβ42, Aβ42/Aβ40, Aβ40/Aβ38, AGE, PTEDUCAT, APOE4 | 0.888 | 0.032 |
| Aβ42, Aβ42/Aβ40, Aβ42/Aβ38, AGE, PTEDUCAT, APOE4 | 0.887 | 0.032 |
| Aβ42, Aβ42/Aβ40, PTEDUCAT, APOE4 | 0.887 | 0.032 |
| Aβ42, Aβ42/Aβ40, Aβ40/Aβ38, AGE, PTEDUCAT | 0.888 | 0.034 |
| Aβ42, Aβ42/Aβ40, Aβ40/Aβ38, AGE, PTEDUCAT, PTGENDER, APOE4 | 0.886 | 0.032 |
| Aβ42, Aβ42/Aβ40, Aβ42/Aβ38, PTEDUCAT | 0.890 | 0.036 |
| Aβ42, Aβ42/Aβ40, PTEDUCAT | 0.888 | 0.034 |
| Aβ42, Aβ42/Aβ40, AGE, PTEDUCAT | 0.883 | 0.030 |
| Aβ42, Aβ42/Aβ40, Aβ40/Aβ38, AGE, PTEDUCAT, PTGENDER | 0.888 | 0.035 |
| Aβ42, Aβ42/Aβ40, Aβ42/Aβ38, Aβ40/Aβ38, PTEDUCAT, PTGENDER, APOE4 | 0.879 | 0.025 |
| Aβ42, Aβ40, Aβ38 | 0.872 | 0.019 |
| Aβ42, Aβ42/Aβ40, Aβ42/Aβ38, Aβ40/Aβ38, PTEDUCAT, PTGENDER | 0.880 | 0.028 |
| Aβ42, Aβ42/Aβ40, Aβ40/Aβ38, PTEDUCAT, PTGENDER, APOE4 | 0.885 | 0.033 |
| Aβ42, Aβ38, Aβ42/Aβ40, Aβ42/Aβ38, Aβ40/Aβ38, PTEDUCAT | 0.875 | 0.023 |
| Aβ42, Aβ42/Aβ40, Aβ42/Aβ38, PTEDUCAT, PTGENDER | 0.879 | 0.027 |
| Aβ42, Aβ42/Aβ40, Aβ42/Aβ38, AGE, PTEDUCAT, PTGENDER | 0.883 | 0.031 |
| Aβ40, Aβ38, Aβ42/Aβ40, Aβ42/Aβ38, Aβ40/Aβ38, AGE, PTEDUCAT | 0.880 | 0.028 |
| Aβ42, Aβ40, Aβ38, Aβ42/Aβ40, Aβ42/Aβ38, Aβ40/Aβ38, AGE, PTEDUCAT | 0.876 | 0.025 |
| Aβ40, Aβ38, Aβ42/Aβ40, Aβ42/Aβ38, Aβ40/Aβ38, PTEDUCAT | 0.879 | 0.028 |
| Aβ42, Aβ42/Aβ40, Aβ40/Aβ38, PTEDUCAT, PTGENDER | 0.886 | 0.035 |
| Aβ40, Aβ42/Aβ40, Aβ40/Aβ38, PTEDUCAT | 0.874 | 0.024 |
| Aβ40, Aβ42/Aβ38, Aβ40/Aβ38, AGE, PTEDUCAT | 0.872 | 0.022 |
| Aβ40, Aβ38, Aβ42/Aβ40, Aβ42/Aβ38, PTEDUCAT | 0.883 | 0.034 |
| Aβ42, Aβ38, Aβ42/Aβ40, PTEDUCAT, APOE4 | 0.881 | 0.033 |
| Aβ42, Aβ40, Aβ38, Aβ42/Aβ40, AGE, PTEDUCAT | 0.881 | 0.032 |
| Aβ42, Aβ42/Aβ40, PTEDUCAT, PTGENDER, APOE4 | 0.883 | 0.035 |
| Aβ40, Aβ38, Aβ42/Aβ38 | 0.875 | 0.027 |
| Aβ42, Aβ40, Aβ42/Aβ40, Aβ42/Aβ38, Aβ40/Aβ38, AGE, PTEDUCAT | 0.876 | 0.028 |
| Aβ42, Aβ40, Aβ38, Aβ42/Aβ40, Aβ40/Aβ38, AGE, PTEDUCAT, PTGENDER | 0.875 | 0.028 |
| Aβ42, Aβ38, Aβ42/Aβ40, PTEDUCAT | 0.883 | 0.036 |
| Aβ42, Aβ38, Aβ42/Aβ40, Aβ42/Aβ38, AGE, PTEDUCAT | 0.882 | 0.035 |
| Aβ40, Aβ38, Aβ42/Aβ40, Aβ42/Aβ38, Aβ40/Aβ38, AGE, PTEDUCAT, PTGENDER | 0.878 | 0.031 |
| Aβ40, Aβ42/Aβ38, PTEDUCAT | 0.883 | 0.036 |
| Aβ42, Aβ40, Aβ38, Aβ42/Aβ40, Aβ42/Aβ38, Aβ40/Aβ38 | 0.874 | 0.027 |
| Aβ40, Aβ42/Aβ38, Aβ40/Aβ38 | 0.867 | 0.021 |
| Aβ42, Aβ40, Aβ38, Aβ42/Aβ40 | 0.881 | 0.034 |
| Aβ42, Aβ40, Aβ38, Aβ42/Aβ40, Aβ42/Aβ38, Aβ40/Aβ38, AGE, PTEDUCAT, APOE4 | 0.876 | 0.030 |
| Aβ42, Aβ40, Aβ38, Aβ42/Aβ38 | 0.876 | 0.030 |
| Aβ42, Aβ40, Aβ38, Aβ42/Aβ40, Aβ42/Aβ38, Aβ40/Aβ38, AGE, PTEDUCAT, PTGENDER | 0.874 | 0.028 |
| Aβ40, Aβ38, Aβ42/Aβ40, Aβ42/Aβ38, Aβ40/Aβ38 | 0.883 | 0.037 |
| Aβ42, Aβ42/Aβ40, Aβ42/Aβ38, Aβ40/Aβ38, AGE, PTEDUCAT, PTGENDER, APOE4 | 0.873 | 0.026 |
| Aβ42, Aβ40, Aβ38, Aβ42/Aβ40, Aβ42/Aβ38, Aβ40/Aβ38, PTEDUCAT | 0.874 | 0.028 |
| Aβ42, Aβ40, Aβ38, Aβ42/Aβ40, Aβ40/Aβ38, PTEDUCAT, PTGENDER, APOE4 | 0.870 | 0.025 |
| Aβ42, Aβ42/Aβ40, Aβ42/Aβ38, PTEDUCAT, PTGENDER, APOE4 | 0.882 | 0.036 |
| Aβ40, Aβ42/Aβ40, Aβ40/Aβ38 | 0.874 | 0.029 |
| Aβ40, Aβ42/Aβ40, PTEDUCAT | 0.882 | 0.037 |
| Aβ38, Aβ42/Aβ40, Aβ40/Aβ38, PTEDUCAT | 0.868 | 0.023 |
| Aβ40, Aβ42/Aβ40, Aβ42/Aβ38, Aβ40/Aβ38, AGE, PTEDUCAT | 0.875 | 0.030 |
| Aβ42, Aβ38, Aβ42/Aβ40, Aβ40/Aβ38, PTEDUCAT, APOE4 | 0.880 | 0.035 |
| Aβ42, Aβ42/Aβ38, Aβ40/Aβ38, AGE, PTEDUCAT | 0.873 | 0.029 |
| Aβ42, Aβ40, Aβ42/Aβ40, Aβ40/Aβ38, AGE, PTEDUCAT | 0.880 | 0.035 |
| Aβ38, Aβ42/Aβ40, Aβ42/Aβ38, Aβ40/Aβ38, PTEDUCAT | 0.870 | 0.025 |
| Aβ42, Aβ40, Aβ38, Aβ42/Aβ38, Aβ40/Aβ38, AGE, PTEDUCAT | 0.879 | 0.035 |
| Aβ42, Aβ40, Aβ42/Aβ40, PTEDUCAT | 0.881 | 0.037 |
| Aβ42, Aβ40, Aβ38, Aβ42/Aβ38, Aβ40/Aβ38, PTEDUCAT | 0.874 | 0.030 |
| Aβ42, Aβ42/Aβ40, Aβ42/Aβ38, AGE, PTEDUCAT, PTGENDER, APOE4 | 0.881 | 0.037 |
| Aβ38, Aβ42/Aβ40, Aβ42/Aβ38, Aβ40/Aβ38, AGE, PTEDUCAT | 0.874 | 0.031 |
| Aβ42, Aβ40, Aβ38, Aβ42/Aβ40, Aβ40/Aβ38, PTEDUCAT, APOE4 | 0.879 | 0.035 |
| Aβ42, Aβ40, Aβ38, Aβ42/Aβ40, Aβ42/Aβ38, PTEDUCAT | 0.878 | 0.034 |
| Aβ40, Aβ38, Aβ42/Aβ40, Aβ40/Aβ38, AGE, PTEDUCAT | 0.864 | 0.021 |
| Aβ42, Aβ40, Aβ38, Aβ42/Aβ40, Aβ42/Aβ38, AGE, PTEDUCAT | 0.876 | 0.033 |
| Aβ42, Aβ40, Aβ38, Aβ42/Aβ40, Aβ40/Aβ38, AGE, PTEDUCAT | 0.880 | 0.037 |
| Aβ42, Aβ40, Aβ42/Aβ38, PTEDUCAT | 0.871 | 0.028 |
| Aβ38, Aβ42/Aβ40, Aβ40/Aβ38 | 0.872 | 0.030 |
| Aβ42, Aβ38, Aβ42/Aβ40, Aβ40/Aβ38, PTEDUCAT | 0.881 | 0.038 |
| Aβ40, Aβ38, Aβ42/Aβ38, APOE4 | 0.871 | 0.029 |
| Aβ42, Aβ42/Aβ40, AGE, PTEDUCAT, PTGENDER, APOE4 | 0.881 | 0.039 |
| Aβ38, Aβ42/Aβ38, Aβ40/Aβ38 | 0.873 | 0.031 |
| Aβ38, Aβ42/Aβ40, Aβ42/Aβ38, PTEDUCAT | 0.874 | 0.032 |
| Aβ42, Aβ38, Aβ42/Aβ40, Aβ40/Aβ38, AGE, PTEDUCAT | 0.876 | 0.034 |
| Aβ40, Aβ38, Aβ42/Aβ40 | 0.876 | 0.034 |
| Aβ40, Aβ42/Aβ38 | 0.881 | 0.040 |
| Aβ42, Aβ40, Aβ38, Aβ42/Aβ40, Aβ42/Aβ38, Aβ40/Aβ38, PTEDUCAT, APOE4 | 0.874 | 0.032 |
| Aβ42, Aβ42/Aβ40, AGE, PTEDUCAT, PTGENDER | 0.876 | 0.034 |
| Aβ40, Aβ38, Aβ42/Aβ38, Aβ40/Aβ38, AGE, PTEDUCAT | 0.862 | 0.020 |
| Aβ42, Aβ38, Aβ42/Aβ40, Aβ42/Aβ38, Aβ40/Aβ38, AGE, PTEDUCAT, PTGENDER | 0.872 | 0.030 |
| Aβ40, Aβ38, Aβ42/Aβ40, Aβ40/Aβ38, PTEDUCAT | 0.864 | 0.022 |
| Aβ42, Aβ40, Aβ38, Aβ42/Aβ40, PTEDUCAT | 0.864 | 0.023 |
| Aβ42, Aβ40, Aβ42/Aβ40, Aβ42/Aβ38, Aβ40/Aβ38, PTEDUCAT | 0.874 | 0.032 |
| Aβ40, Aβ38, Aβ42/Aβ40, PTEDUCAT | 0.873 | 0.031 |
| Aβ42, Aβ38, Aβ42/Aβ40, Aβ42/Aβ38, PTEDUCAT | 0.879 | 0.038 |
| Aβ40, Aβ38, Aβ42/Aβ40, AGE, PTEDUCAT | 0.877 | 0.036 |
| Aβ42, Aβ38, Aβ42/Aβ40, AGE, PTEDUCAT | 0.882 | 0.041 |
| Aβ42, Aβ42/Aβ40, Aβ42/Aβ38, Aβ40/Aβ38, APOE4 | 0.870 | 0.029 |
| Aβ42, Aβ40, Aβ38, Aβ42/Aβ40, AGE | 0.875 | 0.034 |
| Aβ42, Aβ40, Aβ38, Aβ42/Aβ40, Aβ42/Aβ38, Aβ40/Aβ38, AGE | 0.870 | 0.030 |
| Aβ42, Aβ38, Aβ42/Aβ40, Aβ40/Aβ38, AGE, PTEDUCAT, PTGENDER | 0.877 | 0.036 |
| Aβ42, Aβ42/Aβ38, Aβ40/Aβ38, PTEDUCAT, APOE4 | 0.877 | 0.036 |
| Aβ42, Aβ42/Aβ40, PTEDUCAT, PTGENDER | 0.882 | 0.042 |
| Aβ40, Aβ38, Aβ42/Aβ38, Aβ40/Aβ38, PTEDUCAT | 0.860 | 0.020 |
| Aβ42, Aβ38, Aβ42/Aβ40, AGE, PTEDUCAT, APOE4 | 0.873 | 0.033 |
| Aβ42, Aβ38, Aβ42/Aβ40, Aβ42/Aβ38, Aβ40/Aβ38, AGE, PTEDUCAT, APOE4 | 0.870 | 0.029 |
| Aβ42, Aβ40, Aβ42/Aβ40, Aβ42/Aβ38, AGE, PTEDUCAT | 0.878 | 0.038 |
| Aβ40, Aβ38, Aβ42/Aβ40, Aβ42/Aβ38, AGE, PTEDUCAT | 0.879 | 0.039 |
| Aβ40, Aβ38, Aβ42/Aβ40, Aβ40/Aβ38 | 0.873 | 0.033 |
| Aβ42, Aβ40, Aβ38, Aβ42/Aβ38, Aβ40/Aβ38, AGE | 0.880 | 0.040 |
| Aβ42, Aβ42/Aβ40, Aβ42/Aβ38, Aβ40/Aβ38 | 0.872 | 0.032 |
| Aβ40, Aβ42/Aβ40, AGE, PTEDUCAT | 0.880 | 0.040 |
| Aβ42, Aβ40, Aβ38, Aβ42/Aβ40, Aβ42/Aβ38, Aβ40/Aβ38, AGE, PTEDUCAT, PTGENDER, APOE4 | 0.874 | 0.034 |
| Aβ42, Aβ40, Aβ42/Aβ40, AGE | 0.877 | 0.037 |
| Aβ42, Aβ40, Aβ42/Aβ38, Aβ40/Aβ38, AGE, APOE4 | 0.884 | 0.044 |
| Aβ42, Aβ38, Aβ42/Aβ40, Aβ40/Aβ38, AGE, PTEDUCAT, PTGENDER, APOE4 | 0.878 | 0.038 |
| Aβ42, Aβ40, Aβ42/Aβ40, PTEDUCAT, APOE4 | 0.876 | 0.037 |
| Aβ40, Aβ42/Aβ38, PTEDUCAT, APOE4 | 0.879 | 0.039 |
| Aβ42, Aβ42/Aβ38, Aβ40/Aβ38, AGE, APOE4 | 0.861 | 0.021 |
| Aβ40, Aβ38, Aβ42/Aβ40, Aβ42/Aβ38, Aβ40/Aβ38, AGE | 0.873 | 0.034 |
| Aβ40, Aβ38, Aβ42/Aβ38, Aβ40/Aβ38 | 0.875 | 0.035 |
| Aβ38, Aβ42/Aβ40, PTEDUCAT | 0.878 | 0.039 |
| Aβ42, Aβ40, Aβ42/Aβ40, Aβ40/Aβ38, PTEDUCAT, APOE4 | 0.876 | 0.037 |
| Aβ42, Aβ40, Aβ42/Aβ40, Aβ42/Aβ38, Aβ40/Aβ38, PTEDUCAT, APOE4 | 0.871 | 0.032 |
| Aβ42, Aβ38, Aβ42/Aβ40, Aβ42/Aβ38, Aβ40/Aβ38, PTEDUCAT, APOE4 | 0.866 | 0.027 |
| Aβ42, Aβ40, Aβ42/Aβ40, AGE, PTEDUCAT | 0.878 | 0.040 |
| Aβ42, Aβ42/Aβ38, Aβ40/Aβ38, PTEDUCAT | 0.876 | 0.037 |
| Aβ40, Aβ38, Aβ42/Aβ40, Aβ42/Aβ38, Aβ40/Aβ38, AGE, PTEDUCAT, APOE4 | 0.874 | 0.035 |
| Aβ40, Aβ38, Aβ42/Aβ40, Aβ42/Aβ38, Aβ40/Aβ38, PTEDUCAT, PTGENDER | 0.874 | 0.035 |
| Aβ42, Aβ38, Aβ42/Aβ40, Aβ42/Aβ38, Aβ40/Aβ38, AGE, PTEDUCAT | 0.870 | 0.032 |
| Aβ42, Aβ40, Aβ38, PTEDUCAT | 0.861 | 0.022 |
| Aβ42, Aβ40, Aβ38, Aβ42/Aβ40, Aβ40/Aβ38, PTEDUCAT, PTGENDER | 0.870 | 0.031 |
| Aβ42, Aβ38, Aβ42/Aβ40, Aβ40/Aβ38, PTEDUCAT, PTGENDER | 0.878 | 0.039 |
| Aβ42, Aβ38, Aβ42/Aβ40, Aβ40/Aβ38, PTEDUCAT, PTGENDER, APOE4 | 0.876 | 0.038 |
| Aβ40, Aβ42/Aβ38, Aβ40/Aβ38, AGE | 0.863 | 0.025 |
| Aβ40, Aβ42/Aβ38, Aβ40/Aβ38, AGE, PTEDUCAT, APOE4 | 0.875 | 0.037 |
| Aβ42, Aβ40, Aβ38, Aβ42/Aβ40, Aβ40/Aβ38 | 0.881 | 0.043 |
| Aβ42, Aβ40, Aβ38, Aβ42/Aβ40, Aβ42/Aβ38, Aβ40/Aβ38, PTEDUCAT, PTGENDER | 0.871 | 0.033 |
| Aβ42, Aβ40, Aβ38, Aβ42/Aβ38, AGE, PTEDUCAT | 0.881 | 0.043 |
| Aβ42, Aβ40, Aβ42/Aβ40, Aβ40/Aβ38, PTEDUCAT, PTGENDER | 0.878 | 0.040 |
| Aβ42, Aβ40, Aβ42/Aβ40, Aβ40/Aβ38, APOE4 | 0.867 | 0.029 |
| Aβ42, Aβ38, Aβ42/Aβ40, Aβ42/Aβ38, Aβ40/Aβ38, PTEDUCAT, PTGENDER | 0.870 | 0.031 |
| Aβ42, Aβ40, Aβ42/Aβ40, Aβ42/Aβ38, Aβ40/Aβ38, PTEDUCAT, PTGENDER | 0.870 | 0.031 |
| Aβ42, Aβ42/Aβ38, Aβ40/Aβ38, AGE, PTEDUCAT, PTGENDER | 0.862 | 0.024 |
| Aβ42, Aβ40, Aβ38, Aβ42/Aβ40, AGE, PTEDUCAT, PTGENDER | 0.878 | 0.040 |
| Aβ42, Aβ40, Aβ42/Aβ38, Aβ40/Aβ38, AGE, PTGENDER, APOE4 | 0.883 | 0.045 |
| Aβ40, Aβ42/Aβ40 | 0.879 | 0.041 |
| Aβ42, Aβ40, Aβ38, Aβ42/Aβ40, Aβ40/Aβ38, APOE4 | 0.878 | 0.040 |
| Aβ40, Aβ38, Aβ42/Aβ40, Aβ42/Aβ38, PTEDUCAT, PTGENDER | 0.878 | 0.040 |
| Aβ42, Aβ40, Aβ42/Aβ40, Aβ40/Aβ38, AGE, PTEDUCAT, PTGENDER | 0.879 | 0.041 |
| Aβ42, Aβ40, Aβ42/Aβ40, Aβ42/Aβ38, Aβ40/Aβ38, AGE | 0.870 | 0.032 |
| Aβ40, Aβ42/Aβ38, AGE, PTEDUCAT | 0.878 | 0.040 |
| Aβ42, Aβ40, Aβ42/Aβ40, Aβ42/Aβ38, PTEDUCAT, PTGENDER | 0.866 | 0.028 |
| Aβ42, Aβ40, Aβ42/Aβ40, Aβ40/Aβ38, PTEDUCAT, PTGENDER, APOE4 | 0.875 | 0.038 |
| Aβ42, Aβ40, Aβ42/Aβ40, Aβ42/Aβ38, PTEDUCAT | 0.877 | 0.040 |
| Aβ40, Aβ38, Aβ42/Aβ40, AGE, PTEDUCAT, APOE4 | 0.876 | 0.039 |
| Aβ42, Aβ40, Aβ42/Aβ38 | 0.877 | 0.040 |
| Aβ42, Aβ40, Aβ38, Aβ42/Aβ38, PTEDUCAT | 0.862 | 0.025 |
| Aβ42, Aβ40, AGE | 0.873 | 0.036 |
| Aβ42, Aβ40, Aβ38, Aβ42/Aβ40, Aβ42/Aβ38, AGE, PTEDUCAT, APOE4 | 0.876 | 0.039 |
| Aβ42, Aβ40, Aβ38, AGE, PTEDUCAT | 0.869 | 0.032 |
| Aβ42, Aβ42/Aβ40 | 0.878 | 0.041 |
| Aβ40, Aβ38, Aβ42/Aβ38, AGE, PTEDUCAT, PTGENDER | 0.872 | 0.035 |
| Aβ42, Aβ42/Aβ40, AGE | 0.877 | 0.041 |
| Aβ42, Aβ40, Aβ42/Aβ40, Aβ42/Aβ38, Aβ40/Aβ38, AGE, PTEDUCAT, PTGENDER | 0.869 | 0.032 |
| Aβ42, Aβ42/Aβ38, Aβ40/Aβ38, AGE | 0.880 | 0.043 |
| Aβ42, Aβ42/Aβ40, APOE4 | 0.876 | 0.040 |
| Aβ42, Aβ40, Aβ42/Aβ38, Aβ40/Aβ38, APOE4 | 0.853 | 0.016 |
| Aβ40, Aβ38, Aβ42/Aβ40, Aβ42/Aβ38, Aβ40/Aβ38, PTEDUCAT, APOE4 | 0.874 | 0.037 |
| Aβ42, Aβ40, Aβ42/Aβ40, Aβ42/Aβ38, AGE | 0.876 | 0.040 |
| Aβ42, Aβ40, Aβ38, Aβ42/Aβ40, Aβ42/Aβ38, AGE, PTEDUCAT, PTGENDER | 0.873 | 0.036 |
| Aβ42, Aβ40, Aβ42/Aβ38, Aβ40/Aβ38 | 0.867 | 0.030 |
| Aβ42, Aβ40, Aβ38, Aβ42/Aβ40, PTEDUCAT, APOE4 | 0.876 | 0.039 |
| Aβ42, Aβ40, Aβ38, Aβ42/Aβ40, Aβ42/Aβ38, PTEDUCAT, APOE4 | 0.876 | 0.039 |
| Aβ42, Aβ40, Aβ42/Aβ38, AGE | 0.875 | 0.039 |
| Aβ42, Aβ40, Aβ38, Aβ42/Aβ40, Aβ42/Aβ38, Aβ40/Aβ38, APOE4 | 0.871 | 0.035 |
| Aβ42, Aβ40, Aβ42/Aβ40, Aβ40/Aβ38 | 0.874 | 0.038 |
| Aβ42, Aβ40, Aβ42/Aβ40, Aβ42/Aβ38, PTEDUCAT, APOE4 | 0.875 | 0.039 |
| Aβ42, Aβ40, Aβ42/Aβ38, Aβ40/Aβ38, AGE, PTEDUCAT | 0.865 | 0.029 |
| Aβ42, Aβ40, Aβ38, Aβ42/Aβ40, Aβ42/Aβ38, Aβ40/Aβ38, AGE, APOE4 | 0.871 | 0.035 |
| Aβ42, Aβ40, Aβ38, Aβ40/Aβ38, AGE, PTEDUCAT | 0.876 | 0.040 |
| Aβ42, Aβ42/Aβ38, Aβ40/Aβ38, PTGENDER, APOE4 | 0.850 | 0.014 |
| Aβ42, Aβ40, Aβ42/Aβ40 | 0.874 | 0.038 |
| Aβ42, Aβ40, Aβ38, Aβ42/Aβ38, Aβ40/Aβ38 | 0.853 | 0.018 |
| Aβ42, Aβ40, Aβ38, Aβ42/Aβ40, Aβ40/Aβ38, AGE | 0.868 | 0.032 |
| Aβ40, Aβ38, Aβ42/Aβ38, PTEDUCAT | 0.863 | 0.027 |
| Aβ42, Aβ38, Aβ42/Aβ38, Aβ40/Aβ38, PTGENDER | 0.872 | 0.036 |
| Aβ42, Aβ38, Aβ42/Aβ40, Aβ42/Aβ38, Aβ40/Aβ38, PTEDUCAT, PTGENDER, APOE4 | 0.866 | 0.030 |
| Aβ40, Aβ42/Aβ38, Aβ40/Aβ38, PTEDUCAT, APOE4 | 0.869 | 0.034 |
| Aβ42, Aβ40, Aβ38, Aβ42/Aβ40, Aβ42/Aβ38, Aβ40/Aβ38, AGE, PTGENDER | 0.868 | 0.033 |
| Aβ42, Aβ40, Aβ42/Aβ40, Aβ40/Aβ38, AGE, PTEDUCAT, APOE4 | 0.876 | 0.041 |
| Aβ42, Aβ38, Aβ42/Aβ40, AGE, PTEDUCAT, PTGENDER | 0.876 | 0.041 |
| Aβ42, Aβ40, Aβ38, Aβ42/Aβ40, Aβ42/Aβ38 | 0.872 | 0.037 |
| Aβ40, Aβ42/Aβ40, Aβ40/Aβ38, AGE, APOE4 | 0.859 | 0.024 |
| Aβ42, Aβ40, Aβ38, AGE | 0.860 | 0.025 |
| Aβ42, Aβ42/Aβ40, Aβ42/Aβ38, Aβ40/Aβ38, AGE | 0.869 | 0.033 |
| Aβ42, Aβ40 | 0.870 | 0.035 |
| Aβ38, Aβ42/Aβ40, Aβ40/Aβ38, AGE, PTEDUCAT | 0.866 | 0.031 |
| Aβ42, Aβ42/Aβ38, APOE4 | 0.854 | 0.019 |
| Aβ42, Aβ40, Aβ38, Aβ42/Aβ40, PTEDUCAT, PTGENDER | 0.876 | 0.041 |
| Aβ40, Aβ38, Aβ42/Aβ40, PTGENDER | 0.874 | 0.039 |
| Aβ40, Aβ42/Aβ38, Aβ40/Aβ38, APOE4 | 0.868 | 0.033 |
| Aβ42, Aβ42/Aβ38, Aβ40/Aβ38, AGE, PTEDUCAT, APOE4 | 0.876 | 0.041 |
| Aβ42, Aβ42/Aβ40, AGE, PTGENDER | 0.872 | 0.037 |
| Aβ40, Aβ38, Aβ42/Aβ40, Aβ42/Aβ38, Aβ40/Aβ38, AGE, PTGENDER | 0.870 | 0.036 |
| Aβ40, Aβ42/Aβ40, Aβ40/Aβ38, APOE4 | 0.866 | 0.031 |
| Aβ42, Aβ38, Aβ42/Aβ40, Aβ42/Aβ38, PTEDUCAT, APOE4 | 0.870 | 0.035 |
| Aβ38, Aβ42/Aβ40, Aβ40/Aβ38, APOE4 | 0.869 | 0.034 |
| Aβ40, Aβ38, Aβ42/Aβ40, Aβ42/Aβ38 | 0.876 | 0.042 |
| Aβ42, Aβ38, Aβ42/Aβ40, PTEDUCAT, PTGENDER, APOE4 | 0.875 | 0.040 |
| Aβ40, Aβ38, Aβ42/Aβ40, Aβ40/Aβ38, AGE | 0.855 | 0.020 |
| Aβ42, Aβ38, Aβ42/Aβ40, Aβ40/Aβ38, AGE, PTEDUCAT, APOE4 | 0.874 | 0.040 |
| Aβ40, Aβ38, Aβ42/Aβ40, Aβ40/Aβ38, PTEDUCAT, APOE4 | 0.863 | 0.029 |
| Aβ42, Aβ40, Aβ38, Aβ42/Aβ40, Aβ42/Aβ38, AGE | 0.871 | 0.037 |
| Aβ42, Aβ40, Aβ38, Aβ42/Aβ40, Aβ42/Aβ38, PTEDUCAT, PTGENDER | 0.874 | 0.040 |
| Aβ42, Aβ40, Aβ42/Aβ38, Aβ40/Aβ38, PTEDUCAT | 0.868 | 0.034 |
| Aβ40, Aβ42/Aβ40, PTEDUCAT, APOE4 | 0.876 | 0.042 |
| Aβ40, Aβ38, Aβ42/Aβ38, Aβ40/Aβ38, AGE, PTEDUCAT, PTGENDER | 0.856 | 0.022 |
| Aβ40, Aβ38, Aβ42/Aβ40, PTEDUCAT, PTGENDER | 0.867 | 0.033 |
| Aβ42, Aβ38, Aβ42/Aβ40, Aβ42/Aβ38, PTEDUCAT, PTGENDER | 0.875 | 0.041 |
| Aβ42, Aβ40, Aβ42/Aβ38, Aβ40/Aβ38, AGE, PTGENDER | 0.878 | 0.044 |
| Aβ42, Aβ40, Aβ42/Aβ40, Aβ42/Aβ38, AGE, PTEDUCAT, PTGENDER | 0.876 | 0.042 |
| Aβ42, Aβ42/Aβ40, Aβ40/Aβ38, APOE4 | 0.875 | 0.041 |
| Aβ40, Aβ38, Aβ42/Aβ38, AGE, PTEDUCAT | 0.865 | 0.032 |
| Aβ42, Aβ38, Aβ42/Aβ38, Aβ40/Aβ38, AGE, PTEDUCAT | 0.873 | 0.039 |
| Aβ42, Aβ38, Aβ42/Aβ40, Aβ42/Aβ38, Aβ40/Aβ38, AGE | 0.865 | 0.031 |
| Aβ42, Aβ42/Aβ40, Aβ40/Aβ38 | 0.876 | 0.042 |
| Aβ42, Aβ42/Aβ40, AGE, APOE4 | 0.863 | 0.030 |
| Aβ40, Aβ38, Aβ42/Aβ40, Aβ42/Aβ38, AGE, PTEDUCAT, PTGENDER | 0.876 | 0.042 |
| Aβ42, Aβ40, PTEDUCAT | 0.872 | 0.039 |
| Aβ42, Aβ40, Aβ38, Aβ42/Aβ40, Aβ40/Aβ38, PTGENDER | 0.867 | 0.034 |
| Aβ42, Aβ38, Aβ42/Aβ40, PTEDUCAT, PTGENDER | 0.875 | 0.041 |
| Aβ42, Aβ42/Aβ40, Aβ42/Aβ38, Aβ40/Aβ38, AGE, APOE4 | 0.869 | 0.036 |
| Aβ42, Aβ40, Aβ42/Aβ40, AGE, PTEDUCAT, APOE4 | 0.873 | 0.040 |
| Aβ40, Aβ38, Aβ42/Aβ40, PTEDUCAT, APOE4 | 0.865 | 0.032 |
| Aβ42, Aβ40, Aβ42/Aβ40, Aβ42/Aβ38, AGE, PTEDUCAT, APOE4 | 0.874 | 0.041 |
| Aβ42, Aβ38, Aβ42/Aβ38, Aβ40/Aβ38, PTGENDER, APOE4 | 0.865 | 0.032 |
| Aβ40, Aβ38, Aβ42/Aβ38, Aβ40/Aβ38, AGE, PTEDUCAT, APOE4 | 0.861 | 0.028 |
| Aβ42, Aβ40, Aβ42/Aβ38, Aβ40/Aβ38, PTEDUCAT, APOE4 | 0.864 | 0.031 |
| Aβ40, Aβ38, Aβ42/Aβ38, Aβ40/Aβ38, AGE | 0.854 | 0.021 |
| Aβ42, Aβ40, Aβ42/Aβ40, Aβ42/Aβ38 | 0.873 | 0.040 |
| Aβ40, Aβ38, Aβ42/Aβ40, Aβ42/Aβ38, AGE | 0.877 | 0.044 |
| Aβ40, Aβ42/Aβ40, Aβ40/Aβ38, AGE | 0.865 | 0.033 |
| Aβ42, Aβ40, Aβ42/Aβ40, Aβ42/Aβ38, Aβ40/Aβ38, PTEDUCAT, PTGENDER, APOE4 | 0.866 | 0.034 |
| Aβ40, Aβ38, Aβ42/Aβ40, Aβ40/Aβ38, AGE, PTEDUCAT, APOE4 | 0.873 | 0.040 |
| Aβ42, Aβ40, Aβ42/Aβ40, Aβ42/Aβ38, Aβ40/Aβ38, AGE, PTEDUCAT, APOE4 | 0.863 | 0.031 |
| Aβ40, Aβ42/Aβ38, AGE | 0.869 | 0.036 |
| Aβ42, Aβ42/Aβ38, AGE, PTEDUCAT, APOE4 | 0.867 | 0.035 |
| Aβ40, Aβ38, Aβ42/Aβ38, Aβ40/Aβ38, PTEDUCAT, APOE4 | 0.861 | 0.029 |
| Aβ42, Aβ42/Aβ40, Aβ42/Aβ38 | 0.872 | 0.040 |
| Aβ42, Aβ40, Aβ42/Aβ38, AGE, PTEDUCAT | 0.874 | 0.042 |
| Aβ40, Aβ38, Aβ42/Aβ40, Aβ42/Aβ38, PTEDUCAT, APOE4 | 0.856 | 0.024 |
| Aβ42, Aβ40, Aβ38, Aβ42/Aβ40, Aβ40/Aβ38, AGE, PTEDUCAT, APOE4 | 0.872 | 0.040 |
| Aβ42, Aβ38, Aβ42/Aβ40, Aβ42/Aβ38, AGE, PTEDUCAT, PTGENDER | 0.871 | 0.039 |
| Aβ40, Aβ38, Aβ42/Aβ40, Aβ42/Aβ38, Aβ40/Aβ38, AGE, PTEDUCAT, PTGENDER, APOE4 | 0.871 | 0.039 |
| Aβ42, Aβ42/Aβ38, PTEDUCAT | 0.870 | 0.038 |
| Aβ42, Aβ38, Aβ42/Aβ40, Aβ42/Aβ38, AGE, PTEDUCAT, PTGENDER, APOE4 | 0.867 | 0.035 |
| Aβ42, Aβ40, Aβ38, Aβ42/Aβ40, Aβ42/Aβ38, Aβ40/Aβ38, PTEDUCAT, PTGENDER, APOE4 | 0.870 | 0.038 |
| Aβ38, Aβ42/Aβ40, Aβ42/Aβ38 | 0.865 | 0.033 |
| Aβ42, Aβ38, Aβ42/Aβ40, Aβ42/Aβ38, PTEDUCAT, PTGENDER, APOE4 | 0.873 | 0.041 |
| Aβ40, Aβ42/Aβ40, APOE4 | 0.869 | 0.037 |
| Aβ42, Aβ40, Aβ42/Aβ38, AGE, PTEDUCAT, APOE4 | 0.871 | 0.039 |
| Aβ40, Aβ38, Aβ42/Aβ40, Aβ42/Aβ38, AGE, PTGENDER | 0.876 | 0.044 |
| Aβ42, Aβ40, Aβ38, Aβ42/Aβ40, Aβ40/Aβ38, AGE, PTGENDER | 0.864 | 0.033 |
| Aβ42, Aβ40, Aβ38, Aβ42/Aβ40, Aβ42/Aβ38, Aβ40/Aβ38, PTGENDER | 0.868 | 0.036 |
| Aβ42, Aβ40, Aβ42/Aβ40, PTEDUCAT, PTGENDER | 0.875 | 0.043 |
| Aβ42, Aβ40, Aβ42/Aβ40, Aβ42/Aβ38, Aβ40/Aβ38, APOE4 | 0.850 | 0.018 |
| Aβ40, Aβ42/Aβ40, Aβ42/Aβ38, PTEDUCAT, APOE4 | 0.866 | 0.035 |
| Aβ42, Aβ38, Aβ42/Aβ40 | 0.858 | 0.026 |
| Aβ42, Aβ42/Aβ40, Aβ42/Aβ38, AGE | 0.876 | 0.044 |
| Aβ42, Aβ40, Aβ42/Aβ38, Aβ40/Aβ38, AGE, PTEDUCAT, APOE4 | 0.872 | 0.041 |
| Aβ40, Aβ38, Aβ42/Aβ40, Aβ40/Aβ38, AGE, PTEDUCAT, PTGENDER | 0.858 | 0.027 |
| Aβ42, Aβ40, Aβ42/Aβ40, Aβ42/Aβ38, Aβ40/Aβ38, AGE, PTEDUCAT, PTGENDER, APOE4 | 0.864 | 0.032 |
| Aβ40, Aβ38, Aβ42/Aβ38, Aβ40/Aβ38, AGE, APOE4 | 0.857 | 0.026 |
| Aβ42, Aβ38, Aβ42/Aβ40, Aβ42/Aβ38, AGE | 0.874 | 0.043 |
| Aβ42, Aβ38, Aβ42/Aβ40, Aβ42/Aβ38, Aβ40/Aβ38 | 0.863 | 0.033 |
| Aβ42, Aβ40, Aβ42/Aβ40, Aβ40/Aβ38, AGE | 0.874 | 0.043 |
| Aβ40, Aβ38, Aβ42/Aβ40, Aβ42/Aβ38, Aβ40/Aβ38, APOE4 | 0.870 | 0.039 |
| Aβ40, Aβ42/Aβ40, Aβ40/Aβ38, AGE, PTEDUCAT, APOE4 | 0.863 | 0.032 |
| Aβ42, Aβ40, Aβ38, Aβ42/Aβ38, Aβ40/Aβ38, AGE, PTEDUCAT, PTGENDER | 0.854 | 0.023 |
| Aβ42, Aβ40, Aβ38, Aβ42/Aβ38, Aβ40/Aβ38, AGE, PTEDUCAT, APOE4 | 0.869 | 0.038 |
| Aβ40, Aβ38, Aβ42/Aβ40, AGE | 0.865 | 0.034 |
| Aβ38, Aβ42/Aβ40, Aβ42/Aβ38, PTEDUCAT, PTGENDER | 0.871 | 0.041 |
| Aβ40, Aβ38, Aβ42/Aβ40, Aβ40/Aβ38, PTEDUCAT, PTGENDER | 0.868 | 0.037 |
| Aβ42, Aβ42/Aβ40, AGE, PTGENDER, APOE4 | 0.866 | 0.036 |
| Aβ42, Aβ40, Aβ38, Aβ42/Aβ40, Aβ40/Aβ38, PTGENDER, APOE4 | 0.862 | 0.032 |
| Aβ40, Aβ42/Aβ40, Aβ42/Aβ38, Aβ40/Aβ38, PTEDUCAT, PTGENDER | 0.865 | 0.035 |
| Aβ40, Aβ38, Aβ42/Aβ40, Aβ40/Aβ38, APOE4 | 0.856 | 0.026 |
| Aβ42, Aβ40, Aβ38, Aβ42/Aβ40, Aβ42/Aβ38, Aβ40/Aβ38, AGE, PTGENDER, APOE4 | 0.868 | 0.038 |
| Aβ38, Aβ42/Aβ38, PTEDUCAT | 0.869 | 0.038 |
| Aβ42, Aβ40, Aβ38, Aβ42/Aβ38, Aβ40/Aβ38, PTEDUCAT, PTGENDER | 0.853 | 0.023 |
| Aβ42, Aβ42/Aβ40, Aβ42/Aβ38, APOE4 | 0.870 | 0.040 |
| Aβ40, Aβ42/Aβ40, Aβ42/Aβ38, AGE, PTEDUCAT | 0.876 | 0.046 |
| Aβ42, Aβ38, Aβ42/Aβ40, Aβ40/Aβ38 | 0.858 | 0.028 |
| Aβ40, Aβ42/Aβ38, APOE4 | 0.868 | 0.038 |
| Aβ42, Aβ40, Aβ42/Aβ38, Aβ40/Aβ38, AGE | 0.859 | 0.029 |
| Aβ40, Aβ42/Aβ38, AGE, PTEDUCAT, APOE4 | 0.873 | 0.043 |
| Aβ40, Aβ38, Aβ42/Aβ38, Aβ40/Aβ38, PTEDUCAT, PTGENDER | 0.853 | 0.023 |
| Aβ42, Aβ40, Aβ38, Aβ42/Aβ38, PTEDUCAT, PTGENDER | 0.857 | 0.028 |
| Aβ40, Aβ42/Aβ40, Aβ42/Aβ38, Aβ40/Aβ38, PTEDUCAT | 0.868 | 0.038 |
| Aβ40, Aβ38, Aβ42/Aβ40, Aβ42/Aβ38, Aβ40/Aβ38, PTGENDER | 0.868 | 0.039 |
| Aβ40, Aβ42/Aβ40, Aβ42/Aβ38, Aβ40/Aβ38, PTEDUCAT, APOE4 | 0.864 | 0.035 |
| Aβ42, Aβ38, Aβ42/Aβ40, Aβ42/Aβ38, Aβ40/Aβ38, APOE4 | 0.860 | 0.031 |
| Aβ42, Aβ42/Aβ38, PTEDUCAT, PTGENDER | 0.861 | 0.032 |
| Aβ40, Aβ38, Aβ42/Aβ40, Aβ42/Aβ38, Aβ40/Aβ38, AGE, APOE4 | 0.869 | 0.040 |
| Aβ42, Aβ40, Aβ42/Aβ38, Aβ40/Aβ38, PTGENDER | 0.868 | 0.039 |
| Aβ42, Aβ42/Aβ38, Aβ40/Aβ38, PTEDUCAT, PTGENDER | 0.870 | 0.041 |
| Aβ40, Aβ38, Aβ42/Aβ40, Aβ42/Aβ38, AGE, APOE4 | 0.849 | 0.020 |
| Aβ42, Aβ40, Aβ38, Aβ42/Aβ40, Aβ42/Aβ38, AGE, PTGENDER | 0.869 | 0.040 |
| Aβ42, Aβ40, Aβ42/Aβ38, Aβ40/Aβ38, PTEDUCAT, PTGENDER | 0.857 | 0.028 |
| Aβ42, Aβ38, Aβ42/Aβ40, AGE, PTEDUCAT, PTGENDER, APOE4 | 0.871 | 0.042 |
| Aβ40, Aβ42/Aβ38, Aβ40/Aβ38, AGE, PTEDUCAT, PTGENDER | 0.864 | 0.035 |
| Aβ40, Aβ42/Aβ40, Aβ40/Aβ38, PTEDUCAT, APOE4 | 0.868 | 0.040 |
| Aβ42, Aβ40, Aβ38, Aβ42/Aβ40, Aβ42/Aβ38, AGE, PTEDUCAT, PTGENDER, APOE4 | 0.872 | 0.043 |
| Aβ42, Aβ42/Aβ40, Aβ40/Aβ38, PTGENDER | 0.872 | 0.043 |
| Aβ38, Aβ42/Aβ40 | 0.870 | 0.041 |
| Aβ38, Aβ42/Aβ38, Aβ40/Aβ38, PTEDUCAT | 0.866 | 0.037 |
| Aβ40, Aβ38, Aβ42/Aβ38, Aβ40/Aβ38, AGE, PTGENDER | 0.876 | 0.048 |
| Aβ40, Aβ38, Aβ42/Aβ40, Aβ40/Aβ38, AGE, APOE4 | 0.855 | 0.027 |
| Aβ42, Aβ42/Aβ40, Aβ42/Aβ38, AGE, APOE4 | 0.872 | 0.044 |
| Aβ40, Aβ42/Aβ38, Aβ40/Aβ38, PTGENDER, APOE4 | 0.863 | 0.035 |
| Aβ40, Aβ42/Aβ38, Aβ40/Aβ38, PTEDUCAT, PTGENDER | 0.864 | 0.036 |
| Aβ40, Aβ42/Aβ40, Aβ40/Aβ38, AGE, PTEDUCAT, PTGENDER | 0.869 | 0.041 |
| Aβ40, Aβ38, Aβ42/Aβ38, PTEDUCAT, PTGENDER | 0.860 | 0.031 |
| Aβ42, Aβ42/Aβ40, Aβ40/Aβ38, AGE, APOE4 | 0.875 | 0.046 |
| Aβ42, Aβ42/Aβ40, Aβ42/Aβ38, Aβ40/Aβ38, AGE, PTGENDER | 0.864 | 0.035 |
| Aβ42, Aβ40, Aβ38, Aβ42/Aβ38, Aβ40/Aβ38, APOE4 | 0.870 | 0.042 |
| Aβ40, Aβ38, Aβ42/Aβ40, Aβ40/Aβ38, PTEDUCAT, PTGENDER, APOE4 | 0.865 | 0.037 |
| Aβ40, Aβ38, Aβ42/Aβ38, AGE | 0.869 | 0.041 |
| Aβ40, Aβ42/Aβ40, Aβ42/Aβ38, PTEDUCAT | 0.871 | 0.043 |
| Aβ42, Aβ42/Aβ40, PTGENDER, APOE4 | 0.872 | 0.044 |
| Aβ42, Aβ40, Aβ38, Aβ42/Aβ38, Aβ40/Aβ38, PTEDUCAT, APOE4 | 0.865 | 0.037 |
| Aβ40, Aβ38, Aβ42/Aβ40, AGE, PTEDUCAT, PTGENDER | 0.863 | 0.035 |
| Aβ42, Aβ40, AGE, PTEDUCAT | 0.871 | 0.043 |
| Aβ42, Aβ40, Aβ38, Aβ42/Aβ40, Aβ42/Aβ38, APOE4 | 0.870 | 0.042 |
| Aβ42, Aβ40, Aβ38, Aβ42/Aβ38, AGE | 0.867 | 0.039 |
| Aβ42, Aβ40, Aβ38, Aβ42/Aβ40, Aβ42/Aβ38, AGE, APOE4 | 0.869 | 0.041 |
| Aβ38, Aβ42/Aβ40, Aβ40/Aβ38, AGE | 0.861 | 0.033 |
| Aβ42, Aβ40, Aβ42/Aβ40, APOE4 | 0.866 | 0.038 |
| Aβ42, Aβ40, Aβ38, Aβ40/Aβ38 | 0.868 | 0.041 |
| Aβ42, Aβ42/Aβ40, PTGENDER | 0.873 | 0.045 |
| Aβ40, Aβ38, Aβ42/Aβ40, APOE4 | 0.854 | 0.027 |
| Aβ42, Aβ40, Aβ38, Aβ42/Aβ38, Aβ40/Aβ38, AGE, PTGENDER | 0.859 | 0.031 |
| Aβ42, Aβ40, Aβ38, Aβ42/Aβ40, Aβ42/Aβ38, PTGENDER | 0.869 | 0.042 |
| Aβ42, Aβ40, Aβ42/Aβ40, AGE, PTEDUCAT, PTGENDER | 0.874 | 0.046 |
| Aβ40, Aβ38, Aβ42/Aβ38, AGE, PTEDUCAT, APOE4 | 0.864 | 0.036 |
| Aβ40, Aβ38, Aβ42/Aβ40, AGE, APOE4 | 0.854 | 0.026 |
| Aβ40, Aβ38, Aβ42/Aβ40, PTEDUCAT, PTGENDER, APOE4 | 0.864 | 0.037 |
| Aβ42, Aβ42/Aβ40, Aβ40/Aβ38, AGE | 0.875 | 0.048 |
| Aβ42, Aβ40, Aβ38, Aβ42/Aβ40, AGE, PTGENDER | 0.870 | 0.043 |
| Aβ42, Aβ38, Aβ42/Aβ40, Aβ40/Aβ38, AGE | 0.854 | 0.026 |
| Aβ40, Aβ38, Aβ42/Aβ40, AGE, PTEDUCAT, PTGENDER, APOE4 | 0.865 | 0.038 |
| Aβ42, Aβ40, Aβ38, Aβ42/Aβ40, Aβ42/Aβ38, Aβ40/Aβ38, PTGENDER, APOE4 | 0.868 | 0.041 |
| Aβ38, Aβ42/Aβ38, Aβ40/Aβ38, AGE, PTEDUCAT, PTGENDER | 0.869 | 0.042 |
| Aβ42, Aβ40, Aβ38, Aβ42/Aβ40, AGE, APOE4 | 0.848 | 0.021 |
| Aβ42, Aβ42/Aβ40, Aβ40/Aβ38, PTGENDER, APOE4 | 0.871 | 0.044 |
| Aβ42, Aβ40, Aβ38, Aβ42/Aβ40, Aβ40/Aβ38, AGE, APOE4 | 0.861 | 0.034 |
| Aβ42, Aβ42/Aβ40, Aβ42/Aβ38, Aβ40/Aβ38, PTGENDER, APOE4 | 0.862 | 0.035 |
| Aβ40, Aβ42/Aβ40, Aβ40/Aβ38, PTEDUCAT, PTGENDER | 0.869 | 0.042 |
| Aβ42, Aβ38, Aβ42/Aβ38, Aβ40/Aβ38 | 0.867 | 0.040 |
| Aβ42, Aβ40, Aβ42/Aβ38, PTEDUCAT, APOE4 | 0.872 | 0.045 |
| Aβ40, Aβ38, Aβ42/Aβ38, Aβ40/Aβ38, AGE, PTEDUCAT, PTGENDER, APOE4 | 0.858 | 0.031 |
| Aβ40, Aβ38, Aβ42/Aβ40, Aβ40/Aβ38, PTGENDER | 0.854 | 0.027 |
| Aβ42, Aβ38, Aβ42/Aβ38, AGE, PTEDUCAT | 0.859 | 0.032 |
| Aβ40, Aβ38, Aβ42/Aβ38, PTEDUCAT, APOE4 | 0.864 | 0.037 |
| Aβ42, Aβ40, Aβ38, Aβ42/Aβ40, PTGENDER | 0.868 | 0.042 |
| Aβ42, Aβ40, Aβ38, Aβ42/Aβ38, AGE, PTEDUCAT, PTGENDER | 0.858 | 0.032 |
| Aβ42, Aβ40, Aβ42/Aβ40, Aβ40/Aβ38, PTGENDER | 0.870 | 0.044 |
| Aβ40, Aβ42/Aβ40, Aβ42/Aβ38, AGE | 0.872 | 0.045 |
| Aβ42, Aβ40, Aβ42/Aβ40, Aβ40/Aβ38, AGE, PTEDUCAT, PTGENDER, APOE4 | 0.871 | 0.045 |
| Aβ42, Aβ40, Aβ42/Aβ40, Aβ42/Aβ38, Aβ40/Aβ38 | 0.861 | 0.035 |
| Aβ38, Aβ42/Aβ38, AGE, PTEDUCAT | 0.867 | 0.041 |
| Aβ38, Aβ42/Aβ40, APOE4 | 0.870 | 0.044 |
| Aβ42, Aβ42/Aβ40, Aβ40/Aβ38, AGE, PTGENDER | 0.872 | 0.046 |
| Aβ42, Aβ40, Aβ42/Aβ38, Aβ40/Aβ38, PTGENDER, APOE4 | 0.861 | 0.035 |
| Aβ40, Aβ38, Aβ42/Aβ40, Aβ42/Aβ38, Aβ40/Aβ38, PTEDUCAT, PTGENDER, APOE4 | 0.869 | 0.044 |
| Aβ42, Aβ40, AGE, APOE4 | 0.854 | 0.029 |
| Aβ40, Aβ42/Aβ38, PTEDUCAT, PTGENDER | 0.868 | 0.043 |
| Aβ42, Aβ40, Aβ42/Aβ38, Aβ40/Aβ38, AGE, PTEDUCAT, PTGENDER | 0.864 | 0.039 |
| Aβ42, Aβ40, Aβ38, Aβ42/Aβ38, Aβ40/Aβ38, PTGENDER | 0.857 | 0.032 |
| Aβ42, Aβ40, Aβ38, Aβ42/Aβ38, Aβ40/Aβ38, AGE, APOE4 | 0.854 | 0.029 |
| Aβ40, Aβ42/Aβ40, Aβ42/Aβ38, Aβ40/Aβ38, AGE, APOE4 | 0.864 | 0.038 |
| Aβ42, Aβ42/Aβ40, Aβ42/Aβ38, AGE, PTGENDER | 0.868 | 0.042 |
| Aβ42, Aβ42/Aβ40, Aβ42/Aβ38, PTGENDER, APOE4 | 0.868 | 0.042 |
| Aβ40, Aβ38, Aβ42/Aβ40, Aβ42/Aβ38, AGE, PTEDUCAT, APOE4 | 0.873 | 0.048 |
| Aβ38, Aβ42/Aβ40, Aβ42/Aβ38, AGE, PTEDUCAT | 0.871 | 0.045 |
| Aβ42, Aβ40, Aβ42/Aβ38, PTEDUCAT, PTGENDER | 0.870 | 0.044 |
| Aβ40, Aβ42/Aβ38, Aβ40/Aβ38, AGE, APOE4 | 0.871 | 0.045 |
| Aβ42, Aβ40, Aβ38, Aβ42/Aβ38, PTGENDER | 0.869 | 0.044 |
| Aβ42, Aβ40, Aβ38, Aβ40/Aβ38, AGE | 0.869 | 0.043 |
| Aβ40, Aβ42/Aβ38, Aβ40/Aβ38, PTGENDER | 0.861 | 0.036 |
| Aβ40, Aβ42/Aβ40, Aβ42/Aβ38, Aβ40/Aβ38 | 0.875 | 0.050 |
| Aβ40, Aβ42/Aβ40, AGE, PTEDUCAT, APOE4 | 0.868 | 0.043 |
| Aβ42, Aβ42/Aβ40, Aβ42/Aβ38, Aβ40/Aβ38, PTGENDER | 0.862 | 0.037 |
| Aβ42, Aβ38, Aβ42/Aβ38, Aβ40/Aβ38, APOE4 | 0.881 | 0.056 |
| Aβ42, Aβ38, Aβ42/Aβ40, AGE | 0.860 | 0.035 |
| Aβ42, Aβ38, Aβ42/Aβ38, Aβ40/Aβ38, PTEDUCAT | 0.854 | 0.029 |
| Aβ42, Aβ38, Aβ42/Aβ40, Aβ42/Aβ38, Aβ40/Aβ38, PTGENDER | 0.859 | 0.034 |
| Aβ42, Aβ42/Aβ38, PTEDUCAT, PTGENDER, APOE4 | 0.852 | 0.027 |
| Aβ40, Aβ42/Aβ38, AGE, APOE4 | 0.856 | 0.031 |
| Aβ42, Aβ42/Aβ38, Aβ40/Aβ38 | 0.844 | 0.019 |
| Aβ40, Aβ42/Aβ40, Aβ42/Aβ38, Aβ40/Aβ38, PTGENDER, APOE4 | 0.868 | 0.043 |
| Aβ42, Aβ40, Aβ38, Aβ42/Aβ40, Aβ42/Aβ38, PTEDUCAT, PTGENDER, APOE4 | 0.870 | 0.045 |
| Aβ42, Aβ40, AGE, PTEDUCAT, APOE4 | 0.868 | 0.043 |
| Aβ42, Aβ42/Aβ38, Aβ40/Aβ38, AGE, PTEDUCAT, PTGENDER, APOE4 | 0.869 | 0.045 |
| Aβ42, Aβ40, Aβ42/Aβ40, Aβ42/Aβ38, AGE, PTEDUCAT, PTGENDER, APOE4 | 0.868 | 0.043 |
| Aβ40, Aβ38, Aβ42/Aβ40, Aβ42/Aβ38, APOE4 | 0.856 | 0.031 |
| Aβ38, Aβ42/Aβ40, Aβ40/Aβ38, PTGENDER, APOE4 | 0.870 | 0.045 |
| Aβ42, Aβ38, Aβ42/Aβ40, APOE4 | 0.868 | 0.044 |
| Aβ42, Aβ38, Aβ42/Aβ38, PTEDUCAT | 0.863 | 0.038 |
| Aβ42, Aβ38, Aβ42/Aβ40, Aβ42/Aβ38 | 0.868 | 0.043 |
| Aβ42, Aβ42/Aβ40, Aβ42/Aβ38, PTGENDER | 0.870 | 0.046 |
| Aβ40, Aβ42/Aβ38, Aβ40/Aβ38, PTEDUCAT, PTGENDER, APOE4 | 0.869 | 0.045 |
| Aβ42, Aβ40, Aβ42/Aβ40, AGE, PTEDUCAT, PTGENDER, APOE4 | 0.871 | 0.047 |
| Aβ38, Aβ42/Aβ40, AGE, PTEDUCAT, APOE4 | 0.873 | 0.049 |
| Aβ42, Aβ38, Aβ40/Aβ38 | 0.857 | 0.033 |
| Aβ40, Aβ42/Aβ40, AGE | 0.870 | 0.046 |
| Aβ38, Aβ42/Aβ38, Aβ40/Aβ38, AGE, PTGENDER | 0.871 | 0.047 |
| Aβ38, Aβ42/Aβ40, Aβ40/Aβ38, PTEDUCAT, PTGENDER | 0.858 | 0.034 |
| Aβ42, Aβ38, Aβ40/Aβ38, AGE | 0.851 | 0.027 |
| Aβ42, Aβ40, Aβ38, Aβ42/Aβ40, AGE, PTEDUCAT, APOE4 | 0.869 | 0.045 |
| Aβ42, Aβ38, Aβ42/Aβ40, Aβ40/Aβ38, PTGENDER, APOE4 | 0.867 | 0.043 |
| Aβ40, Aβ38, Aβ42/Aβ38, Aβ40/Aβ38, APOE4 | 0.852 | 0.028 |
| Aβ40, Aβ38, Aβ42/Aβ40, Aβ40/Aβ38, AGE, PTGENDER | 0.850 | 0.026 |
| Aβ40, Aβ38, Aβ42/Aβ40, Aβ40/Aβ38, AGE, PTEDUCAT, PTGENDER, APOE4 | 0.856 | 0.033 |
| Aβ38, Aβ42/Aβ38, Aβ40/Aβ38, AGE, PTEDUCAT | 0.856 | 0.033 |
| Aβ38, Aβ42/Aβ38, Aβ40/Aβ38, PTEDUCAT, PTGENDER, APOE4 | 0.866 | 0.042 |
| Aβ38, Aβ42/Aβ38, Aβ40/Aβ38, PTEDUCAT, APOE4 | 0.853 | 0.030 |
| Aβ40, Aβ38, Aβ42/Aβ38, AGE, APOE4 | 0.849 | 0.026 |
| Aβ42, Aβ40, Aβ42/Aβ40, Aβ42/Aβ38, AGE, APOE4 | 0.855 | 0.032 |
| Aβ40, Aβ42/Aβ40, Aβ42/Aβ38, Aβ40/Aβ38, AGE, PTEDUCAT, PTGENDER | 0.860 | 0.037 |
| Aβ42, Aβ42/Aβ40, Aβ40/Aβ38, AGE, PTGENDER, APOE4 | 0.869 | 0.046 |
| Aβ42, Aβ40, Aβ42/Aβ40, AGE, PTGENDER, APOE4 | 0.868 | 0.045 |
| Aβ42, Aβ40, Aβ42/Aβ40, AGE, APOE4 | 0.857 | 0.034 |
| Aβ42, Aβ42/Aβ38, AGE, PTEDUCAT | 0.866 | 0.043 |
| Aβ40, Aβ38, Aβ42/Aβ38, AGE, PTEDUCAT, PTGENDER, APOE4 | 0.861 | 0.039 |
| Aβ42, Aβ38, Aβ40/Aβ38, PTEDUCAT, PTGENDER | 0.858 | 0.035 |
| Aβ42, Aβ38, Aβ40/Aβ38, PTEDUCAT, PTGENDER, APOE4 | 0.858 | 0.035 |
| Aβ40, Aβ42/Aβ38, AGE, PTEDUCAT, PTGENDER | 0.871 | 0.048 |
| Aβ42, Aβ42/Aβ40, Aβ42/Aβ38, Aβ40/Aβ38, AGE, PTGENDER, APOE4 | 0.857 | 0.034 |
| Aβ42, Aβ40, Aβ38, Aβ40/Aβ38, AGE, PTEDUCAT, PTGENDER | 0.866 | 0.043 |
| Aβ42, Aβ40, Aβ42/Aβ40, PTEDUCAT, PTGENDER, APOE4 | 0.869 | 0.047 |
| Aβ42, Aβ40, Aβ42/Aβ40, Aβ42/Aβ38, Aβ40/Aβ38, PTGENDER, APOE4 | 0.859 | 0.036 |
| Aβ42, Aβ40, Aβ38, Aβ42/Aβ40, APOE4 | 0.866 | 0.044 |
| Aβ38, Aβ42/Aβ40, AGE, PTEDUCAT | 0.871 | 0.049 |
| Aβ42, Aβ40, Aβ42/Aβ38, PTEDUCAT, PTGENDER, APOE4 | 0.853 | 0.031 |
| Aβ42, Aβ38, Aβ42/Aβ40, Aβ42/Aβ38, AGE, PTEDUCAT, APOE4 | 0.868 | 0.046 |
| Aβ40, Aβ38, Aβ42/Aβ38, PTEDUCAT, PTGENDER, APOE4 | 0.854 | 0.031 |
| Aβ40, Aβ38, Aβ42/Aβ38, Aβ40/Aβ38, PTGENDER | 0.845 | 0.023 |
| Aβ42, Aβ38, Aβ42/Aβ40, Aβ40/Aβ38, AGE, APOE4 | 0.857 | 0.035 |
| Aβ42, Aβ40, Aβ38, Aβ42/Aβ40, Aβ40/Aβ38, AGE, PTEDUCAT, PTGENDER, APOE4 | 0.859 | 0.037 |
| Aβ42, Aβ40, Aβ38, Aβ40/Aβ38, PTEDUCAT, APOE4 | 0.857 | 0.035 |
| Aβ42, Aβ38, Aβ42/Aβ40, Aβ40/Aβ38, APOE4 | 0.863 | 0.041 |
| Aβ42, Aβ40, Aβ42/Aβ38, AGE, PTGENDER | 0.865 | 0.043 |
| Aβ42, Aβ40, Aβ38, Aβ40/Aβ38, AGE, PTGENDER | 0.868 | 0.046 |
| Aβ42, Aβ40, Aβ38, Aβ42/Aβ38, PTEDUCAT, APOE4 | 0.861 | 0.039 |
| Aβ42, Aβ42/Aβ38, Aβ40/Aβ38, PTEDUCAT, PTGENDER, APOE4 | 0.868 | 0.046 |
| Aβ42, Aβ38, Aβ42/Aβ38, AGE | 0.859 | 0.037 |
| Aβ42, Aβ38, Aβ42/Aβ40, Aβ42/Aβ38, Aβ40/Aβ38, PTGENDER, APOE4 | 0.855 | 0.033 |
| Aβ40, Aβ38, Aβ42/Aβ40, AGE, PTGENDER | 0.860 | 0.038 |
| Aβ38, Aβ42/Aβ40, Aβ40/Aβ38, AGE, PTEDUCAT, PTGENDER | 0.860 | 0.038 |
| Aβ42, Aβ40, Aβ38, Aβ42/Aβ38, APOE4 | 0.856 | 0.034 |
| Aβ42, Aβ40, Aβ38, PTEDUCAT, APOE4 | 0.860 | 0.038 |
| Aβ42, Aβ38, Aβ42/Aβ40, Aβ40/Aβ38, PTGENDER | 0.867 | 0.045 |
| Aβ38, Aβ42/Aβ40, Aβ40/Aβ38, PTEDUCAT, APOE4 | 0.867 | 0.046 |
| Aβ42, Aβ40, Aβ42/Aβ38, AGE, APOE4 | 0.856 | 0.034 |
| Aβ42, Aβ40, Aβ38, Aβ42/Aβ38, Aβ40/Aβ38, AGE, PTEDUCAT, PTGENDER, APOE4 | 0.852 | 0.030 |
| Aβ40, Aβ38, Aβ42/Aβ40, Aβ42/Aβ38, Aβ40/Aβ38, AGE, PTGENDER, APOE4 | 0.865 | 0.044 |
| Aβ42, Aβ38, Aβ42/Aβ40, Aβ42/Aβ38, Aβ40/Aβ38, AGE, PTGENDER | 0.856 | 0.034 |
| Aβ38, Aβ42/Aβ40, Aβ42/Aβ38, Aβ40/Aβ38 | 0.871 | 0.049 |
| Aβ38, Aβ42/Aβ40, Aβ42/Aβ38, PTEDUCAT, APOE4 | 0.867 | 0.046 |
| Aβ42, Aβ38, Aβ42/Aβ38, Aβ40/Aβ38, AGE | 0.848 | 0.027 |
| Aβ38, Aβ42/Aβ40, Aβ42/Aβ38, AGE, PTEDUCAT, PTGENDER | 0.865 | 0.044 |
| Aβ42, Aβ40, Aβ38, Aβ42/Aβ38, AGE, PTEDUCAT, APOE4 | 0.867 | 0.046 |
| Aβ38, Aβ42/Aβ40, Aβ42/Aβ38, Aβ40/Aβ38, AGE, APOE4 | 0.862 | 0.040 |
| Aβ40, Aβ42/Aβ40, AGE, APOE4 | 0.858 | 0.037 |
| Aβ42, Aβ40, Aβ38, Aβ42/Aβ40, AGE, PTEDUCAT, PTGENDER, APOE4 | 0.851 | 0.030 |
| Aβ42, Aβ40, Aβ42/Aβ40, Aβ42/Aβ38, AGE, PTGENDER | 0.869 | 0.048 |
| Aβ40, Aβ42/Aβ40, PTEDUCAT, PTGENDER | 0.867 | 0.047 |
| Aβ42, Aβ42/Aβ40, Aβ42/Aβ38, AGE, PTGENDER, APOE4 | 0.861 | 0.040 |
| Aβ38, Aβ42/Aβ38, Aβ40/Aβ38, AGE | 0.869 | 0.049 |
| Aβ42, Aβ40, Aβ42/Aβ40, Aβ42/Aβ38, PTEDUCAT, PTGENDER, APOE4 | 0.867 | 0.046 |
| Aβ40, Aβ38, Aβ42/Aβ40, Aβ42/Aβ38, PTGENDER | 0.871 | 0.050 |
| Aβ42, Aβ38, Aβ42/Aβ40, AGE, PTGENDER | 0.868 | 0.048 |
| Aβ42, Aβ40, Aβ38, Aβ42/Aβ38, Aβ40/Aβ38, AGE, PTGENDER, APOE4 | 0.873 | 0.052 |
| Aβ42, Aβ40, Aβ42/Aβ38, Aβ40/Aβ38, AGE, PTEDUCAT, PTGENDER, APOE4 | 0.869 | 0.048 |
| Aβ42, Aβ40, Aβ40/Aβ38, AGE | 0.858 | 0.038 |
| Aβ40, Aβ38, Aβ42/Aβ38, Aβ40/Aβ38, PTEDUCAT, PTGENDER, APOE4 | 0.854 | 0.034 |
| Aβ42, Aβ38, Aβ42/Aβ40, Aβ42/Aβ38, Aβ40/Aβ38, AGE, APOE4 | 0.857 | 0.037 |
| Aβ42, Aβ38, Aβ42/Aβ40, Aβ40/Aβ38, AGE, PTGENDER | 0.866 | 0.046 |
| Aβ38, Aβ42/Aβ40, PTEDUCAT, APOE4 | 0.871 | 0.051 |
| Aβ40, Aβ42/Aβ40, Aβ40/Aβ38, PTEDUCAT, PTGENDER, APOE4 | 0.863 | 0.043 |
| Aβ42, Aβ38, Aβ42/Aβ38 | 0.860 | 0.040 |
| Aβ42, Aβ42/Aβ38, AGE | 0.855 | 0.035 |
| Aβ42, Aβ38, Aβ42/Aβ40, Aβ42/Aβ38, AGE, PTGENDER, APOE4 | 0.859 | 0.040 |
| Aβ40, Aβ38, Aβ42/Aβ40, Aβ42/Aβ38, Aβ40/Aβ38, PTGENDER, APOE4 | 0.866 | 0.046 |
| Aβ40, Aβ38, Aβ42/Aβ38, Aβ40/Aβ38, AGE, PTGENDER, APOE4 | 0.868 | 0.048 |
| Aβ42, Aβ38, Aβ42/Aβ40, AGE, APOE4 | 0.862 | 0.042 |
| Aβ42, Aβ40, Aβ38, AGE, PTEDUCAT, PTGENDER | 0.860 | 0.041 |
| Aβ40, Aβ38, Aβ42/Aβ40, Aβ40/Aβ38, AGE, PTGENDER, APOE4 | 0.869 | 0.049 |
| Aβ42, Aβ40, Aβ42/Aβ38, APOE4 | 0.857 | 0.038 |
| Aβ42, Aβ38, Aβ42/Aβ40, PTGENDER, APOE4 | 0.867 | 0.047 |
| Aβ42, Aβ40, Aβ38, Aβ42/Aβ40, Aβ42/Aβ38, AGE, PTGENDER, APOE4 | 0.866 | 0.047 |
| Aβ42, Aβ38, Aβ42/Aβ40, Aβ42/Aβ38, PTGENDER | 0.866 | 0.047 |
| Aβ42, Aβ42/Aβ38, PTEDUCAT, APOE4 | 0.863 | 0.043 |
| Aβ42, Aβ40, Aβ40/Aβ38, APOE4 | 0.852 | 0.033 |
| Aβ42, Aβ40, Aβ42/Aβ40, Aβ40/Aβ38, AGE, PTGENDER | 0.868 | 0.048 |
| Aβ42, Aβ40, Aβ40/Aβ38, PTEDUCAT, APOE4 | 0.857 | 0.037 |
| Aβ42, Aβ42/Aβ38, Aβ40/Aβ38, AGE, PTGENDER, APOE4 | 0.847 | 0.028 |
| Aβ42, Aβ38, Aβ40/Aβ38, AGE, PTEDUCAT, PTGENDER, APOE4 | 0.857 | 0.038 |
| Aβ42, Aβ38, Aβ42/Aβ40, PTGENDER | 0.867 | 0.047 |
| Aβ38, Aβ42/Aβ40, Aβ42/Aβ38, PTEDUCAT, PTGENDER, APOE4 | 0.857 | 0.037 |
| Aβ42, Aβ40, Aβ38, Aβ40/Aβ38, AGE, PTEDUCAT, APOE4 | 0.862 | 0.042 |
| Aβ40, Aβ38, Aβ42/Aβ40, Aβ40/Aβ38, PTGENDER, APOE4 | 0.869 | 0.050 |
| Aβ42, Aβ38, Aβ42/Aβ38, Aβ40/Aβ38, PTEDUCAT, APOE4 | 0.871 | 0.052 |
| Aβ38, Aβ42/Aβ40, Aβ42/Aβ38, AGE, PTEDUCAT, PTGENDER, APOE4 | 0.853 | 0.034 |
| Aβ42, Aβ40, Aβ40/Aβ38, PTEDUCAT | 0.858 | 0.039 |
| Aβ38, Aβ42/Aβ38, Aβ40/Aβ38, PTGENDER | 0.856 | 0.037 |
| Aβ42, Aβ40, Aβ38, Aβ42/Aβ38, AGE, PTGENDER | 0.868 | 0.049 |
| Aβ40, Aβ42/Aβ40, Aβ42/Aβ38, Aβ40/Aβ38, AGE | 0.862 | 0.044 |
| Aβ38, Aβ42/Aβ40, PTEDUCAT, PTGENDER | 0.870 | 0.051 |
| Aβ40, Aβ38, Aβ42/Aβ40, AGE, PTGENDER, APOE4 | 0.862 | 0.044 |
| Aβ38, Aβ42/Aβ38 | 0.858 | 0.039 |
| Aβ42, Aβ42/Aβ38, Aβ40/Aβ38, APOE4 | 0.864 | 0.045 |
| Aβ40, Aβ42/Aβ40, AGE, PTEDUCAT, PTGENDER | 0.871 | 0.052 |
| Aβ42, Aβ40, PTEDUCAT, APOE4 | 0.852 | 0.034 |
| Aβ40, Aβ38, Aβ42/Aβ40, Aβ42/Aβ38, PTEDUCAT, PTGENDER, APOE4 | 0.868 | 0.050 |
| Aβ38, Aβ42/Aβ38, PTEDUCAT, PTGENDER | 0.856 | 0.038 |
| Aβ40, Aβ38, Aβ42/Aβ38, PTGENDER | 0.855 | 0.037 |
| Aβ42, Aβ40, Aβ42/Aβ38, Aβ40/Aβ38, PTEDUCAT, PTGENDER, APOE4 | 0.838 | 0.020 |
| Aβ42, Aβ38, Aβ42/Aβ40, Aβ42/Aβ38, Aβ40/Aβ38, AGE, PTEDUCAT, PTGENDER, APOE4 | 0.856 | 0.038 |
| Aβ38, Aβ42/Aβ38, Aβ40/Aβ38, APOE4 | 0.862 | 0.044 |
| Aβ42, Aβ42/Aβ38, Aβ40/Aβ38, PTGENDER | 0.865 | 0.047 |
| Aβ42, Aβ40, Aβ38, Aβ42/Aβ40, Aβ42/Aβ38, PTGENDER, APOE4 | 0.866 | 0.048 |
| Aβ38, Aβ42/Aβ40, Aβ40/Aβ38, AGE, PTEDUCAT, APOE4 | 0.863 | 0.045 |
| Aβ42, Aβ38, Aβ42/Aβ38, Aβ40/Aβ38, AGE, PTGENDER | 0.879 | 0.061 |
| Aβ38, Aβ42/Aβ40, Aβ42/Aβ38, Aβ40/Aβ38, PTEDUCAT, APOE4 | 0.858 | 0.041 |
| Aβ40, Aβ42/Aβ40, Aβ42/Aβ38, Aβ40/Aβ38, APOE4 | 0.853 | 0.035 |
| Aβ38, Aβ42/Aβ38, Aβ40/Aβ38, AGE, PTGENDER, APOE4 | 0.871 | 0.054 |
| Aβ42, Aβ40, Aβ38, Aβ42/Aβ40, PTEDUCAT, PTGENDER, APOE4 | 0.864 | 0.047 |
| Aβ38, Aβ42/Aβ40, Aβ42/Aβ38, AGE | 0.862 | 0.044 |
| Aβ40, Aβ38, Aβ42/Aβ40, Aβ42/Aβ38, AGE, PTEDUCAT, PTGENDER, APOE4 | 0.871 | 0.053 |
| Aβ40, Aβ38, Aβ42/Aβ38, AGE, PTGENDER | 0.855 | 0.038 |
| Aβ42, Aβ38, Aβ42/Aβ38, Aβ40/Aβ38, PTEDUCAT, PTGENDER, APOE4 | 0.875 | 0.057 |
| Aβ38, Aβ42/Aβ40, Aβ40/Aβ38, PTEDUCAT, PTGENDER, APOE4 | 0.861 | 0.044 |
| Aβ42, Aβ40, Aβ40/Aβ38 | 0.856 | 0.039 |
| Aβ42, Aβ40, Aβ42/Aβ40, Aβ42/Aβ38, APOE4 | 0.862 | 0.045 |
| Aβ42, Aβ40, Aβ42/Aβ38, AGE, PTEDUCAT, PTGENDER | 0.867 | 0.049 |
| Aβ42, Aβ40, Aβ38, Aβ40/Aβ38, PTEDUCAT | 0.856 | 0.039 |
| Aβ40, Aβ42/Aβ40, Aβ40/Aβ38, AGE, PTEDUCAT, PTGENDER, APOE4 | 0.861 | 0.044 |
| Aβ38, Aβ42/Aβ40, Aβ42/Aβ38, APOE4 | 0.854 | 0.037 |
| Aβ42, Aβ40, Aβ38, Aβ42/Aβ38, Aβ40/Aβ38, PTEDUCAT, PTGENDER, APOE4 | 0.852 | 0.035 |
| Aβ42, Aβ40, Aβ42/Aβ40, AGE, PTGENDER | 0.867 | 0.050 |
| Aβ42, Aβ38, Aβ42/Aβ40, AGE, PTGENDER, APOE4 | 0.865 | 0.048 |
| Aβ38, Aβ42/Aβ40, AGE, APOE4 | 0.849 | 0.032 |
| Aβ42, Aβ38, Aβ42/Aβ38, Aβ40/Aβ38, AGE, PTGENDER, APOE4 | 0.878 | 0.061 |
| Aβ42, Aβ40, Aβ42/Aβ40, Aβ40/Aβ38, AGE, APOE4 | 0.866 | 0.050 |
| Aβ40, Aβ42/Aβ40, Aβ42/Aβ38, AGE, PTEDUCAT, PTGENDER | 0.868 | 0.052 |
| Aβ38, Aβ42/Aβ40, AGE | 0.867 | 0.050 |
| Aβ42, Aβ40, Aβ40/Aβ38, AGE, PTEDUCAT | 0.860 | 0.043 |
| Aβ42, Aβ38, Aβ42/Aβ38, Aβ40/Aβ38, AGE, PTEDUCAT, PTGENDER | 0.876 | 0.060 |
| Aβ42, Aβ42/Aβ38, AGE, PTEDUCAT, PTGENDER | 0.863 | 0.047 |
| Aβ42, Aβ40, Aβ38, Aβ42/Aβ38, AGE, PTGENDER, APOE4 | 0.852 | 0.036 |
| Aβ40, Aβ42/Aβ38, AGE, PTGENDER, APOE4 | 0.858 | 0.042 |
| Aβ38, Aβ42/Aβ40, Aβ42/Aβ38, Aβ40/Aβ38, APOE4 | 0.865 | 0.049 |
| Aβ40, Aβ42/Aβ38, Aβ40/Aβ38, AGE, PTGENDER | 0.855 | 0.039 |
| Aβ40, Aβ42/Aβ38, Aβ40/Aβ38, AGE, PTGENDER, APOE4 | 0.866 | 0.050 |
| Aβ42, Aβ38, Aβ42/Aβ40, Aβ42/Aβ38, APOE4 | 0.860 | 0.044 |
| Aβ40, Aβ42/Aβ40, Aβ42/Aβ38, Aβ40/Aβ38, AGE, PTGENDER | 0.863 | 0.047 |
| Aβ38, Aβ42/Aβ40, Aβ42/Aβ38, AGE, PTEDUCAT, APOE4 | 0.864 | 0.048 |
| Aβ38, Aβ42/Aβ38, Aβ40/Aβ38, AGE, PTEDUCAT, PTGENDER, APOE4 | 0.867 | 0.051 |
| Aβ38, Aβ42/Aβ38, AGE | 0.855 | 0.040 |
| Aβ38, Aβ42/Aβ40, Aβ42/Aβ38, AGE, APOE4 | 0.865 | 0.050 |
| Aβ38, Aβ42/Aβ40, Aβ40/Aβ38, PTGENDER | 0.870 | 0.055 |
| Aβ42, Aβ38, Aβ42/Aβ40, Aβ42/Aβ38, AGE, APOE4 | 0.850 | 0.035 |
| Aβ42, Aβ42/Aβ38, Aβ40/Aβ38, AGE, PTGENDER | 0.848 | 0.033 |
| Aβ40, Aβ38, Aβ42/Aβ38, AGE, PTGENDER, APOE4 | 0.844 | 0.029 |
| Aβ40, Aβ42/Aβ40, Aβ42/Aβ38, AGE, PTEDUCAT, APOE4 | 0.867 | 0.052 |
| Aβ40, Aβ42/Aβ40, Aβ42/Aβ38 | 0.866 | 0.051 |
| Aβ40, Aβ42/Aβ40, Aβ42/Aβ38, APOE4 | 0.859 | 0.044 |
| Aβ42, Aβ40, Aβ38, Aβ42/Aβ38, AGE, APOE4 | 0.855 | 0.040 |
| Aβ38, Aβ42/Aβ40, Aβ40/Aβ38, AGE, PTGENDER | 0.869 | 0.054 |
| Aβ40, Aβ42/Aβ40, Aβ40/Aβ38, AGE, PTGENDER, APOE4 | 0.873 | 0.058 |
| Aβ42, Aβ40, Aβ38, PTEDUCAT, PTGENDER | 0.861 | 0.046 |
| Aβ42, Aβ40, Aβ38, AGE, APOE4 | 0.844 | 0.029 |
| Aβ42, Aβ42/Aβ38 | 0.857 | 0.042 |
| Aβ42, Aβ40, Aβ38, Aβ42/Aβ38, AGE, PTEDUCAT, PTGENDER, APOE4 | 0.855 | 0.041 |
| Aβ38, Aβ42/Aβ38, Aβ40/Aβ38, PTEDUCAT, PTGENDER | 0.853 | 0.039 |
| Aβ42, Aβ38, Aβ40/Aβ38, AGE, PTEDUCAT | 0.858 | 0.044 |
| Aβ40, Aβ38, Aβ42/Aβ38, Aβ40/Aβ38, PTGENDER, APOE4 | 0.847 | 0.033 |
| Aβ40, Aβ42/Aβ40, Aβ42/Aβ38, Aβ40/Aβ38, AGE, PTEDUCAT, APOE4 | 0.855 | 0.041 |
| Aβ42, Aβ40, Aβ38, APOE4 | 0.844 | 0.031 |
| Aβ42, Aβ40, Aβ42/Aβ38, AGE, PTEDUCAT, PTGENDER, APOE4 | 0.864 | 0.051 |
| Aβ42, Aβ40, Aβ38, Aβ40/Aβ38, PTEDUCAT, PTGENDER | 0.862 | 0.048 |
| Aβ42, Aβ38, Aβ42/Aβ38, Aβ40/Aβ38, AGE, PTEDUCAT, PTGENDER, APOE4 | 0.866 | 0.052 |
| Aβ42, Aβ38, APOE4 | 0.844 | 0.030 |
| Aβ42, Aβ40, Aβ40/Aβ38, AGE, PTEDUCAT, APOE4 | 0.850 | 0.036 |
| Aβ42, Aβ38, Aβ40/Aβ38, PTEDUCAT | 0.854 | 0.041 |
| Aβ38, Aβ42/Aβ40, Aβ42/Aβ38, Aβ40/Aβ38, PTEDUCAT, PTGENDER, APOE4 | 0.855 | 0.041 |
| Aβ40, Aβ42/Aβ40, Aβ42/Aβ38, Aβ40/Aβ38, PTEDUCAT, PTGENDER, APOE4 | 0.856 | 0.043 |
| Aβ42, Aβ38, AGE | 0.853 | 0.040 |
| Aβ42, Aβ38, Aβ42/Aβ40, Aβ40/Aβ38, AGE, PTGENDER, APOE4 | 0.861 | 0.047 |
| Aβ40, Aβ42/Aβ40, Aβ40/Aβ38, PTGENDER | 0.867 | 0.054 |
| Aβ40, Aβ38, Aβ42/Aβ40, Aβ42/Aβ38, PTGENDER, APOE4 | 0.855 | 0.042 |
| Aβ42, Aβ38, Aβ42/Aβ40, Aβ42/Aβ38, AGE, PTGENDER | 0.863 | 0.050 |
| Aβ42, Aβ38, Aβ42/Aβ38, Aβ40/Aβ38, AGE, APOE4 | 0.862 | 0.049 |
| Aβ42, Aβ38, Aβ42/Aβ38, Aβ40/Aβ38, AGE, PTEDUCAT, APOE4 | 0.851 | 0.037 |
| Aβ42, Aβ42/Aβ38, AGE, APOE4 | 0.856 | 0.043 |
| Aβ42, Aβ40, Aβ38, AGE, PTEDUCAT, APOE4 | 0.857 | 0.044 |
| Aβ42, Aβ40, Aβ38, AGE, PTGENDER | 0.849 | 0.036 |
| Aβ38, Aβ42/Aβ38, Aβ40/Aβ38, PTGENDER, APOE4 | 0.865 | 0.052 |
| Aβ38, Aβ42/Aβ38, Aβ40/Aβ38, AGE, PTEDUCAT, APOE4 | 0.872 | 0.059 |
| Aβ42, Aβ40, Aβ40/Aβ38, AGE, PTGENDER, APOE4 | 0.860 | 0.047 |
| Aβ42, Aβ40, Aβ42/Aβ40, Aβ42/Aβ38, Aβ40/Aβ38, AGE, PTGENDER | 0.853 | 0.040 |
| Aβ40, Aβ42/Aβ38, PTGENDER, APOE4 | 0.846 | 0.034 |
| Aβ42, Aβ38, Aβ42/Aβ38, AGE, PTEDUCAT, APOE4 | 0.858 | 0.045 |
| Aβ42, Aβ38, Aβ42/Aβ38, Aβ40/Aβ38, PTEDUCAT, PTGENDER | 0.860 | 0.048 |
| Aβ42, Aβ40, Aβ38, Aβ42/Aβ38, PTEDUCAT, PTGENDER, APOE4 | 0.855 | 0.042 |
| Aβ42, Aβ38, Aβ40/Aβ38, PTEDUCAT, APOE4 | 0.856 | 0.044 |
| Aβ42, Aβ40, PTEDUCAT, PTGENDER, APOE4 | 0.854 | 0.042 |
| Aβ38, Aβ42/Aβ40, Aβ42/Aβ38, Aβ40/Aβ38, AGE, PTEDUCAT, PTGENDER | 0.854 | 0.042 |
| Aβ42, Aβ40, Aβ42/Aβ40, Aβ42/Aβ38, PTGENDER, APOE4 | 0.845 | 0.033 |
| Aβ42, Aβ38 | 0.854 | 0.042 |
| Aβ40, Aβ42/Aβ38, PTEDUCAT, PTGENDER, APOE4 | 0.863 | 0.052 |
| Aβ42, Aβ40, Aβ38, Aβ40/Aβ38, APOE4 | 0.841 | 0.030 |
| Aβ40, Aβ38, Aβ42/Aβ40, PTGENDER, APOE4 | 0.855 | 0.044 |
| Aβ38, Aβ42/Aβ40, Aβ42/Aβ38, Aβ40/Aβ38, AGE | 0.851 | 0.040 |
| Aβ40, Aβ42/Aβ40, Aβ42/Aβ38, AGE, PTEDUCAT, PTGENDER, APOE4 | 0.863 | 0.052 |
| Aβ42, Aβ40, Aβ40/Aβ38, AGE, APOE4 | 0.845 | 0.034 |
| Aβ42, Aβ40, Aβ38, Aβ42/Aβ38, Aβ40/Aβ38, PTGENDER, APOE4 | 0.856 | 0.046 |
| Aβ40, Aβ42/Aβ38, PTGENDER | 0.870 | 0.059 |
| Aβ38, Aβ42/Aβ40, PTEDUCAT, PTGENDER, APOE4 | 0.855 | 0.044 |
| Aβ40, Aβ42/Aβ40, PTGENDER, APOE4 | 0.855 | 0.044 |
| Aβ40, Aβ38, Aβ42/Aβ38, PTGENDER, APOE4 | 0.853 | 0.043 |
| Aβ42, Aβ40, Aβ42/Aβ40, Aβ42/Aβ38, Aβ40/Aβ38, AGE, APOE4 | 0.845 | 0.035 |
| Aβ42, Aβ38, Aβ42/Aβ40, Aβ42/Aβ38, Aβ40/Aβ38, AGE, PTGENDER, APOE4 | 0.849 | 0.039 |
| Aβ42, Aβ40, Aβ38, Aβ42/Aβ40, PTGENDER, APOE4 | 0.858 | 0.047 |
| Aβ38, Aβ42/Aβ40, Aβ42/Aβ38, Aβ40/Aβ38, PTEDUCAT, PTGENDER | 0.854 | 0.043 |
| Aβ38, Aβ42/Aβ40, Aβ42/Aβ38, Aβ40/Aβ38, PTGENDER, APOE4 | 0.860 | 0.050 |
| Aβ42, Aβ40, Aβ40/Aβ38, PTGENDER, APOE4 | 0.852 | 0.042 |
| Aβ40, Aβ42/Aβ40, PTEDUCAT, PTGENDER, APOE4 | 0.862 | 0.052 |
| Aβ42, Aβ38, Aβ42/Aβ38, PTEDUCAT, PTGENDER | 0.841 | 0.031 |
| Aβ38, Aβ42/Aβ40, Aβ40/Aβ38, AGE, PTGENDER, APOE4 | 0.866 | 0.056 |
| Aβ42, Aβ40, PTGENDER | 0.860 | 0.050 |
| Aβ40, Aβ42/Aβ38, AGE, PTEDUCAT, PTGENDER, APOE4 | 0.861 | 0.051 |
| Aβ42, Aβ40, Aβ40/Aβ38, AGE, PTGENDER | 0.851 | 0.041 |
| Aβ38, Aβ42/Aβ38, Aβ40/Aβ38, AGE, APOE4 | 0.836 | 0.026 |
| Aβ42, Aβ40, AGE, PTEDUCAT, PTGENDER | 0.856 | 0.047 |
| Aβ42, Aβ40, Aβ42/Aβ40, Aβ42/Aβ38, Aβ40/Aβ38, PTGENDER | 0.863 | 0.054 |
| Aβ40, Aβ42/Aβ38, AGE, PTGENDER | 0.863 | 0.053 |
| Aβ42, Aβ40, Aβ38, Aβ42/Aβ40, Aβ40/Aβ38, AGE, PTGENDER, APOE4 | 0.846 | 0.037 |
| Aβ38, Aβ42/Aβ38, AGE, PTEDUCAT, PTGENDER | 0.857 | 0.048 |
| Aβ42, Aβ38, Aβ42/Aβ38, PTEDUCAT, APOE4 | 0.855 | 0.047 |
| Aβ42, Aβ40, Aβ38, Aβ40/Aβ38, AGE, PTEDUCAT, PTGENDER, APOE4 | 0.852 | 0.043 |
| Aβ40, Aβ42/Aβ40, Aβ40/Aβ38, AGE, PTGENDER | 0.858 | 0.049 |
| Aβ38, Aβ42/Aβ40, AGE, PTEDUCAT, PTGENDER | 0.864 | 0.056 |
| Aβ38, Aβ42/Aβ40, PTGENDER, APOE4 | 0.855 | 0.046 |
| Aβ38, Aβ42/Aβ40, Aβ42/Aβ38, PTGENDER | 0.856 | 0.048 |
| Aβ38, Aβ42/Aβ40, AGE, PTGENDER, APOE4 | 0.853 | 0.045 |
| Aβ40, Aβ42/Aβ40, Aβ42/Aβ38, PTEDUCAT, PTGENDER | 0.859 | 0.050 |
| Aβ40, Aβ42/Aβ40, PTGENDER | 0.857 | 0.048 |
| Aβ40, Aβ42/Aβ40, AGE, PTEDUCAT, PTGENDER, APOE4 | 0.859 | 0.051 |
| Aβ42, Aβ40, Aβ38, Aβ42/Aβ40, AGE, PTGENDER, APOE4 | 0.844 | 0.036 |
| Aβ42, Aβ42/Aβ38, AGE, PTEDUCAT, PTGENDER, APOE4 | 0.845 | 0.037 |
| Aβ42, Aβ38, Aβ42/Aβ38, AGE, APOE4 | 0.854 | 0.046 |
| Aβ42, Aβ38, Aβ42/Aβ38, AGE, PTEDUCAT, PTGENDER | 0.850 | 0.042 |
| Aβ38, Aβ42/Aβ40, PTGENDER | 0.851 | 0.043 |
| Aβ42, Aβ38, Aβ42/Aβ40, Aβ42/Aβ38, PTGENDER, APOE4 | 0.859 | 0.051 |
| Aβ40, Aβ38, Aβ42/Aβ40, Aβ42/Aβ38, AGE, PTGENDER, APOE4 | 0.865 | 0.057 |
| Aβ40, Aβ42/Aβ40, Aβ42/Aβ38, AGE, APOE4 | 0.852 | 0.045 |
| Aβ42, Aβ40, Aβ38, Aβ40/Aβ38, AGE, APOE4 | 0.844 | 0.036 |
| Aβ38, Aβ42/Aβ40, Aβ40/Aβ38, AGE, APOE4 | 0.842 | 0.034 |
| Aβ42, Aβ40, Aβ40/Aβ38, AGE, PTEDUCAT, PTGENDER | 0.860 | 0.053 |
| Aβ38, Aβ42/Aβ40, Aβ42/Aβ38, Aβ40/Aβ38, AGE, PTGENDER | 0.862 | 0.055 |
| Aβ40, Aβ42/Aβ40, AGE, PTGENDER | 0.861 | 0.053 |
| Aβ40, Aβ42/Aβ40, Aβ42/Aβ38, Aβ40/Aβ38, PTGENDER | 0.849 | 0.042 |
| Aβ42, Aβ38, PTEDUCAT, APOE4 | 0.855 | 0.048 |
| Aβ38, Aβ42/Aβ38, AGE, APOE4 | 0.835 | 0.028 |
| Aβ42, Aβ40, AGE, PTEDUCAT, PTGENDER, APOE4 | 0.852 | 0.045 |
| Aβ40, Aβ42/Aβ38, Aβ40/Aβ38, AGE, PTEDUCAT, PTGENDER, APOE4 | 0.855 | 0.049 |
| Aβ42, Aβ40, Aβ42/Aβ38, PTGENDER, APOE4 | 0.847 | 0.041 |
| Aβ42, Aβ42/Aβ38, AGE, PTGENDER | 0.850 | 0.043 |
| Aβ42, Aβ40, Aβ38, PTGENDER | 0.852 | 0.046 |
| Aβ40, Aβ42/Aβ40, Aβ42/Aβ38, AGE, PTGENDER | 0.863 | 0.057 |
| Aβ42, Aβ40, Aβ38, Aβ42/Aβ38, PTGENDER, APOE4 | 0.839 | 0.032 |
| Aβ42, Aβ40, PTGENDER, APOE4 | 0.859 | 0.052 |
| Aβ42, Aβ40, Aβ42/Aβ38, PTGENDER | 0.856 | 0.050 |
| Aβ38, Aβ42/Aβ40, Aβ42/Aβ38, Aβ40/Aβ38, AGE, PTEDUCAT, APOE4 | 0.852 | 0.046 |
| Aβ38, Aβ42/Aβ38, APOE4 | 0.844 | 0.039 |
| Aβ38, Aβ42/Aβ40, Aβ42/Aβ38, PTGENDER, APOE4 | 0.852 | 0.046 |
| Aβ42, Aβ40, Aβ38, AGE, PTEDUCAT, PTGENDER, APOE4 | 0.853 | 0.047 |
| Aβ38, Aβ42/Aβ40, Aβ42/Aβ38, Aβ40/Aβ38, PTGENDER | 0.847 | 0.042 |
| Aβ40, Aβ42/Aβ40, AGE, PTGENDER, APOE4 | 0.857 | 0.052 |
| Aβ42, Aβ40, Aβ42/Aβ40, Aβ40/Aβ38, AGE, PTGENDER, APOE4 | 0.853 | 0.048 |
| Aβ42, Aβ40, Aβ38, Aβ40/Aβ38, PTEDUCAT, PTGENDER, APOE4 | 0.840 | 0.035 |
| Aβ42, Aβ40, Aβ38, Aβ40/Aβ38, PTGENDER | 0.844 | 0.039 |
| Aβ42, Aβ40, AGE, PTGENDER | 0.858 | 0.053 |
| Aβ40, Aβ42/Aβ40, Aβ42/Aβ38, Aβ40/Aβ38, AGE, PTEDUCAT, PTGENDER, APOE4 | 0.849 | 0.045 |
| Aβ40, Aβ42/Aβ40, Aβ40/Aβ38, PTGENDER, APOE4 | 0.843 | 0.039 |
| Aβ42, Aβ40, AGE, PTGENDER, APOE4 | 0.852 | 0.049 |
| Aβ38, Aβ42/Aβ40, Aβ40/Aβ38, AGE, PTEDUCAT, PTGENDER, APOE4 | 0.857 | 0.054 |
| Aβ40, Aβ42/Aβ40, Aβ42/Aβ38, PTEDUCAT, PTGENDER, APOE4 | 0.856 | 0.053 |
| Aβ42, Aβ40, Aβ42/Aβ40, Aβ42/Aβ38, AGE, PTGENDER, APOE4 | 0.856 | 0.053 |
| Aβ42, Aβ40, Aβ40/Aβ38, PTGENDER | 0.845 | 0.042 |
| Aβ42, Aβ40, PTEDUCAT, PTGENDER | 0.851 | 0.048 |
| Aβ42, Aβ40, Aβ40/Aβ38, PTEDUCAT, PTGENDER | 0.851 | 0.048 |
| Aβ42, Aβ40, APOE4 | 0.846 | 0.044 |
| Aβ38, Aβ42/Aβ40, AGE, PTGENDER | 0.859 | 0.058 |
| Aβ42, Aβ40, Aβ40/Aβ38, PTEDUCAT, PTGENDER, APOE4 | 0.844 | 0.042 |
| Aβ42, Aβ40, Aβ42/Aβ40, Aβ42/Aβ38, Aβ40/Aβ38, AGE, PTGENDER, APOE4 | 0.837 | 0.036 |
| Aβ42, Aβ38, Aβ42/Aβ38, APOE4 | 0.853 | 0.052 |
| Aβ42, Aβ40, Aβ42/Aβ40, PTGENDER | 0.858 | 0.057 |
| Aβ42, Aβ38, Aβ40/Aβ38, AGE, PTGENDER, APOE4 | 0.850 | 0.049 |
| Aβ42, Aβ38, Aβ40/Aβ38, AGE, APOE4 | 0.850 | 0.050 |
| Aβ38, Aβ42/Aβ38, AGE, PTGENDER, APOE4 | 0.842 | 0.042 |
| Aβ42, Aβ38, Aβ42/Aβ38, PTGENDER | 0.849 | 0.050 |
| Aβ38, Aβ42/Aβ38, AGE, PTEDUCAT, APOE4 | 0.853 | 0.053 |
| Aβ42, Aβ40, Aβ38, PTGENDER, APOE4 | 0.843 | 0.044 |
| Aβ40, Aβ42/Aβ40, Aβ42/Aβ38, PTGENDER, APOE4 | 0.845 | 0.045 |
| Aβ42, Aβ40, Aβ40/Aβ38, AGE, PTEDUCAT, PTGENDER, APOE4 | 0.840 | 0.041 |
| Aβ42, Aβ38, PTEDUCAT | 0.851 | 0.052 |
| Aβ42, Aβ40, Aβ38, AGE, PTGENDER, APOE4 | 0.840 | 0.041 |
| Aβ38, Aβ42/Aβ40, AGE, PTEDUCAT, PTGENDER, APOE4 | 0.858 | 0.059 |
| Aβ42, Aβ40, Aβ38, Aβ40/Aβ38, AGE, PTGENDER, APOE4 | 0.852 | 0.053 |
| Aβ42, Aβ42/Aβ38, PTGENDER, APOE4 | 0.848 | 0.049 |
| Aβ38, Aβ42/Aβ38, PTGENDER | 0.847 | 0.048 |
| Aβ40, Aβ42/Aβ40, Aβ42/Aβ38, Aβ40/Aβ38, AGE, PTGENDER, APOE4 | 0.861 | 0.063 |
| Aβ38, Aβ42/Aβ40, Aβ42/Aβ38, AGE, PTGENDER | 0.851 | 0.053 |
| Aβ40, Aβ42/Aβ40, Aβ42/Aβ38, AGE, PTGENDER, APOE4 | 0.854 | 0.055 |
| Aβ38, Aβ42/Aβ38, PTEDUCAT, APOE4 | 0.850 | 0.052 |
| Aβ38, Aβ42/Aβ40, Aβ42/Aβ38, Aβ40/Aβ38, AGE, PTEDUCAT, PTGENDER, APOE4 | 0.844 | 0.046 |
| Aβ42, Aβ38, Aβ40/Aβ38, AGE, PTEDUCAT, APOE4 | 0.850 | 0.052 |
| Aβ42, Aβ38, Aβ40/Aβ38, PTGENDER, APOE4 | 0.833 | 0.036 |
| Aβ42, Aβ38, Aβ42/Aβ38, PTEDUCAT, PTGENDER, APOE4 | 0.842 | 0.045 |
| Aβ42, Aβ40, Aβ38, PTEDUCAT, PTGENDER, APOE4 | 0.838 | 0.041 |
| Aβ42, Aβ40, Aβ38, Aβ40/Aβ38, PTGENDER, APOE4 | 0.829 | 0.033 |
| Aβ42, Aβ38, AGE, APOE4 | 0.840 | 0.045 |
| Aβ42, Aβ38, Aβ42/Aβ38, AGE, PTEDUCAT, PTGENDER, APOE4 | 0.846 | 0.051 |
| Aβ38, Aβ42/Aβ38, AGE, PTGENDER | 0.842 | 0.047 |
| Aβ42, Aβ38, Aβ40/Aβ38, AGE, PTEDUCAT, PTGENDER | 0.851 | 0.056 |
| Aβ42, Aβ38, Aβ40/Aβ38, APOE4 | 0.831 | 0.036 |
| Aβ42, Aβ40, Aβ42/Aβ40, Aβ40/Aβ38, PTGENDER, APOE4 | 0.856 | 0.061 |
| Aβ42, Aβ42/Aβ38, PTGENDER | 0.843 | 0.049 |
| Aβ42, Aβ38, AGE, PTEDUCAT, PTGENDER | 0.841 | 0.047 |
| Aβ42, Aβ40, Aβ42/Aβ38, AGE, PTGENDER, APOE4 | 0.842 | 0.049 |
| Aβ42, Aβ38, Aβ40/Aβ38, AGE, PTGENDER | 0.847 | 0.055 |
| Aβ42, Aβ40, Aβ42/Aβ40, Aβ42/Aβ38, PTGENDER | 0.855 | 0.062 |
| Aβ38, Aβ42/Aβ40, Aβ42/Aβ38, AGE, PTGENDER, APOE4 | 0.845 | 0.053 |
| Aβ42, Aβ38, Aβ42/Aβ38, PTGENDER, APOE4 | 0.832 | 0.040 |
| Aβ38, Aβ42/Aβ38, PTEDUCAT, PTGENDER, APOE4 | 0.837 | 0.045 |
| Aβ40, Aβ38, Aβ40/Aβ38, AGE, PTEDUCAT, APOE4 | 0.816 | 0.025 |
| Aβ38, Aβ42/Aβ38, PTGENDER, APOE4 | 0.832 | 0.041 |
| Aβ42, Aβ42/Aβ38, AGE, PTGENDER, APOE4 | 0.843 | 0.052 |
| Aβ42, Aβ38, Aβ42/Aβ38, AGE, PTGENDER | 0.842 | 0.052 |
| Aβ40, Aβ42/Aβ40, Aβ42/Aβ38, PTGENDER | 0.852 | 0.062 |
| Aβ42, Aβ38, Aβ40/Aβ38, PTGENDER | 0.845 | 0.056 |
| Aβ42, Aβ38, AGE, PTGENDER | 0.837 | 0.049 |
| Aβ42, Aβ38, AGE, PTEDUCAT | 0.849 | 0.061 |
| Aβ42, Aβ40, Aβ42/Aβ40, PTGENDER, APOE4 | 0.842 | 0.054 |
| Aβ42, Aβ38, Aβ42/Aβ38, AGE, PTGENDER, APOE4 | 0.829 | 0.041 |
| Aβ38, Aβ42/Aβ40, Aβ42/Aβ38, Aβ40/Aβ38, AGE, PTGENDER, APOE4 | 0.837 | 0.049 |
| Aβ40, Aβ38, Aβ40/Aβ38, PTEDUCAT, APOE4 | 0.810 | 0.026 |
| Aβ40, Aβ38, AGE, PTEDUCAT, APOE4 | 0.816 | 0.032 |
| Aβ38, Aβ40/Aβ38, AGE, PTEDUCAT, APOE4 | 0.809 | 0.026 |
| Aβ42, Aβ38, AGE, PTGENDER, APOE4 | 0.830 | 0.047 |
| Aβ40, Aβ38, Aβ40/Aβ38, AGE, PTEDUCAT | 0.802 | 0.020 |
| Aβ40, Aβ40/Aβ38, AGE, PTEDUCAT, APOE4 | 0.803 | 0.022 |
| Aβ38, Aβ42/Aβ38, AGE, PTEDUCAT, PTGENDER, APOE4 | 0.846 | 0.065 |
| Aβ40, Aβ38, PTEDUCAT, APOE4 | 0.813 | 0.033 |
| Aβ40, Aβ38, Aβ40/Aβ38, AGE, APOE4 | 0.805 | 0.026 |
| Aβ42, Aβ38, PTEDUCAT, PTGENDER, APOE4 | 0.825 | 0.046 |
| Aβ42, Aβ38, AGE, PTEDUCAT, APOE4 | 0.843 | 0.064 |
| Aβ42, Aβ38, PTGENDER | 0.817 | 0.039 |
| Aβ42, Aβ38, PTGENDER, APOE4 | 0.804 | 0.026 |
| Aβ42, Aβ38, AGE, PTEDUCAT, PTGENDER, APOE4 | 0.831 | 0.053 |
| Aβ38, AGE, PTEDUCAT, APOE4 | 0.817 | 0.041 |
| Aβ40, AGE, PTEDUCAT, APOE4 | 0.812 | 0.036 |
| Aβ40, Aβ38, AGE, PTEDUCAT | 0.805 | 0.031 |
| Aβ40, Aβ38, AGE, APOE4 | 0.809 | 0.035 |
| Aβ40, Aβ38, Aβ40/Aβ38, AGE, PTEDUCAT, PTGENDER, APOE4 | 0.809 | 0.036 |
| Aβ40, Aβ38, Aβ40/Aβ38, PTEDUCAT | 0.795 | 0.022 |
| Aβ42, Aβ38, PTEDUCAT, PTGENDER | 0.830 | 0.058 |
| Aβ38, AGE, APOE4 | 0.811 | 0.038 |
| Aβ40, Aβ38, AGE, PTEDUCAT, PTGENDER, APOE4 | 0.813 | 0.041 |
| Aβ40, Aβ40/Aβ38, AGE, APOE4 | 0.825 | 0.054 |
| Aβ40, Aβ38, Aβ40/Aβ38, APOE4 | 0.796 | 0.025 |
| Aβ40, PTEDUCAT, APOE4 | 0.800 | 0.030 |
| Aβ38, Aβ40/Aβ38, AGE, APOE4 | 0.799 | 0.028 |
| Aβ38, Aβ40/Aβ38, AGE, PTEDUCAT | 0.792 | 0.022 |
| Aβ40, Aβ38, PTEDUCAT | 0.802 | 0.033 |
| Aβ40, Aβ38, AGE, PTEDUCAT, PTGENDER | 0.804 | 0.036 |
| Aβ40, Aβ38, Aβ40/Aβ38, AGE | 0.802 | 0.034 |
| Aβ40, Aβ38, Aβ40/Aβ38, AGE, PTEDUCAT, PTGENDER | 0.796 | 0.028 |
| Aβ40, Aβ38, APOE4 | 0.801 | 0.033 |
| Aβ40, Aβ40/Aβ38, PTEDUCAT, APOE4 | 0.796 | 0.028 |
| Aβ38, PTEDUCAT | 0.801 | 0.034 |
| Aβ40, Aβ40/Aβ38, AGE, PTEDUCAT | 0.801 | 0.034 |
| Aβ38, PTEDUCAT, APOE4 | 0.809 | 0.042 |
| Aβ38, Aβ40/Aβ38, AGE, PTEDUCAT, PTGENDER, APOE4 | 0.816 | 0.050 |
| Aβ40, Aβ40/Aβ38, PTEDUCAT, PTGENDER | 0.810 | 0.044 |
| Aβ38, AGE, PTEDUCAT, PTGENDER | 0.804 | 0.039 |
| Aβ40, Aβ38, Aβ40/Aβ38, PTEDUCAT, PTGENDER, APOE4 | 0.799 | 0.034 |
| Aβ38, Aβ40/Aβ38, PTEDUCAT, APOE4 | 0.798 | 0.034 |
| Aβ40, Aβ38, PTEDUCAT, PTGENDER, APOE4 | 0.806 | 0.042 |
| Aβ40, Aβ40/Aβ38, AGE, PTEDUCAT, PTGENDER | 0.795 | 0.032 |
| Aβ40, Aβ38, Aβ40/Aβ38, AGE, PTGENDER, APOE4 | 0.798 | 0.035 |
| Aβ40, Aβ40/Aβ38, AGE, PTGENDER | 0.803 | 0.039 |
| Aβ38, Aβ40/Aβ38, PTEDUCAT | 0.785 | 0.022 |
| Aβ40, Aβ40/Aβ38, AGE, PTEDUCAT, PTGENDER, APOE4 | 0.812 | 0.049 |
| Aβ40, Aβ40/Aβ38, APOE4 | 0.787 | 0.024 |
| Aβ38, AGE, PTEDUCAT | 0.796 | 0.033 |
| Aβ40, Aβ38, Aβ40/Aβ38, PTEDUCAT, PTGENDER | 0.786 | 0.024 |
| Aβ40, AGE, APOE4 | 0.807 | 0.045 |
| Aβ38, Aβ40/Aβ38, APOE4 | 0.789 | 0.027 |
| Aβ38, AGE | 0.794 | 0.033 |
| Aβ38, PTGENDER, APOE4 | 0.799 | 0.039 |
| Aβ40, Aβ38 | 0.789 | 0.029 |
| Aβ40, Aβ38, AGE | 0.795 | 0.036 |
| Aβ40, APOE4 | 0.793 | 0.034 |
| Aβ40, Aβ38, Aβ40/Aβ38 | 0.783 | 0.024 |
| Aβ40, Aβ38, Aβ40/Aβ38, AGE, PTGENDER | 0.798 | 0.040 |
| Aβ40, Aβ38, AGE, PTGENDER, APOE4 | 0.801 | 0.043 |
| Aβ40, Aβ40/Aβ38 | 0.786 | 0.028 |
| Aβ40, Aβ40/Aβ38, AGE | 0.793 | 0.035 |
| Aβ40, PTEDUCAT, PTGENDER | 0.785 | 0.028 |
| Aβ40, Aβ40/Aβ38, PTEDUCAT | 0.783 | 0.026 |
| Aβ40, AGE, PTEDUCAT | 0.798 | 0.042 |
| Aβ40, Aβ38, PTGENDER, APOE4 | 0.795 | 0.039 |
| Aβ40, Aβ38, PTEDUCAT, PTGENDER | 0.793 | 0.038 |
| Aβ40, Aβ38, Aβ40/Aβ38, PTGENDER, APOE4 | 0.787 | 0.032 |
| Aβ38, Aβ40/Aβ38 | 0.776 | 0.021 |
| Aβ38, AGE, PTEDUCAT, PTGENDER, APOE4 | 0.805 | 0.051 |
| Aβ38, Aβ40/Aβ38, AGE | 0.790 | 0.037 |
| Aβ40, Aβ40/Aβ38, AGE, PTGENDER, APOE4 | 0.806 | 0.052 |
| Aβ40, AGE, PTGENDER, APOE4 | 0.796 | 0.042 |
| Aβ38 | 0.787 | 0.034 |
| Aβ40, AGE, PTEDUCAT, PTGENDER, APOE4 | 0.799 | 0.047 |
| Aβ38, APOE4 | 0.793 | 0.041 |
| Aβ38, PTEDUCAT, PTGENDER | 0.783 | 0.032 |
| Aβ40, PTEDUCAT, PTGENDER, APOE4 | 0.793 | 0.042 |
| Aβ38, Aβ40/Aβ38, PTGENDER, APOE4 | 0.806 | 0.055 |
| Aβ40, Aβ38, AGE, PTGENDER | 0.789 | 0.040 |
| Aβ40, AGE | 0.788 | 0.038 |
| Aβ40, PTEDUCAT | 0.789 | 0.040 |
| Aβ38, Aβ40/Aβ38, AGE, PTEDUCAT, PTGENDER | 0.778 | 0.029 |
| Aβ38, PTGENDER | 0.776 | 0.027 |
| Aβ40, Aβ38, Aβ40/Aβ38, PTGENDER | 0.776 | 0.028 |
| Aβ40, Aβ40/Aβ38, PTGENDER, APOE4 | 0.776 | 0.029 |
| Aβ40, Aβ40/Aβ38, PTEDUCAT, PTGENDER, APOE4 | 0.791 | 0.043 |
| Aβ40 | 0.782 | 0.036 |
| Aβ38, Aβ40/Aβ38, PTEDUCAT, PTGENDER | 0.784 | 0.038 |
| Aβ38, PTEDUCAT, PTGENDER, APOE4 | 0.786 | 0.041 |
| Aβ38, Aβ40/Aβ38, AGE, PTGENDER | 0.768 | 0.024 |
| Aβ38, Aβ40/Aβ38, AGE, PTGENDER, APOE4 | 0.784 | 0.041 |
| Aβ40, AGE, PTEDUCAT, PTGENDER | 0.795 | 0.052 |
| Aβ40, Aβ38, PTGENDER | 0.781 | 0.038 |
| Aβ38, Aβ40/Aβ38, PTEDUCAT, PTGENDER, APOE4 | 0.785 | 0.046 |
| Aβ38, AGE, PTGENDER, APOE4 | 0.799 | 0.061 |
| Aβ38, AGE, PTGENDER | 0.778 | 0.040 |
| Aβ40, PTGENDER, APOE4 | 0.785 | 0.049 |
| Aβ40, AGE, PTGENDER | 0.786 | 0.050 |
| Aβ38, Aβ40/Aβ38, PTGENDER | 0.782 | 0.048 |
| Aβ42/Aβ40, Aβ40/Aβ38, AGE, PTEDUCAT | 0.789 | 0.059 |
| Aβ40, Aβ40/Aβ38, PTGENDER | 0.769 | 0.040 |
| Aβ42/Aβ40, Aβ42/Aβ38, Aβ40/Aβ38, AGE, PTEDUCAT, PTGENDER | 0.757 | 0.034 |
| Aβ42/Aβ40, Aβ42/Aβ38, Aβ40/Aβ38, AGE, PTEDUCAT, PTGENDER, APOE4 | 0.757 | 0.034 |
| Aβ42/Aβ40, Aβ42/Aβ38, Aβ40/Aβ38, AGE, PTEDUCAT, APOE4 | 0.757 | 0.034 |
| Aβ40, PTGENDER | 0.771 | 0.053 |
| Aβ42/Aβ40, Aβ40/Aβ38, AGE, PTEDUCAT, APOE4 | 0.777 | 0.065 |
| Aβ42/Aβ40, Aβ40/Aβ38, AGE, PTGENDER | 0.759 | 0.050 |
| Aβ42/Aβ40, Aβ40/Aβ38, AGE, PTGENDER, APOE4 | 0.759 | 0.050 |
| Aβ42/Aβ38, Aβ40/Aβ38, AGE, APOE4 | 0.725 | 0.020 |
| Aβ42/Aβ38, Aβ40/Aβ38, AGE, PTEDUCAT, APOE4 | 0.741 | 0.036 |
| Aβ42/Aβ38, Aβ40/Aβ38, AGE | 0.724 | 0.020 |
| Aβ42/Aβ38, Aβ40/Aβ38, AGE, PTEDUCAT, PTGENDER | 0.713 | 0.010 |
| Aβ42/Aβ40, Aβ40/Aβ38, AGE, APOE4 | 0.753 | 0.051 |
| Aβ42/Aβ38, Aβ40/Aβ38 | 0.768 | 0.067 |
| Aβ42/Aβ40, Aβ40/Aβ38, AGE | 0.752 | 0.051 |
| Aβ42/Aβ40, Aβ40/Aβ38, PTEDUCAT | 0.748 | 0.048 |
| Aβ42/Aβ38, Aβ40/Aβ38, AGE, PTEDUCAT | 0.723 | 0.023 |
| Aβ42/Aβ38, Aβ40/Aβ38, AGE, PTEDUCAT, PTGENDER, APOE4 | 0.723 | 0.023 |
| Aβ42/Aβ40, Aβ42/Aβ38, Aβ40/Aβ38, AGE, PTEDUCAT | 0.748 | 0.048 |
| Aβ42/Aβ38, Aβ40/Aβ38, AGE, PTGENDER | 0.718 | 0.022 |
| Aβ42/Aβ40, Aβ40/Aβ38, PTEDUCAT, APOE4 | 0.754 | 0.061 |
| Aβ42/Aβ40, Aβ40/Aβ38, AGE, PTEDUCAT, PTGENDER | 0.768 | 0.075 |
| Aβ42/Aβ40, Aβ40/Aβ38, AGE, PTEDUCAT, PTGENDER, APOE4 | 0.768 | 0.075 |
| Aβ42/Aβ40, Aβ40/Aβ38, PTEDUCAT, PTGENDER | 0.739 | 0.048 |
| Aβ42/Aβ40, Aβ40/Aβ38 | 0.754 | 0.063 |
| Aβ42/Aβ38, Aβ40/Aβ38, AGE, PTGENDER, APOE4 | 0.744 | 0.057 |
| Aβ42/Aβ38, Aβ40/Aβ38, PTEDUCAT | 0.742 | 0.054 |
| Aβ42/Aβ38, Aβ40/Aβ38, PTEDUCAT, APOE4 | 0.742 | 0.054 |
| Aβ42/Aβ40, Aβ40/Aβ38, PTEDUCAT, PTGENDER, APOE4 | 0.754 | 0.073 |
| Aβ42/Aβ40, Aβ42/Aβ38, Aβ40/Aβ38, PTEDUCAT, APOE4 | 0.733 | 0.056 |
| Aβ42/Aβ40, Aβ42/Aβ38, Aβ40/Aβ38, PTEDUCAT | 0.730 | 0.057 |
| Aβ42/Aβ38, Aβ40/Aβ38, PTEDUCAT, PTGENDER | 0.731 | 0.061 |
| Aβ42/Aβ38, Aβ40/Aβ38, PTEDUCAT, PTGENDER, APOE4 | 0.731 | 0.061 |
| Aβ42/Aβ40, Aβ40/Aβ38, APOE4 | 0.741 | 0.073 |
| Aβ42/Aβ40, Aβ42/Aβ38, Aβ40/Aβ38, AGE | 0.731 | 0.064 |
| Aβ42/Aβ40, Aβ40/Aβ38, PTGENDER | 0.733 | 0.068 |
| Aβ42/Aβ40, Aβ40/Aβ38, PTGENDER, APOE4 | 0.733 | 0.068 |
| Aβ42/Aβ40, Aβ42/Aβ38, Aβ40/Aβ38, PTEDUCAT, PTGENDER | 0.698 | 0.033 |
| Aβ42/Aβ40, Aβ42/Aβ38, Aβ40/Aβ38 | 0.744 | 0.080 |
| Aβ42, Aβ40/Aβ38, AGE, PTEDUCAT | 0.680 | 0.016 |
| Aβ42/Aβ40, Aβ42/Aβ38, Aβ40/Aβ38, AGE, PTGENDER | 0.723 | 0.060 |
| Aβ40/Aβ38, AGE, PTEDUCAT, PTGENDER, APOE4 | 0.694 | 0.035 |
| Aβ42/Aβ40, Aβ42/Aβ38, Aβ40/Aβ38, AGE, PTGENDER, APOE4 | 0.719 | 0.060 |
| Aβ40/Aβ38, AGE, PTEDUCAT | 0.681 | 0.029 |
| Aβ42/Aβ38, Aβ40/Aβ38, PTGENDER, APOE4 | 0.727 | 0.078 |
| Aβ40/Aβ38, AGE, PTEDUCAT, PTGENDER | 0.680 | 0.032 |
| Aβ40/Aβ38, AGE, PTEDUCAT, APOE4 | 0.683 | 0.035 |
| Aβ42/Aβ38, Aβ40/Aβ38, APOE4 | 0.713 | 0.066 |
| Aβ42/Aβ40, Aβ42/Aβ38, Aβ40/Aβ38, PTGENDER | 0.723 | 0.081 |
| Aβ42/Aβ38, Aβ40/Aβ38, PTGENDER | 0.703 | 0.062 |
| Aβ42/Aβ40, Aβ42/Aβ38, Aβ40/Aβ38, APOE4 | 0.718 | 0.078 |
| Aβ42/Aβ40, Aβ42/Aβ38, Aβ40/Aβ38, AGE, APOE4 | 0.716 | 0.077 |
| Aβ42/Aβ40, Aβ42/Aβ38, Aβ40/Aβ38, PTEDUCAT, PTGENDER, APOE4 | 0.734 | 0.096 |
| Aβ42, Aβ40/Aβ38, AGE, PTGENDER | 0.672 | 0.036 |
| Aβ40/Aβ38, AGE, PTGENDER, APOE4 | 0.677 | 0.044 |
| Aβ42/Aβ40, Aβ42/Aβ38, Aβ40/Aβ38, PTGENDER, APOE4 | 0.713 | 0.083 |
| Aβ42, Aβ40/Aβ38, AGE, PTGENDER, APOE4 | 0.695 | 0.066 |
| Aβ42/Aβ40, AGE, PTEDUCAT | 0.699 | 0.070 |
| Aβ42, Aβ40/Aβ38, AGE, APOE4 | 0.662 | 0.034 |
| Aβ42, Aβ40/Aβ38, AGE, PTEDUCAT, APOE4 | 0.664 | 0.038 |
| Aβ42/Aβ40, AGE, PTEDUCAT, APOE4 | 0.703 | 0.078 |
| Aβ42, Aβ40/Aβ38, AGE, PTEDUCAT, PTGENDER, APOE4 | 0.663 | 0.037 |
| Aβ40/Aβ38, AGE | 0.695 | 0.073 |
| Aβ40/Aβ38, AGE, APOE4 | 0.667 | 0.045 |
| Aβ42, Aβ40/Aβ38, APOE4 | 0.657 | 0.036 |
| Aβ42/Aβ38, AGE, PTEDUCAT | 0.696 | 0.076 |
| Aβ42/Aβ40, PTEDUCAT | 0.701 | 0.085 |
| Aβ40/Aβ38, PTEDUCAT, PTGENDER | 0.686 | 0.073 |
| Aβ40/Aβ38, AGE, PTGENDER | 0.636 | 0.023 |
| Aβ40/Aβ38, PTEDUCAT | 0.688 | 0.075 |
| Aβ42/Aβ38, AGE, PTEDUCAT, APOE4 | 0.699 | 0.087 |
| Aβ42/Aβ38 | 0.682 | 0.070 |
| Aβ42, AGE, APOE4 | 0.662 | 0.050 |
| Aβ42/Aβ40, Aβ42/Aβ38, AGE, PTEDUCAT | 0.699 | 0.087 |
| Aβ42/Aβ40, AGE | 0.686 | 0.074 |
| Aβ42/Aβ40, AGE, APOE4 | 0.696 | 0.085 |
| Aβ42/Aβ40, Aβ42/Aβ38, AGE | 0.683 | 0.072 |
| Aβ42/Aβ40, Aβ42/Aβ38, AGE, APOE4 | 0.693 | 0.083 |
| Aβ42/Aβ40, Aβ42/Aβ38, AGE, PTEDUCAT, APOE4 | 0.700 | 0.091 |
| Aβ42/Aβ40, PTEDUCAT, APOE4 | 0.691 | 0.084 |
| Aβ42/Aβ40, AGE, PTEDUCAT, PTGENDER | 0.690 | 0.085 |
| Aβ42, Aβ40/Aβ38, AGE | 0.654 | 0.051 |
| Aβ42/Aβ40, AGE, PTEDUCAT, PTGENDER, APOE4 | 0.684 | 0.082 |
| Aβ42/Aβ38, AGE, APOE4 | 0.697 | 0.095 |
| Aβ42/Aβ40, Aβ42/Aβ38, AGE, PTEDUCAT, PTGENDER | 0.695 | 0.094 |
| Aβ42/Aβ38, AGE | 0.679 | 0.079 |
| Aβ42/Aβ38, AGE, PTEDUCAT, PTGENDER | 0.687 | 0.088 |
| Aβ42/Aβ40, Aβ42/Aβ38, AGE, PTGENDER | 0.678 | 0.079 |
| Aβ42/Aβ40, Aβ42/Aβ38, PTEDUCAT | 0.698 | 0.100 |
| Aβ42/Aβ40, AGE, PTGENDER | 0.680 | 0.082 |
| Aβ42/Aβ38, AGE, PTGENDER | 0.669 | 0.072 |
| Aβ42/Aβ40, Aβ42/Aβ38, PTEDUCAT, APOE4 | 0.694 | 0.098 |
| Aβ40/Aβ38, APOE4 | 0.663 | 0.067 |
| Aβ42/Aβ38, PTEDUCAT | 0.692 | 0.096 |
| Aβ42/Aβ40, Aβ42/Aβ38, AGE, PTGENDER, APOE4 | 0.686 | 0.090 |
| Aβ42, AGE, PTEDUCAT, APOE4 | 0.642 | 0.048 |
| Aβ42/Aβ38, AGE, PTEDUCAT, PTGENDER, APOE4 | 0.689 | 0.095 |
| Aβ42/Aβ40, AGE, PTGENDER, APOE4 | 0.674 | 0.080 |
| Aβ42/Aβ40, Aβ42/Aβ38, AGE, PTEDUCAT, PTGENDER, APOE4 | 0.693 | 0.099 |
| Aβ42, Aβ40/Aβ38, PTEDUCAT, PTGENDER, APOE4 | 0.642 | 0.049 |
| Aβ42, Aβ40/Aβ38, AGE, PTEDUCAT, PTGENDER | 0.636 | 0.045 |
| Aβ42/Aβ38, PTEDUCAT, APOE4 | 0.687 | 0.097 |
| Aβ42/Aβ40 | 0.688 | 0.101 |
| Aβ42, AGE, PTEDUCAT, PTGENDER, APOE4 | 0.623 | 0.036 |
| Aβ42/Aβ40, APOE4 | 0.680 | 0.094 |
| Aβ42, Aβ40/Aβ38, PTEDUCAT | 0.644 | 0.059 |
| Aβ42, Aβ40/Aβ38, PTGENDER, APOE4 | 0.619 | 0.034 |
| Aβ42/Aβ40, Aβ42/Aβ38 | 0.657 | 0.073 |
| Aβ42/Aβ40, Aβ42/Aβ38, PTEDUCAT, PTGENDER | 0.692 | 0.108 |
| Aβ42, AGE, PTGENDER, APOE4 | 0.618 | 0.035 |
| Aβ40/Aβ38, PTGENDER, APOE4 | 0.629 | 0.047 |
| Aβ42, Aβ40/Aβ38, PTEDUCAT, APOE4 | 0.647 | 0.066 |
| Aβ42/Aβ40, Aβ42/Aβ38, APOE4 | 0.682 | 0.101 |
| Aβ42/Aβ38, AGE, PTGENDER, APOE4 | 0.685 | 0.106 |
| Aβ42/Aβ40, PTEDUCAT, PTGENDER | 0.684 | 0.104 |
| Aβ42/Aβ40, PTEDUCAT, PTGENDER, APOE4 | 0.679 | 0.101 |
| Aβ42/Aβ40, Aβ42/Aβ38, PTEDUCAT, PTGENDER, APOE4 | 0.685 | 0.108 |
| Aβ42, Aβ40/Aβ38, PTEDUCAT, PTGENDER | 0.639 | 0.063 |
| Aβ40/Aβ38, PTEDUCAT, APOE4 | 0.657 | 0.081 |
| AGE, PTEDUCAT, APOE4 | 0.620 | 0.045 |
| Aβ40/Aβ38 | 0.641 | 0.069 |
| Aβ42, AGE | 0.611 | 0.038 |
| Aβ42/Aβ38, PTEDUCAT, PTGENDER | 0.682 | 0.112 |
| Aβ42/Aβ38, PTGENDER | 0.661 | 0.091 |
| Aβ40/Aβ38, PTEDUCAT, PTGENDER, APOE4 | 0.664 | 0.094 |
| Aβ42/Aβ38, APOE4 | 0.671 | 0.102 |
| Aβ42, AGE, PTGENDER | 0.608 | 0.040 |
| Aβ42/Aβ40, Aβ42/Aβ38, PTGENDER | 0.625 | 0.058 |
| Aβ42/Aβ40, Aβ42/Aβ38, PTGENDER, APOE4 | 0.676 | 0.109 |
| Aβ42, Aβ40/Aβ38 | 0.633 | 0.068 |
| AGE, APOE4 | 0.644 | 0.080 |
| Aβ42/Aβ40, PTGENDER | 0.660 | 0.097 |
| Aβ42, AGE, PTEDUCAT | 0.598 | 0.036 |
| Aβ40/Aβ38, PTGENDER | 0.626 | 0.064 |
| Aβ42/Aβ38, PTEDUCAT, PTGENDER, APOE4 | 0.674 | 0.114 |
| Aβ42/Aβ40, PTGENDER, APOE4 | 0.626 | 0.067 |
| Aβ42/Aβ38, PTGENDER, APOE4 | 0.674 | 0.117 |
| Aβ42, AGE, PTEDUCAT, PTGENDER | 0.611 | 0.055 |
| Aβ42, Aβ40/Aβ38, PTGENDER | 0.598 | 0.043 |
| Aβ42, APOE4 | 0.643 | 0.088 |
| Aβ42 | 0.594 | 0.040 |
| Aβ42, PTEDUCAT, APOE4 | 0.631 | 0.090 |
| Aβ42, PTGENDER, APOE4 | 0.592 | 0.058 |
| AGE, PTEDUCAT | 0.560 | 0.027 |
| Aβ42, PTGENDER | 0.562 | 0.030 |
| AGE, PTEDUCAT, PTGENDER, APOE4 | 0.616 | 0.091 |
| Aβ42, PTEDUCAT | 0.570 | 0.047 |
| APOE4 | 0.557 | 0.036 |
| PTEDUCAT, APOE4 | 0.573 | 0.057 |
| Aβ42, PTEDUCAT, PTGENDER, APOE4 | 0.599 | 0.088 |
| Aβ42, PTEDUCAT, PTGENDER | 0.539 | 0.036 |
| AGE | 0.577 | 0.075 |
| AGE, PTGENDER, APOE4 | 0.608 | 0.108 |
| PTEDUCAT | 0.521 | 0.037 |
| AGE, PTEDUCAT, PTGENDER | 0.557 | 0.074 |
| PTEDUCAT, PTGENDER, APOE4 | 0.530 | 0.052 |
| PTEDUCAT, PTGENDER | 0.482 | 0.011 |
| AGE, PTGENDER | 0.546 | 0.099 |
| PTGENDER, APOE4 | 0.523 | 0.098 |
| PTGENDER | 0.468 | 0.069 |
