# Supplementary material for: Soluble amyloid-beta isoforms predict downstream Alzheimer’s disease pathology
Source: Cell Biosci. 2021 Dec 11;11:204. doi: 10.1186/s13578-021-00712-3 (PMC8665586; doi:10.1186/s13578-021-00712-3)
Supplement: Supplementary file 2 — Additional file 2. Machine learning results for predicting neurodegeneration positivity (N+). Table containing features, AUC and standard deviation results for all 1023 models for predicting neurodegeneration positivity. [file 13578_2021_712_MOESM2_ESM.docx]

Additional file 2

M**achine learning results for predicting neurodegeneration positivity (N+).**

| **Features** | **Acc** | **Stdev** |
| --- | --- | --- |
| Aβ42, Aβ40, Aβ42/Aβ40, Aβ42/Aβ38, Aβ40/Aβ38 | 0.915 | 0.018 |
| Aβ42, Aβ40, Aβ42/Aβ38, Aβ40/Aβ38, PTGENDER, APOE4 | 0.905 | 0.009 |
| Aβ42, Aβ40, Aβ42/Aβ40, Aβ40/Aβ38 | 0.918 | 0.022 |
| Aβ40, Aβ42/Aβ40, Aβ40/Aβ38, PTEDUCAT | 0.911 | 0.015 |
| Aβ42, Aβ40, Aβ42/Aβ40, Aβ40/Aβ38, PTEDUCAT | 0.913 | 0.017 |
| Aβ40, Aβ42/Aβ40, Aβ40/Aβ38 | 0.908 | 0.012 |
| Aβ40, Aβ42/Aβ40, Aβ40/Aβ38, APOE4 | 0.900 | 0.005 |
| Aβ40, Aβ42/Aβ40, PTGENDER, APOE4 | 0.906 | 0.012 |
| Aβ42, Aβ40, Aβ40/Aβ38 | 0.902 | 0.008 |
| Aβ42, Aβ42/Aβ40, AGE, APOE4 | 0.909 | 0.015 |
| Aβ42, Aβ40, Aβ42/Aβ38, Aβ40/Aβ38 | 0.907 | 0.014 |
| Aβ42, Aβ42/Aβ40 | 0.908 | 0.016 |
| Aβ42, Aβ40, Aβ40/Aβ38, PTGENDER | 0.906 | 0.013 |
| Aβ42, Aβ42/Aβ40, PTEDUCAT | 0.917 | 0.024 |
| Aβ40, Aβ38, Aβ42/Aβ40, Aβ40/Aβ38 | 0.913 | 0.021 |
| Aβ40, Aβ38, Aβ42/Aβ40, Aβ42/Aβ38, Aβ40/Aβ38, PTEDUCAT | 0.917 | 0.025 |
| Aβ42, Aβ40, Aβ42/Aβ40, Aβ40/Aβ38, PTGENDER | 0.900 | 0.008 |
| Aβ40, Aβ42/Aβ40, Aβ40/Aβ38, PTGENDER | 0.908 | 0.016 |
| Aβ42, Aβ42/Aβ40, Aβ42/Aβ38, Aβ40/Aβ38, AGE, PTEDUCAT | 0.911 | 0.020 |
| Aβ42, Aβ40, Aβ38, Aβ42/Aβ40, Aβ40/Aβ38 | 0.910 | 0.019 |
| Aβ42, Aβ42/Aβ40, Aβ42/Aβ38, Aβ40/Aβ38, PTEDUCAT | 0.907 | 0.015 |
| Aβ40, Aβ42/Aβ38, Aβ40/Aβ38 | 0.911 | 0.020 |
| Aβ40, Aβ42/Aβ40 | 0.912 | 0.021 |
| Aβ40, Aβ42/Aβ38 | 0.915 | 0.024 |
| Aβ42, Aβ40, Aβ42/Aβ40, Aβ42/Aβ38 | 0.913 | 0.022 |
| Aβ40, Aβ42/Aβ40, Aβ42/Aβ38, PTEDUCAT | 0.904 | 0.014 |
| Aβ42, Aβ40, Aβ42/Aβ40, AGE | 0.912 | 0.022 |
| Aβ40, Aβ42/Aβ38, PTGENDER | 0.915 | 0.026 |
| Aβ42, Aβ40, Aβ38, Aβ42/Aβ40, Aβ40/Aβ38, APOE4 | 0.907 | 0.018 |
| Aβ42, Aβ42/Aβ40, AGE | 0.911 | 0.022 |
| Aβ40, Aβ38, Aβ42/Aβ40, Aβ42/Aβ38, Aβ40/Aβ38, APOE4 | 0.915 | 0.026 |
| Aβ42, Aβ38, Aβ42/Aβ40, Aβ42/Aβ38, Aβ40/Aβ38 | 0.900 | 0.011 |
| Aβ42, Aβ40, Aβ38, Aβ42/Aβ38, Aβ40/Aβ38 | 0.913 | 0.024 |
| Aβ40, Aβ42/Aβ38, APOE4 | 0.911 | 0.022 |
| Aβ42, Aβ40, Aβ42/Aβ40 | 0.909 | 0.020 |
| Aβ42, Aβ40, Aβ38, Aβ42/Aβ40, Aβ40/Aβ38, AGE | 0.900 | 0.011 |
| Aβ42, Aβ40, Aβ42/Aβ40, AGE, PTEDUCAT | 0.911 | 0.022 |
| Aβ42, Aβ42/Aβ40, AGE, PTEDUCAT | 0.916 | 0.027 |
| Aβ42, Aβ40, Aβ42/Aβ40, Aβ40/Aβ38, PTEDUCAT, APOE4 | 0.903 | 0.014 |
| Aβ42, Aβ40, Aβ40/Aβ38, AGE, PTEDUCAT | 0.897 | 0.008 |
| Aβ40, Aβ38, Aβ42/Aβ40, Aβ42/Aβ38, Aβ40/Aβ38, PTGENDER | 0.916 | 0.027 |
| Aβ42, Aβ40, Aβ42/Aβ40, PTEDUCAT | 0.912 | 0.024 |
| Aβ42, Aβ40, Aβ42/Aβ40, Aβ42/Aβ38, APOE4 | 0.908 | 0.020 |
| Aβ42, Aβ40, Aβ42/Aβ40, Aβ42/Aβ38, AGE | 0.903 | 0.015 |
| Aβ42, Aβ40, Aβ38, Aβ42/Aβ40, Aβ40/Aβ38, PTEDUCAT, APOE4 | 0.907 | 0.019 |
| Aβ42, Aβ40, PTEDUCAT | 0.900 | 0.012 |
| Aβ40, Aβ38, Aβ42/Aβ38, Aβ40/Aβ38, APOE4 | 0.911 | 0.023 |
| Aβ42, Aβ40, Aβ42/Aβ40, Aβ42/Aβ38, PTGENDER | 0.911 | 0.023 |
| Aβ42, Aβ42/Aβ40, Aβ40/Aβ38, AGE, PTEDUCAT, APOE4 | 0.913 | 0.025 |
| Aβ40, Aβ42/Aβ40, AGE | 0.904 | 0.017 |
| Aβ40, Aβ38, Aβ42/Aβ38, Aβ40/Aβ38, PTGENDER, APOE4 | 0.910 | 0.022 |
| Aβ40, Aβ38, Aβ42/Aβ38, Aβ40/Aβ38, AGE, APOE4 | 0.917 | 0.029 |
| Aβ42, Aβ42/Aβ40, Aβ42/Aβ38, AGE | 0.909 | 0.021 |
| Aβ42, Aβ42/Aβ40, AGE, PTGENDER, APOE4 | 0.910 | 0.023 |
| Aβ40, Aβ42/Aβ40, Aβ40/Aβ38, AGE, PTEDUCAT | 0.905 | 0.018 |
| Aβ40, Aβ42/Aβ40, Aβ40/Aβ38, PTGENDER, APOE4 | 0.901 | 0.014 |
| Aβ42, Aβ40, Aβ42/Aβ40, AGE, PTGENDER | 0.911 | 0.024 |
| Aβ40, Aβ42/Aβ40, Aβ42/Aβ38, Aβ40/Aβ38, AGE, PTEDUCAT | 0.896 | 0.008 |
| Aβ42, Aβ42/Aβ40, AGE, PTGENDER | 0.912 | 0.024 |
| Aβ42, Aβ40, PTEDUCAT, APOE4 | 0.900 | 0.012 |
| Aβ40, Aβ42/Aβ40, Aβ40/Aβ38, PTEDUCAT, PTGENDER, APOE4 | 0.904 | 0.017 |
| Aβ42, Aβ40, Aβ42/Aβ40, Aβ40/Aβ38, AGE, PTGENDER, APOE4 | 0.906 | 0.019 |
| Aβ42, Aβ40, Aβ38, Aβ40/Aβ38, PTGENDER, APOE4 | 0.893 | 0.006 |
| Aβ42, Aβ42/Aβ40, Aβ40/Aβ38, PTEDUCAT | 0.913 | 0.026 |
| Aβ42, Aβ42/Aβ40, PTEDUCAT, PTGENDER | 0.912 | 0.025 |
| Aβ40, Aβ42/Aβ38, Aβ40/Aβ38, AGE | 0.911 | 0.024 |
| Aβ42, Aβ40, Aβ38, Aβ42/Aβ40, Aβ42/Aβ38, Aβ40/Aβ38 | 0.910 | 0.024 |
| Aβ42, Aβ40, Aβ42/Aβ38, Aβ40/Aβ38, PTEDUCAT | 0.910 | 0.023 |
| Aβ42, Aβ42/Aβ40, PTGENDER | 0.911 | 0.025 |
| Aβ42, Aβ42/Aβ40, Aβ42/Aβ38, Aβ40/Aβ38, PTEDUCAT, APOE4 | 0.904 | 0.018 |
| Aβ40, Aβ38, Aβ42/Aβ38, Aβ40/Aβ38, AGE, PTGENDER, APOE4 | 0.914 | 0.027 |
| Aβ40, Aβ42/Aβ38, AGE, PTGENDER, APOE4 | 0.906 | 0.019 |
| Aβ40, Aβ42/Aβ40, PTGENDER | 0.909 | 0.022 |
| Aβ42, Aβ40, Aβ38, Aβ40/Aβ38 | 0.915 | 0.029 |
| Aβ42, Aβ40, Aβ42/Aβ40, PTGENDER | 0.909 | 0.022 |
| Aβ40, Aβ42/Aβ40, Aβ42/Aβ38, Aβ40/Aβ38, PTEDUCAT | 0.899 | 0.013 |
| Aβ42, Aβ40, Aβ42/Aβ40, AGE, PTEDUCAT, PTGENDER | 0.909 | 0.023 |
| Aβ42, Aβ42/Aβ40, Aβ42/Aβ38, Aβ40/Aβ38, AGE, PTEDUCAT, PTGENDER | 0.908 | 0.022 |
| Aβ40, Aβ38, Aβ42/Aβ40, Aβ40/Aβ38, PTGENDER | 0.908 | 0.022 |
| Aβ42, Aβ40, Aβ42/Aβ40, Aβ42/Aβ38, Aβ40/Aβ38, AGE, PTEDUCAT | 0.896 | 0.010 |
| Aβ42, Aβ40, Aβ42/Aβ40, Aβ40/Aβ38, AGE, PTEDUCAT | 0.906 | 0.020 |
| Aβ40, Aβ42/Aβ38, AGE, PTEDUCAT, PTGENDER, APOE4 | 0.914 | 0.028 |
| Aβ40, Aβ42/Aβ38, AGE, PTGENDER | 0.914 | 0.028 |
| Aβ42, Aβ40, Aβ42/Aβ40, Aβ42/Aβ38, AGE, PTEDUCAT | 0.906 | 0.021 |
| Aβ40, Aβ38, Aβ42/Aβ40, Aβ42/Aβ38, Aβ40/Aβ38, AGE | 0.915 | 0.030 |
| Aβ42, Aβ42/Aβ40, APOE4 | 0.909 | 0.023 |
| Aβ42, Aβ42/Aβ40, Aβ40/Aβ38, AGE, APOE4 | 0.912 | 0.026 |
| Aβ42, Aβ40, Aβ38, Aβ42/Aβ40, Aβ40/Aβ38, PTGENDER | 0.908 | 0.023 |
| Aβ42, Aβ40, Aβ42/Aβ40, Aβ42/Aβ38, Aβ40/Aβ38, PTEDUCAT | 0.900 | 0.015 |
| Aβ42, Aβ40, Aβ38, Aβ40/Aβ38, PTGENDER | 0.911 | 0.026 |
| Aβ42, Aβ38, Aβ42/Aβ40, Aβ40/Aβ38 | 0.907 | 0.022 |
| Aβ40, Aβ42/Aβ38, Aβ40/Aβ38, PTGENDER, APOE4 | 0.900 | 0.016 |
| Aβ42, Aβ42/Aβ40, Aβ40/Aβ38, AGE | 0.907 | 0.022 |
| Aβ42, Aβ40, Aβ38, Aβ42/Aβ40, Aβ40/Aβ38, AGE, PTGENDER | 0.909 | 0.025 |
| Aβ40, Aβ38, Aβ42/Aβ40, Aβ42/Aβ38, Aβ40/Aβ38, PTEDUCAT, PTGENDER | 0.909 | 0.025 |
| Aβ42, Aβ40, Aβ38, Aβ42/Aβ40, Aβ40/Aβ38, PTEDUCAT | 0.899 | 0.015 |
| Aβ42, Aβ40, Aβ42/Aβ40, Aβ42/Aβ38, Aβ40/Aβ38, AGE | 0.891 | 0.006 |
| Aβ42, Aβ40, Aβ42/Aβ38, Aβ40/Aβ38, PTGENDER | 0.895 | 0.011 |
| Aβ42, Aβ38, Aβ42/Aβ38, Aβ40/Aβ38 | 0.908 | 0.024 |
| Aβ42, Aβ40, Aβ40/Aβ38, AGE | 0.897 | 0.013 |
| Aβ42, Aβ42/Aβ40, Aβ40/Aβ38, AGE, PTEDUCAT | 0.910 | 0.026 |
| Aβ42, Aβ40, Aβ42/Aβ40, PTEDUCAT, PTGENDER | 0.909 | 0.025 |
| Aβ42, Aβ40, Aβ42/Aβ40, Aβ42/Aβ38, Aβ40/Aβ38, APOE4 | 0.895 | 0.012 |
| Aβ40, Aβ42/Aβ38, Aβ40/Aβ38, APOE4 | 0.907 | 0.023 |
| Aβ42, Aβ42/Aβ40, Aβ42/Aβ38, Aβ40/Aβ38, PTEDUCAT, PTGENDER | 0.905 | 0.021 |
| Aβ42, Aβ38, Aβ42/Aβ40, Aβ42/Aβ38, Aβ40/Aβ38, AGE | 0.895 | 0.012 |
| Aβ42, Aβ40, Aβ38, Aβ42/Aβ40 | 0.899 | 0.016 |
| Aβ42, Aβ42/Aβ40, PTEDUCAT, APOE4 | 0.911 | 0.028 |
| Aβ42, Aβ40, Aβ40/Aβ38, APOE4 | 0.895 | 0.012 |
| Aβ42, Aβ42/Aβ40, Aβ40/Aβ38 | 0.908 | 0.025 |
| Aβ42, Aβ40, Aβ38, Aβ42/Aβ40, Aβ40/Aβ38, AGE, PTEDUCAT | 0.900 | 0.017 |
| Aβ40, Aβ42/Aβ40, AGE, PTEDUCAT | 0.903 | 0.020 |
| Aβ42, Aβ38, Aβ42/Aβ40, Aβ42/Aβ38, Aβ40/Aβ38, AGE, PTEDUCAT | 0.895 | 0.013 |
| Aβ40, Aβ42/Aβ40, Aβ42/Aβ38 | 0.908 | 0.025 |
| Aβ40, Aβ38, Aβ42/Aβ40, Aβ42/Aβ38, Aβ40/Aβ38, AGE, PTEDUCAT | 0.913 | 0.030 |
| Aβ42, Aβ42/Aβ40, Aβ42/Aβ38, AGE, PTEDUCAT | 0.906 | 0.024 |
| Aβ40, Aβ42/Aβ40, PTEDUCAT | 0.903 | 0.020 |
| Aβ40, Aβ42/Aβ38, Aβ40/Aβ38, PTEDUCAT | 0.911 | 0.029 |
| Aβ42, Aβ40, Aβ42/Aβ40, Aβ40/Aβ38, AGE, PTGENDER | 0.908 | 0.025 |
| Aβ42, Aβ40, Aβ38, Aβ42/Aβ40, Aβ42/Aβ38, Aβ40/Aβ38, PTGENDER, APOE4 | 0.891 | 0.009 |
| Aβ40, Aβ42/Aβ38, Aβ40/Aβ38, PTGENDER | 0.905 | 0.022 |
| Aβ42, Aβ38, Aβ42/Aβ40, Aβ40/Aβ38, PTEDUCAT | 0.909 | 0.027 |
| Aβ42, Aβ42/Aβ40, PTGENDER, APOE4 | 0.910 | 0.027 |
| Aβ42, Aβ40, Aβ42/Aβ40, Aβ42/Aβ38, Aβ40/Aβ38, PTEDUCAT, APOE4 | 0.896 | 0.014 |
| Aβ40, Aβ42/Aβ40, Aβ42/Aβ38, AGE, PTEDUCAT | 0.901 | 0.019 |
| Aβ42, Aβ40, Aβ42/Aβ40, Aβ42/Aβ38, PTEDUCAT | 0.899 | 0.017 |
| Aβ40, Aβ42/Aβ40, Aβ42/Aβ38, AGE | 0.899 | 0.017 |
| Aβ42, Aβ40, Aβ42/Aβ40, Aβ42/Aβ38, AGE, PTGENDER | 0.901 | 0.019 |
| Aβ40, Aβ38, Aβ42/Aβ38, Aβ40/Aβ38 | 0.914 | 0.032 |
| Aβ40, Aβ42/Aβ40, Aβ40/Aβ38, PTEDUCAT, PTGENDER | 0.904 | 0.022 |
| Aβ40, Aβ42/Aβ40, AGE, PTGENDER | 0.911 | 0.029 |
| Aβ42, Aβ40, Aβ40/Aβ38, AGE, PTEDUCAT, PTGENDER | 0.888 | 0.006 |
| Aβ40, Aβ38, Aβ42/Aβ40, Aβ42/Aβ38, Aβ40/Aβ38 | 0.909 | 0.028 |
| Aβ42, Aβ42/Aβ40, Aβ42/Aβ38, Aβ40/Aβ38, AGE, PTEDUCAT, APOE4 | 0.905 | 0.023 |
| Aβ42, Aβ42/Aβ40, PTEDUCAT, PTGENDER, APOE4 | 0.909 | 0.027 |
| Aβ42, Aβ38, Aβ42/Aβ40, Aβ42/Aβ38, Aβ40/Aβ38, PTEDUCAT | 0.896 | 0.014 |
| Aβ40, Aβ42/Aβ40, PTEDUCAT, PTGENDER | 0.905 | 0.024 |
| Aβ42, Aβ40, Aβ42/Aβ40, PTEDUCAT, APOE4 | 0.906 | 0.024 |
| Aβ42, Aβ38, Aβ42/Aβ40, Aβ40/Aβ38, AGE | 0.903 | 0.021 |
| Aβ40, Aβ42/Aβ38, Aβ40/Aβ38, PTEDUCAT, PTGENDER | 0.914 | 0.033 |
| Aβ42, Aβ42/Aβ40, Aβ42/Aβ38, PTEDUCAT | 0.906 | 0.025 |
| Aβ40, Aβ38, Aβ42/Aβ40 | 0.908 | 0.027 |
| Aβ40, Aβ42/Aβ40, Aβ42/Aβ38, PTEDUCAT, PTGENDER | 0.913 | 0.032 |
| Aβ40, Aβ38, Aβ42/Aβ40, Aβ40/Aβ38, AGE, PTGENDER, APOE4 | 0.910 | 0.029 |
| Aβ42, Aβ42/Aβ40, Aβ42/Aβ38, Aβ40/Aβ38, AGE | 0.904 | 0.023 |
| Aβ42, Aβ40, Aβ42/Aβ40, Aβ40/Aβ38, AGE | 0.899 | 0.017 |
| Aβ42, Aβ40, Aβ42/Aβ38, Aβ40/Aβ38, APOE4 | 0.900 | 0.019 |
| Aβ42, Aβ40, Aβ42/Aβ40, Aβ40/Aβ38, APOE4 | 0.898 | 0.017 |
| Aβ42, Aβ42/Aβ40, Aβ40/Aβ38, APOE4 | 0.907 | 0.026 |
| Aβ42, Aβ40, Aβ42/Aβ40, APOE4 | 0.903 | 0.022 |
| Aβ42, Aβ40, Aβ38, Aβ42/Aβ40, Aβ40/Aβ38, AGE, APOE4 | 0.907 | 0.026 |
| Aβ40, Aβ42/Aβ40, Aβ42/Aβ38, Aβ40/Aβ38, AGE, PTEDUCAT, PTGENDER | 0.894 | 0.013 |
| Aβ42, Aβ40, Aβ38, Aβ42/Aβ40, AGE | 0.900 | 0.019 |
| Aβ42, Aβ40, Aβ42/Aβ38, APOE4 | 0.905 | 0.024 |
| Aβ42, Aβ40, Aβ38, Aβ42/Aβ38, Aβ40/Aβ38, APOE4 | 0.902 | 0.021 |
| Aβ42, Aβ40, Aβ42/Aβ40, AGE, APOE4 | 0.903 | 0.022 |
| Aβ42, Aβ42/Aβ40, AGE, PTEDUCAT, PTGENDER | 0.910 | 0.029 |
| Aβ42, Aβ42/Aβ40, Aβ40/Aβ38, PTEDUCAT, APOE4 | 0.908 | 0.027 |
| Aβ40, Aβ42/Aβ40, AGE, PTEDUCAT, PTGENDER | 0.903 | 0.022 |
| Aβ40, Aβ38, Aβ42/Aβ40, Aβ42/Aβ38 | 0.912 | 0.031 |
| Aβ40, Aβ42/Aβ38, Aβ40/Aβ38, PTEDUCAT, PTGENDER, APOE4 | 0.907 | 0.026 |
| Aβ40, Aβ42/Aβ40, Aβ42/Aβ38, PTEDUCAT, APOE4 | 0.892 | 0.012 |
| Aβ40, Aβ38, Aβ42/Aβ40, AGE | 0.910 | 0.029 |
| Aβ42, Aβ40, Aβ42/Aβ40, PTGENDER, APOE4 | 0.901 | 0.021 |
| Aβ42, Aβ40, Aβ42/Aβ40, Aβ42/Aβ38, Aβ40/Aβ38, PTGENDER | 0.892 | 0.012 |
| Aβ42, Aβ42/Aβ40, AGE, PTEDUCAT, APOE4 | 0.910 | 0.029 |
| Aβ42, Aβ42/Aβ40, Aβ42/Aβ38, Aβ40/Aβ38 | 0.902 | 0.022 |
| Aβ42, Aβ42/Aβ40, Aβ42/Aβ38, AGE, APOE4 | 0.900 | 0.020 |
| Aβ42, Aβ38, Aβ42/Aβ40, Aβ40/Aβ38, AGE, PTEDUCAT | 0.903 | 0.023 |
| Aβ40, Aβ42/Aβ40, Aβ42/Aβ38, Aβ40/Aβ38, PTEDUCAT, PTGENDER | 0.891 | 0.011 |
| Aβ42, Aβ38, Aβ42/Aβ40, Aβ42/Aβ38, Aβ40/Aβ38, AGE, PTGENDER | 0.895 | 0.015 |
| Aβ42, Aβ42/Aβ40, Aβ42/Aβ38, Aβ40/Aβ38, PTEDUCAT, PTGENDER, APOE4 | 0.902 | 0.022 |
| Aβ40, Aβ38, Aβ42/Aβ40, Aβ40/Aβ38, AGE | 0.907 | 0.027 |
| Aβ42, Aβ38, Aβ42/Aβ40, Aβ40/Aβ38, PTGENDER | 0.908 | 0.028 |
| Aβ40, Aβ42/Aβ40, Aβ40/Aβ38, AGE | 0.898 | 0.018 |
| Aβ42, Aβ40, PTEDUCAT, PTGENDER | 0.892 | 0.012 |
| Aβ42, Aβ42/Aβ40, Aβ42/Aβ38, AGE, PTGENDER | 0.908 | 0.028 |
| Aβ42, Aβ40, Aβ42/Aβ40, Aβ40/Aβ38, PTEDUCAT, PTGENDER | 0.901 | 0.022 |
| Aβ40, Aβ42/Aβ40, Aβ42/Aβ38, APOE4 | 0.904 | 0.025 |
| Aβ42, Aβ40, Aβ38, Aβ42/Aβ40, Aβ40/Aβ38, AGE, PTEDUCAT, APOE4 | 0.900 | 0.020 |
| Aβ40, Aβ42/Aβ40, Aβ42/Aβ38, Aβ40/Aβ38, PTGENDER | 0.898 | 0.018 |
| Aβ40, Aβ42/Aβ38, AGE, PTEDUCAT | 0.907 | 0.028 |
| Aβ40, Aβ38, Aβ42/Aβ40, Aβ42/Aβ38, AGE, PTEDUCAT | 0.917 | 0.038 |
| Aβ42, Aβ38, Aβ40/Aβ38, PTEDUCAT | 0.895 | 0.015 |
| Aβ42, Aβ40, Aβ38, Aβ42/Aβ40, AGE, PTGENDER | 0.901 | 0.021 |
| Aβ42, Aβ42/Aβ40, Aβ42/Aβ38, Aβ40/Aβ38, APOE4 | 0.900 | 0.021 |
| Aβ42, Aβ42/Aβ40, AGE, PTEDUCAT, PTGENDER, APOE4 | 0.908 | 0.029 |
| Aβ40, Aβ42/Aβ40, Aβ42/Aβ38, PTGENDER | 0.905 | 0.026 |
| Aβ42, Aβ40, Aβ38, Aβ42/Aβ40, Aβ40/Aβ38, AGE, PTEDUCAT, PTGENDER, APOE4 | 0.910 | 0.030 |
| Aβ42, Aβ42/Aβ40, Aβ42/Aβ38 | 0.903 | 0.024 |
| Aβ40, Aβ38, Aβ42/Aβ40, Aβ40/Aβ38, PTEDUCAT, PTGENDER, APOE4 | 0.908 | 0.029 |
| Aβ42, Aβ40, Aβ38, Aβ42/Aβ40, PTEDUCAT | 0.900 | 0.021 |
| Aβ42, Aβ40, Aβ38, Aβ42/Aβ40, APOE4 | 0.900 | 0.021 |
| Aβ42, Aβ38, Aβ42/Aβ40, Aβ42/Aβ38, Aβ40/Aβ38, PTEDUCAT, PTGENDER | 0.897 | 0.019 |
| Aβ42, Aβ40, AGE, PTEDUCAT | 0.900 | 0.021 |
| Aβ42, Aβ40 | 0.892 | 0.013 |
| Aβ42, Aβ38, Aβ42/Aβ40, Aβ42/Aβ38, Aβ40/Aβ38, APOE4 | 0.896 | 0.017 |
| Aβ42, Aβ40, Aβ40/Aβ38, PTEDUCAT | 0.905 | 0.026 |
| Aβ42, Aβ40, Aβ42/Aβ40, Aβ42/Aβ38, Aβ40/Aβ38, AGE, PTEDUCAT, PTGENDER | 0.892 | 0.014 |
| Aβ42, Aβ40, Aβ42/Aβ40, Aβ42/Aβ38, Aβ40/Aβ38, PTEDUCAT, PTGENDER | 0.891 | 0.012 |
| Aβ42, Aβ40, Aβ42/Aβ40, PTEDUCAT, PTGENDER, APOE4 | 0.903 | 0.024 |
| Aβ40, Aβ42/Aβ40, APOE4 | 0.898 | 0.019 |
| Aβ42, Aβ40, Aβ40/Aβ38, PTGENDER, APOE4 | 0.891 | 0.013 |
| Aβ42, Aβ40, Aβ40/Aβ38, PTEDUCAT, PTGENDER | 0.900 | 0.021 |
| Aβ42, Aβ40, AGE, PTGENDER, APOE4 | 0.895 | 0.016 |
| Aβ42, Aβ38, Aβ40/Aβ38 | 0.894 | 0.015 |
| Aβ40, Aβ38, Aβ42/Aβ40, Aβ40/Aβ38, AGE, PTEDUCAT, PTGENDER | 0.908 | 0.029 |
| Aβ42, Aβ38, Aβ42/Aβ40, Aβ42/Aβ38, Aβ40/Aβ38, PTEDUCAT, APOE4 | 0.895 | 0.017 |
| Aβ38, Aβ42/Aβ40, Aβ42/Aβ38, Aβ40/Aβ38, AGE, PTEDUCAT | 0.894 | 0.016 |
| Aβ42, Aβ40, Aβ38, Aβ42/Aβ40, Aβ42/Aβ38, AGE | 0.898 | 0.020 |
| Aβ42, Aβ40, Aβ38, Aβ42/Aβ40, Aβ42/Aβ38, Aβ40/Aβ38, PTEDUCAT, APOE4 | 0.897 | 0.019 |
| Aβ42, Aβ40, Aβ38, Aβ42/Aβ40, Aβ40/Aβ38, AGE, PTEDUCAT, PTGENDER | 0.903 | 0.025 |
| Aβ42, Aβ42/Aβ40, Aβ40/Aβ38, PTGENDER | 0.907 | 0.028 |
| Aβ42, Aβ42/Aβ40, Aβ42/Aβ38, AGE, PTGENDER, APOE4 | 0.904 | 0.026 |
| Aβ42, Aβ38, Aβ42/Aβ40, Aβ40/Aβ38, PTEDUCAT, APOE4 | 0.901 | 0.023 |
| Aβ38, Aβ42/Aβ40, Aβ40/Aβ38 | 0.905 | 0.027 |
| Aβ42, Aβ40, AGE, PTEDUCAT, APOE4 | 0.899 | 0.021 |
| Aβ42, Aβ40, Aβ38, Aβ42/Aβ40, Aβ40/Aβ38, PTEDUCAT, PTGENDER | 0.901 | 0.023 |
| Aβ40, Aβ38, Aβ42/Aβ40, Aβ40/Aβ38, AGE, PTGENDER | 0.909 | 0.031 |
| Aβ42, Aβ40, Aβ38, Aβ40/Aβ38, AGE, PTEDUCAT, PTGENDER, APOE4 | 0.901 | 0.023 |
| Aβ42, Aβ40, Aβ42/Aβ40, AGE, PTEDUCAT, APOE4 | 0.904 | 0.026 |
| Aβ42, Aβ40, Aβ38, Aβ42/Aβ38, Aβ40/Aβ38, PTEDUCAT | 0.902 | 0.024 |
| Aβ42, Aβ42/Aβ40, Aβ42/Aβ38, PTGENDER | 0.901 | 0.023 |
| Aβ42, Aβ40, Aβ38, Aβ42/Aβ38, Aβ40/Aβ38, PTGENDER | 0.907 | 0.029 |
| Aβ40, Aβ42/Aβ40, Aβ42/Aβ38, Aβ40/Aβ38, AGE | 0.909 | 0.031 |
| Aβ40, Aβ38, Aβ42/Aβ38, Aβ40/Aβ38, PTEDUCAT | 0.902 | 0.024 |
| Aβ40, Aβ42/Aβ40, Aβ42/Aβ38, Aβ40/Aβ38, PTEDUCAT, APOE4 | 0.892 | 0.015 |
| Aβ40, Aβ38, Aβ42/Aβ40, Aβ40/Aβ38, PTGENDER, APOE4 | 0.899 | 0.021 |
| Aβ40, Aβ38, Aβ42/Aβ40, Aβ40/Aβ38, AGE, PTEDUCAT, APOE4 | 0.907 | 0.030 |
| Aβ42, Aβ40, Aβ42/Aβ40, Aβ42/Aβ38, AGE, APOE4 | 0.906 | 0.029 |
| Aβ40, Aβ42/Aβ40, AGE, APOE4 | 0.900 | 0.023 |
| Aβ40, Aβ38, Aβ42/Aβ40, Aβ40/Aβ38, PTEDUCAT, APOE4 | 0.900 | 0.022 |
| Aβ40, Aβ38, Aβ42/Aβ40, Aβ42/Aβ38, AGE, PTEDUCAT, PTGENDER, APOE4 | 0.914 | 0.037 |
| Aβ40, Aβ42/Aβ40, Aβ42/Aβ38, Aβ40/Aβ38 | 0.900 | 0.023 |
| Aβ42, Aβ40, Aβ42/Aβ40, Aβ42/Aβ38, PTEDUCAT, PTGENDER | 0.895 | 0.018 |
| Aβ42, Aβ38, Aβ42/Aβ40, Aβ40/Aβ38, AGE, APOE4 | 0.905 | 0.028 |
| Aβ42, Aβ42/Aβ40, Aβ42/Aβ38, PTEDUCAT, APOE4 | 0.902 | 0.025 |
| Aβ42, Aβ38, Aβ40/Aβ38, PTGENDER, APOE4 | 0.891 | 0.014 |
| Aβ42, Aβ40, Aβ42/Aβ40, Aβ42/Aβ38, Aβ40/Aβ38, PTEDUCAT, PTGENDER, APOE4 | 0.902 | 0.025 |
| Aβ42, Aβ40, Aβ38, Aβ42/Aβ38, Aβ40/Aβ38, PTGENDER, APOE4 | 0.902 | 0.025 |
| Aβ40, Aβ42/Aβ38, Aβ40/Aβ38, AGE, PTEDUCAT, PTGENDER, APOE4 | 0.911 | 0.034 |
| Aβ42, Aβ40, Aβ42/Aβ40, Aβ42/Aβ38, Aβ40/Aβ38, AGE, PTGENDER | 0.887 | 0.010 |
| Aβ42, Aβ40, Aβ42/Aβ38, Aβ40/Aβ38, AGE | 0.889 | 0.012 |
| Aβ40, Aβ42/Aβ40, Aβ42/Aβ38, AGE, PTGENDER | 0.898 | 0.021 |
| Aβ42, Aβ40, Aβ38, Aβ42/Aβ40, Aβ42/Aβ38, Aβ40/Aβ38, AGE, PTEDUCAT, PTGENDER, APOE4 | 0.895 | 0.018 |
| Aβ40, Aβ42/Aβ40, Aβ42/Aβ38, Aβ40/Aβ38, PTGENDER, APOE4 | 0.903 | 0.026 |
| Aβ40, Aβ38, Aβ42/Aβ40, Aβ40/Aβ38, APOE4 | 0.898 | 0.021 |
| Aβ40, Aβ38, Aβ42/Aβ40, Aβ42/Aβ38, Aβ40/Aβ38, PTGENDER, APOE4 | 0.903 | 0.027 |
| Aβ42, Aβ40, Aβ42/Aβ40, Aβ42/Aβ38, PTEDUCAT, APOE4 | 0.901 | 0.024 |
| Aβ40, Aβ38, Aβ42/Aβ40, Aβ42/Aβ38, Aβ40/Aβ38, AGE, PTGENDER, APOE4 | 0.909 | 0.032 |
| Aβ42, Aβ40, Aβ38, Aβ40/Aβ38, AGE, PTEDUCAT, PTGENDER | 0.890 | 0.013 |
| Aβ42, Aβ40, AGE | 0.897 | 0.020 |
| Aβ40, Aβ42/Aβ40, Aβ42/Aβ38, Aβ40/Aβ38, AGE, PTEDUCAT, APOE4 | 0.903 | 0.027 |
| Aβ42, Aβ42/Aβ40, Aβ40/Aβ38, AGE, PTGENDER | 0.906 | 0.029 |
| Aβ40, Aβ42/Aβ38, Aβ40/Aβ38, AGE, APOE4 | 0.907 | 0.031 |
| Aβ42, Aβ40, Aβ38, Aβ42/Aβ40, Aβ42/Aβ38, Aβ40/Aβ38, PTEDUCAT | 0.902 | 0.026 |
| Aβ40, Aβ38, Aβ42/Aβ40, Aβ42/Aβ38, PTEDUCAT, APOE4 | 0.907 | 0.030 |
| Aβ42, Aβ38, Aβ42/Aβ40, AGE | 0.902 | 0.026 |
| Aβ42, Aβ42/Aβ40, Aβ40/Aβ38, PTEDUCAT, PTGENDER | 0.906 | 0.029 |
| Aβ42, Aβ42/Aβ40, Aβ42/Aβ38, Aβ40/Aβ38, AGE, PTGENDER | 0.898 | 0.022 |
| Aβ42, Aβ40, Aβ42/Aβ38, AGE, PTEDUCAT | 0.903 | 0.027 |
| Aβ40, Aβ38, Aβ42/Aβ40, Aβ42/Aβ38, AGE, APOE4 | 0.912 | 0.035 |
| Aβ42, Aβ40, Aβ42/Aβ40, Aβ40/Aβ38, PTEDUCAT, PTGENDER, APOE4 | 0.901 | 0.025 |
| Aβ42, Aβ42/Aβ40, Aβ42/Aβ38, AGE, PTEDUCAT, APOE4 | 0.904 | 0.028 |
| Aβ40, Aβ38, Aβ42/Aβ40, Aβ42/Aβ38, PTEDUCAT, PTGENDER | 0.910 | 0.034 |
| Aβ42, Aβ42/Aβ40, Aβ42/Aβ38, Aβ40/Aβ38, PTGENDER | 0.900 | 0.024 |
| Aβ42, Aβ40, Aβ40/Aβ38, PTEDUCAT, APOE4 | 0.892 | 0.016 |
| Aβ40, Aβ38, Aβ42/Aβ40, PTEDUCAT | 0.907 | 0.031 |
| Aβ42, Aβ40, Aβ42/Aβ40, Aβ42/Aβ38, Aβ40/Aβ38, AGE, PTEDUCAT, APOE4 | 0.889 | 0.013 |
| Aβ40, Aβ42/Aβ40, PTEDUCAT, APOE4 | 0.899 | 0.023 |
| Aβ42, Aβ38, Aβ42/Aβ40, Aβ42/Aβ38, AGE, PTGENDER | 0.902 | 0.026 |
| Aβ40, Aβ42/Aβ40, Aβ42/Aβ38, AGE, PTEDUCAT, APOE4 | 0.902 | 0.026 |
| Aβ42, Aβ42/Aβ40, Aβ40/Aβ38, PTGENDER, APOE4 | 0.905 | 0.029 |
| Aβ42, Aβ42/Aβ40, Aβ42/Aβ38, Aβ40/Aβ38, AGE, PTEDUCAT, PTGENDER, APOE4 | 0.902 | 0.026 |
| Aβ40, Aβ42/Aβ38, AGE | 0.902 | 0.026 |
| Aβ42, Aβ40, Aβ38, Aβ42/Aβ38, Aβ40/Aβ38, PTEDUCAT, APOE4 | 0.898 | 0.022 |
| Aβ40, Aβ42/Aβ38, PTEDUCAT, APOE4 | 0.904 | 0.028 |
| Aβ42, Aβ40, Aβ38, Aβ40/Aβ38, PTEDUCAT | 0.903 | 0.028 |
| Aβ42, Aβ38, Aβ42/Aβ38, Aβ40/Aβ38, AGE | 0.887 | 0.011 |
| Aβ42, Aβ42/Aβ40, Aβ40/Aβ38, PTEDUCAT, PTGENDER, APOE4 | 0.904 | 0.028 |
| Aβ42, Aβ40, Aβ42/Aβ40, AGE, PTGENDER, APOE4 | 0.901 | 0.025 |
| Aβ38, Aβ42/Aβ40, Aβ40/Aβ38, PTGENDER, APOE4 | 0.899 | 0.023 |
| Aβ42, Aβ40, Aβ42/Aβ40, Aβ40/Aβ38, AGE, PTEDUCAT, PTGENDER | 0.904 | 0.029 |
| Aβ42, Aβ38, Aβ42/Aβ38, Aβ40/Aβ38, AGE, PTEDUCAT | 0.899 | 0.023 |
| Aβ42, Aβ40, Aβ38, Aβ42/Aβ38, Aβ40/Aβ38, PTEDUCAT, PTGENDER, APOE4 | 0.903 | 0.028 |
| Aβ42, Aβ40, Aβ42/Aβ38, Aβ40/Aβ38, PTEDUCAT, PTGENDER | 0.903 | 0.027 |
| Aβ40, Aβ42/Aβ38, PTEDUCAT | 0.905 | 0.030 |
| Aβ42, Aβ38, Aβ42/Aβ40, Aβ40/Aβ38, APOE4 | 0.902 | 0.026 |
| Aβ42, Aβ40, Aβ42/Aβ38, Aβ40/Aβ38, AGE, PTGENDER | 0.906 | 0.030 |
| Aβ40, Aβ38, Aβ42/Aβ38, Aβ40/Aβ38, AGE, PTEDUCAT | 0.911 | 0.035 |
| Aβ40, Aβ38, Aβ42/Aβ40, Aβ42/Aβ38, Aβ40/Aβ38, PTEDUCAT, PTGENDER, APOE4 | 0.903 | 0.027 |
| Aβ42, Aβ40, Aβ42/Aβ40, Aβ42/Aβ38, PTGENDER, APOE4 | 0.905 | 0.029 |
| Aβ42, Aβ40, Aβ38, Aβ42/Aβ40, Aβ42/Aβ38, AGE, PTEDUCAT | 0.913 | 0.038 |
| Aβ40, Aβ38, Aβ42/Aβ38, Aβ40/Aβ38, PTEDUCAT, PTGENDER, APOE4 | 0.906 | 0.030 |
| Aβ42, Aβ38, Aβ40/Aβ38, AGE | 0.900 | 0.024 |
| Aβ40, Aβ38, Aβ42/Aβ40, APOE4 | 0.906 | 0.031 |
| Aβ40, Aβ42/Aβ40, Aβ42/Aβ38, AGE, APOE4 | 0.898 | 0.023 |
| Aβ40, Aβ42/Aβ40, Aβ40/Aβ38, AGE, PTEDUCAT, PTGENDER | 0.898 | 0.023 |
| Aβ42, Aβ40, Aβ42/Aβ40, Aβ42/Aβ38, Aβ40/Aβ38, AGE, APOE4 | 0.883 | 0.008 |
| Aβ40, Aβ38, Aβ42/Aβ40, Aβ40/Aβ38, AGE, APOE4 | 0.903 | 0.028 |
| Aβ40, Aβ42/Aβ40, Aβ40/Aβ38, AGE, PTEDUCAT, APOE4 | 0.897 | 0.022 |
| Aβ40, Aβ38, Aβ42/Aβ40, Aβ42/Aβ38, Aβ40/Aβ38, AGE, PTEDUCAT, PTGENDER | 0.908 | 0.033 |
| Aβ42, Aβ38, Aβ42/Aβ40, Aβ42/Aβ38, AGE | 0.898 | 0.023 |
| Aβ40, Aβ42/Aβ40, Aβ40/Aβ38, AGE, APOE4 | 0.904 | 0.029 |
| Aβ40, Aβ38, Aβ42/Aβ40, Aβ42/Aβ38, Aβ40/Aβ38, AGE, APOE4 | 0.897 | 0.023 |
| Aβ42, Aβ40, Aβ42/Aβ38, Aβ40/Aβ38, AGE, PTEDUCAT | 0.898 | 0.024 |
| Aβ42, Aβ38, Aβ42/Aβ40, AGE, PTEDUCAT | 0.905 | 0.030 |
| Aβ42, Aβ40, Aβ38, Aβ42/Aβ40, Aβ42/Aβ38, Aβ40/Aβ38, AGE, PTGENDER | 0.889 | 0.015 |
| Aβ40, Aβ38, Aβ42/Aβ40, PTGENDER | 0.895 | 0.020 |
| Aβ40, Aβ42/Aβ38, Aβ40/Aβ38, PTEDUCAT, APOE4 | 0.900 | 0.026 |
| Aβ42, Aβ40, Aβ42/Aβ38 | 0.893 | 0.019 |
| Aβ40, Aβ38, Aβ42/Aβ40, Aβ42/Aβ38, Aβ40/Aβ38, AGE, PTGENDER | 0.901 | 0.027 |
| Aβ42, Aβ40, Aβ38, Aβ42/Aβ40, Aβ42/Aβ38, Aβ40/Aβ38, PTGENDER | 0.887 | 0.012 |
| Aβ42, Aβ40, Aβ38, Aβ42/Aβ40, PTGENDER | 0.896 | 0.022 |
| Aβ40, Aβ42/Aβ40, Aβ42/Aβ38, Aβ40/Aβ38, AGE, APOE4 | 0.900 | 0.025 |
| Aβ40, Aβ42/Aβ40, AGE, PTEDUCAT, PTGENDER, APOE4 | 0.903 | 0.029 |
| Aβ40, Aβ42/Aβ40, Aβ42/Aβ38, AGE, PTGENDER, APOE4 | 0.897 | 0.022 |
| Aβ38, Aβ42/Aβ40, Aβ42/Aβ38, Aβ40/Aβ38, PTEDUCAT | 0.886 | 0.012 |
| Aβ40, Aβ42/Aβ40, Aβ42/Aβ38, PTGENDER, APOE4 | 0.897 | 0.022 |
| Aβ42, Aβ40, Aβ38, Aβ42/Aβ40, Aβ40/Aβ38, PTEDUCAT, PTGENDER, APOE4 | 0.899 | 0.025 |
| Aβ40, Aβ42/Aβ40, Aβ42/Aβ38, Aβ40/Aβ38, AGE, PTEDUCAT, PTGENDER, APOE4 | 0.903 | 0.029 |
| Aβ40, Aβ38, Aβ42/Aβ38, Aβ40/Aβ38, PTEDUCAT, APOE4 | 0.903 | 0.029 |
| Aβ42, Aβ38, Aβ42/Aβ40, Aβ42/Aβ38, Aβ40/Aβ38, AGE, APOE4 | 0.891 | 0.017 |
| Aβ42, Aβ42/Aβ40, Aβ40/Aβ38, AGE, PTEDUCAT, PTGENDER | 0.905 | 0.031 |
| Aβ42, Aβ40, Aβ38, Aβ42/Aβ40, AGE, PTEDUCAT | 0.904 | 0.030 |
| Aβ42, Aβ40, Aβ42/Aβ40, Aβ42/Aβ38, AGE, PTEDUCAT, APOE4 | 0.903 | 0.029 |
| Aβ42, Aβ38, Aβ42/Aβ40, Aβ40/Aβ38, AGE, PTGENDER | 0.895 | 0.021 |
| Aβ42, Aβ40, Aβ42/Aβ40, Aβ42/Aβ38, AGE, PTEDUCAT, PTGENDER | 0.900 | 0.026 |
| Aβ40, Aβ42/Aβ40, AGE, PTEDUCAT, APOE4 | 0.898 | 0.025 |
| Aβ42, Aβ40, Aβ42/Aβ40, Aβ40/Aβ38, AGE, PTEDUCAT, APOE4 | 0.899 | 0.026 |
| Aβ40, Aβ42/Aβ38, AGE, APOE4 | 0.896 | 0.022 |
| Aβ42, Aβ38, Aβ42/Aβ40, Aβ40/Aβ38, AGE, PTEDUCAT, PTGENDER | 0.901 | 0.027 |
| Aβ42, Aβ40, Aβ38, Aβ42/Aβ40, Aβ42/Aβ38, Aβ40/Aβ38, PTEDUCAT, PTGENDER, APOE4 | 0.904 | 0.030 |
| Aβ42, Aβ40, Aβ42/Aβ40, AGE, PTEDUCAT, PTGENDER, APOE4 | 0.902 | 0.028 |
| Aβ40, Aβ38, Aβ42/Aβ40, AGE, PTGENDER | 0.902 | 0.028 |
| Aβ40, Aβ42/Aβ40, Aβ42/Aβ38, AGE, PTEDUCAT, PTGENDER | 0.897 | 0.023 |
| Aβ42, Aβ40, Aβ42/Aβ38, AGE, PTEDUCAT, APOE4 | 0.886 | 0.013 |
| Aβ38, Aβ42/Aβ40, Aβ42/Aβ38, Aβ40/Aβ38 | 0.888 | 0.014 |
| Aβ42, Aβ40, PTGENDER | 0.893 | 0.020 |
| Aβ40, Aβ38, Aβ42/Aβ40, Aβ40/Aβ38, AGE, PTEDUCAT, PTGENDER, APOE4 | 0.898 | 0.024 |
| Aβ42, Aβ40, Aβ38, Aβ42/Aβ40, Aβ40/Aβ38, PTGENDER, APOE4 | 0.894 | 0.020 |
| Aβ42, Aβ38, Aβ42/Aβ40, Aβ40/Aβ38, AGE, PTEDUCAT, APOE4 | 0.903 | 0.030 |
| Aβ42, Aβ38, Aβ42/Aβ40, Aβ40/Aβ38, PTGENDER, APOE4 | 0.904 | 0.030 |
| Aβ42, Aβ38, Aβ42/Aβ40, Aβ40/Aβ38, PTEDUCAT, PTGENDER | 0.904 | 0.031 |
| Aβ42, Aβ40, Aβ42/Aβ38, Aβ40/Aβ38, AGE, PTEDUCAT, PTGENDER, APOE4 | 0.899 | 0.025 |
| Aβ42, Aβ40, Aβ38, Aβ42/Aβ40, Aβ42/Aβ38, Aβ40/Aβ38, APOE4 | 0.901 | 0.028 |
| Aβ38, Aβ42/Aβ40, Aβ42/Aβ38, PTEDUCAT, PTGENDER | 0.890 | 0.016 |
| Aβ42, Aβ40, Aβ38, Aβ42/Aβ40, AGE, PTGENDER, APOE4 | 0.905 | 0.031 |
| Aβ42, Aβ42/Aβ40, Aβ42/Aβ38, PTEDUCAT, PTGENDER | 0.901 | 0.028 |
| Aβ40, Aβ42/Aβ38, Aβ40/Aβ38, AGE, PTEDUCAT | 0.897 | 0.023 |
| Aβ42, Aβ42/Aβ40, Aβ42/Aβ38, PTGENDER, APOE4 | 0.905 | 0.032 |
| Aβ42, Aβ42/Aβ40, Aβ42/Aβ38, APOE4 | 0.903 | 0.030 |
| Aβ42, Aβ40, Aβ38, Aβ42/Aβ40, AGE, PTEDUCAT, APOE4 | 0.909 | 0.036 |
| Aβ42, Aβ40, Aβ40/Aβ38, AGE, PTGENDER, APOE4 | 0.895 | 0.022 |
| Aβ42, Aβ42/Aβ40, Aβ42/Aβ38, Aβ40/Aβ38, PTGENDER, APOE4 | 0.897 | 0.025 |
| Aβ40, Aβ42/Aβ40, Aβ40/Aβ38, AGE, PTGENDER | 0.894 | 0.021 |
| Aβ42, Aβ38, Aβ42/Aβ40, Aβ42/Aβ38, Aβ40/Aβ38, AGE, PTEDUCAT, APOE4 | 0.896 | 0.024 |
| Aβ40, Aβ42/Aβ40, Aβ42/Aβ38, Aβ40/Aβ38, AGE, PTGENDER, APOE4 | 0.900 | 0.027 |
| Aβ42, Aβ42/Aβ40, Aβ42/Aβ38, AGE, PTEDUCAT, PTGENDER | 0.901 | 0.028 |
| Aβ42, Aβ40, Aβ38, Aβ42/Aβ40, Aβ42/Aβ38, Aβ40/Aβ38, AGE, PTEDUCAT | 0.891 | 0.018 |
| Aβ42, Aβ40, Aβ38, Aβ40/Aβ38, PTEDUCAT, PTGENDER, APOE4 | 0.886 | 0.013 |
| Aβ40, Aβ42/Aβ40, Aβ42/Aβ38, PTEDUCAT, PTGENDER, APOE4 | 0.897 | 0.025 |
| Aβ40, Aβ42/Aβ40, PTEDUCAT, PTGENDER, APOE4 | 0.896 | 0.023 |
| Aβ42, Aβ42/Aβ40, Aβ42/Aβ38, AGE, PTEDUCAT, PTGENDER, APOE4 | 0.900 | 0.028 |
| Aβ38, Aβ42/Aβ40, Aβ40/Aβ38, AGE | 0.897 | 0.025 |
| Aβ40, Aβ38, Aβ42/Aβ40, PTGENDER, APOE4 | 0.901 | 0.028 |
| Aβ42, Aβ42/Aβ40, Aβ42/Aβ38, Aβ40/Aβ38, AGE, APOE4 | 0.898 | 0.026 |
| Aβ42, Aβ38, Aβ42/Aβ40, AGE, APOE4 | 0.903 | 0.030 |
| Aβ40, Aβ38, Aβ42/Aβ40, Aβ40/Aβ38, AGE, PTEDUCAT | 0.894 | 0.021 |
| Aβ42, Aβ38, Aβ42/Aβ40, Aβ42/Aβ38, Aβ40/Aβ38, PTGENDER, APOE4 | 0.899 | 0.027 |
| Aβ42, Aβ38, Aβ42/Aβ40, Aβ42/Aβ38, PTEDUCAT | 0.892 | 0.020 |
| Aβ42, Aβ40, Aβ38, Aβ42/Aβ40, Aβ42/Aβ38, Aβ40/Aβ38, PTEDUCAT, PTGENDER | 0.906 | 0.034 |
| Aβ42, Aβ38, Aβ42/Aβ40, Aβ42/Aβ38, Aβ40/Aβ38, PTGENDER | 0.898 | 0.026 |
| Aβ40, Aβ38, Aβ42/Aβ40, Aβ42/Aβ38, AGE | 0.893 | 0.021 |
| Aβ42, Aβ40, Aβ38, Aβ42/Aβ40, Aβ42/Aβ38, Aβ40/Aβ38, AGE, PTEDUCAT, PTGENDER | 0.911 | 0.039 |
| Aβ38, Aβ42/Aβ40, Aβ42/Aβ38, Aβ40/Aβ38, AGE | 0.890 | 0.018 |
| Aβ42, Aβ40, Aβ38, Aβ42/Aβ40, Aβ42/Aβ38, AGE, PTGENDER | 0.906 | 0.034 |
| Aβ40, Aβ42/Aβ40, AGE, PTGENDER, APOE4 | 0.897 | 0.025 |
| Aβ42, Aβ40, Aβ38, Aβ42/Aβ40, Aβ40/Aβ38, AGE, PTGENDER, APOE4 | 0.900 | 0.028 |
| Aβ42, Aβ40, Aβ42/Aβ38, AGE, APOE4 | 0.902 | 0.031 |
| Aβ42, Aβ40, Aβ38, Aβ42/Aβ40, Aβ42/Aβ38 | 0.894 | 0.022 |
| Aβ42, Aβ40, Aβ42/Aβ40, Aβ42/Aβ38, AGE, PTGENDER, APOE4 | 0.901 | 0.029 |
| Aβ42, Aβ40, Aβ42/Aβ40, Aβ40/Aβ38, PTGENDER, APOE4 | 0.896 | 0.025 |
| Aβ42, Aβ40, Aβ38, AGE | 0.886 | 0.015 |
| Aβ42, Aβ38, Aβ42/Aβ40, PTEDUCAT | 0.903 | 0.032 |
| Aβ42, Aβ40, Aβ38, Aβ42/Aβ38, Aβ40/Aβ38, AGE | 0.908 | 0.036 |
| Aβ42, Aβ40, Aβ38, Aβ42/Aβ40, Aβ42/Aβ38, Aβ40/Aβ38, AGE | 0.905 | 0.034 |
| Aβ42, Aβ40, Aβ42/Aβ38, PTEDUCAT, APOE4 | 0.901 | 0.030 |
| Aβ42, Aβ40, Aβ38, Aβ42/Aβ40, Aβ42/Aβ38, Aβ40/Aβ38, AGE, APOE4 | 0.899 | 0.028 |
| Aβ42, Aβ42/Aβ40, Aβ42/Aβ38, Aβ40/Aβ38, AGE, PTGENDER, APOE4 | 0.899 | 0.028 |
| Aβ42, Aβ38, Aβ42/Aβ40, Aβ42/Aβ38, PTGENDER | 0.897 | 0.026 |
| Aβ42, Aβ38, Aβ42/Aβ40, Aβ42/Aβ38, Aβ40/Aβ38, AGE, PTGENDER, APOE4 | 0.907 | 0.035 |
| Aβ40, Aβ38, Aβ42/Aβ40, Aβ40/Aβ38, PTEDUCAT, PTGENDER | 0.888 | 0.016 |
| Aβ40, Aβ42/Aβ40, Aβ42/Aβ38, Aβ40/Aβ38, AGE, PTGENDER | 0.888 | 0.016 |
| Aβ40, Aβ38, Aβ42/Aβ40, AGE, PTEDUCAT | 0.902 | 0.031 |
| Aβ42, Aβ40, Aβ38, Aβ42/Aβ40, PTEDUCAT, APOE4 | 0.898 | 0.027 |
| Aβ42, Aβ42/Aβ40, Aβ40/Aβ38, AGE, PTGENDER, APOE4 | 0.902 | 0.031 |
| Aβ42, Aβ40, Aβ38, PTEDUCAT, APOE4 | 0.893 | 0.022 |
| Aβ42, Aβ40, Aβ42/Aβ40, Aβ42/Aβ38, PTEDUCAT, PTGENDER, APOE4 | 0.905 | 0.035 |
| Aβ40, Aβ42/Aβ40, Aβ40/Aβ38, AGE, PTGENDER, APOE4 | 0.896 | 0.025 |
| Aβ42, Aβ40, Aβ40/Aβ38, AGE, PTGENDER | 0.903 | 0.032 |
| Aβ40, Aβ42/Aβ40, Aβ42/Aβ38, Aβ40/Aβ38, PTEDUCAT, PTGENDER, APOE4 | 0.887 | 0.016 |
| Aβ40, Aβ42/Aβ38, PTEDUCAT, PTGENDER, APOE4 | 0.902 | 0.032 |
| Aβ42, Aβ40, Aβ42/Aβ38, PTEDUCAT | 0.893 | 0.023 |
| Aβ40, Aβ38, Aβ42/Aβ38, Aβ40/Aβ38, AGE | 0.909 | 0.039 |
| Aβ42, Aβ40, Aβ38, Aβ42/Aβ40, Aβ42/Aβ38, AGE, APOE4 | 0.903 | 0.033 |
| Aβ42, Aβ40, Aβ38, Aβ40/Aβ38, PTEDUCAT, PTGENDER | 0.889 | 0.019 |
| Aβ42, Aβ40, Aβ38, Aβ42/Aβ38, Aβ40/Aβ38, AGE, PTEDUCAT, PTGENDER, APOE4 | 0.904 | 0.034 |
| Aβ40, Aβ38, Aβ42/Aβ40, Aβ42/Aβ38, PTEDUCAT, PTGENDER, APOE4 | 0.904 | 0.033 |
| Aβ40, Aβ38, Aβ42/Aβ38, Aβ40/Aβ38, PTGENDER | 0.900 | 0.029 |
| Aβ42, Aβ40, Aβ38 | 0.892 | 0.021 |
| Aβ42, Aβ40, APOE4 | 0.895 | 0.025 |
| Aβ42, Aβ38, Aβ42/Aβ40, Aβ42/Aβ38, APOE4 | 0.895 | 0.025 |
| Aβ42, Aβ40, Aβ38, Aβ42/Aβ40, Aβ42/Aβ38, PTGENDER, APOE4 | 0.901 | 0.031 |
| Aβ42, Aβ38, Aβ42/Aβ40, APOE4 | 0.902 | 0.032 |
| Aβ40, Aβ38, Aβ42/Aβ38, Aβ40/Aβ38, AGE, PTEDUCAT, PTGENDER, APOE4 | 0.906 | 0.037 |
| Aβ42, Aβ42/Aβ40, Aβ42/Aβ38, PTEDUCAT, PTGENDER, APOE4 | 0.899 | 0.030 |
| Aβ42, Aβ38, Aβ42/Aβ38, Aβ40/Aβ38, PTEDUCAT | 0.904 | 0.034 |
| Aβ42, Aβ38, Aβ42/Aβ40, AGE, PTGENDER | 0.901 | 0.031 |
| Aβ42, Aβ40, Aβ38, Aβ42/Aβ40, Aβ42/Aβ38, AGE, PTEDUCAT, PTGENDER, APOE4 | 0.901 | 0.031 |
| Aβ42, Aβ40, Aβ38, Aβ42/Aβ40, AGE, PTEDUCAT, PTGENDER | 0.896 | 0.027 |
| Aβ42, Aβ40, Aβ42/Aβ40, Aβ40/Aβ38, AGE, APOE4 | 0.896 | 0.027 |
| Aβ42, Aβ40, Aβ38, Aβ42/Aβ40, Aβ42/Aβ38, APOE4 | 0.896 | 0.027 |
| Aβ42, Aβ42/Aβ40, Aβ40/Aβ38, AGE, PTEDUCAT, PTGENDER, APOE4 | 0.902 | 0.033 |
| Aβ42, Aβ42/Aβ38, Aβ40/Aβ38, AGE | 0.896 | 0.027 |
| Aβ42, Aβ38, Aβ42/Aβ40, AGE, PTEDUCAT, PTGENDER | 0.903 | 0.034 |
| Aβ42, Aβ38, Aβ42/Aβ40, Aβ40/Aβ38, PTEDUCAT, PTGENDER, APOE4 | 0.901 | 0.031 |
| Aβ42, Aβ40, Aβ38, Aβ42/Aβ40, Aβ42/Aβ38, PTEDUCAT | 0.895 | 0.026 |
| Aβ42, Aβ38, Aβ42/Aβ40, PTGENDER | 0.905 | 0.035 |
| Aβ42, Aβ40, Aβ42/Aβ38, PTEDUCAT, PTGENDER, APOE4 | 0.896 | 0.027 |
| Aβ42, Aβ40, Aβ38, Aβ42/Aβ38, APOE4 | 0.899 | 0.030 |
| Aβ38, Aβ42/Aβ38, Aβ40/Aβ38, PTGENDER | 0.907 | 0.038 |
| Aβ42, Aβ40, Aβ38, Aβ42/Aβ40, AGE, APOE4 | 0.896 | 0.027 |
| Aβ42, Aβ38, Aβ42/Aβ40, AGE, PTEDUCAT, APOE4 | 0.904 | 0.035 |
| Aβ42, Aβ40, Aβ42/Aβ38, Aβ40/Aβ38, AGE, APOE4 | 0.898 | 0.029 |
| Aβ42, Aβ40, Aβ38, Aβ40/Aβ38, AGE | 0.891 | 0.022 |
| Aβ40, Aβ42/Aβ38, PTGENDER, APOE4 | 0.901 | 0.033 |
| Aβ42, Aβ38, Aβ42/Aβ40 | 0.901 | 0.032 |
| Aβ42, Aβ38, Aβ42/Aβ40, Aβ40/Aβ38, AGE, PTGENDER, APOE4 | 0.902 | 0.033 |
| Aβ42, Aβ40, Aβ38, Aβ42/Aβ40, Aβ42/Aβ38, PTEDUCAT, APOE4 | 0.902 | 0.034 |
| Aβ42, Aβ40, Aβ38, Aβ40/Aβ38, PTEDUCAT, APOE4 | 0.883 | 0.014 |
| Aβ38, Aβ42/Aβ40, Aβ42/Aβ38, Aβ40/Aβ38, PTGENDER | 0.884 | 0.015 |
| Aβ42, Aβ40, Aβ42/Aβ38, Aβ40/Aβ38, AGE, PTEDUCAT, APOE4 | 0.893 | 0.024 |
| Aβ40, Aβ38, Aβ42/Aβ40, Aβ42/Aβ38, APOE4 | 0.901 | 0.032 |
| Aβ40, Aβ42/Aβ38, Aβ40/Aβ38, AGE, PTEDUCAT, PTGENDER | 0.895 | 0.027 |
| Aβ38, Aβ42/Aβ40, Aβ42/Aβ38, Aβ40/Aβ38, PTEDUCAT, APOE4 | 0.887 | 0.019 |
| Aβ38, Aβ42/Aβ40, Aβ40/Aβ38, PTGENDER | 0.899 | 0.031 |
| Aβ42, Aβ38, Aβ40/Aβ38, AGE, PTEDUCAT | 0.896 | 0.028 |
| Aβ42, Aβ40, Aβ42/Aβ40, Aβ42/Aβ38, Aβ40/Aβ38, PTGENDER, APOE4 | 0.898 | 0.029 |
| Aβ38, Aβ42/Aβ40, Aβ42/Aβ38, Aβ40/Aβ38, AGE, PTEDUCAT, PTGENDER | 0.887 | 0.018 |
| Aβ40, Aβ38, Aβ42/Aβ38 | 0.891 | 0.023 |
| Aβ40, Aβ38, Aβ42/Aβ40, Aβ42/Aβ38, AGE, PTEDUCAT, PTGENDER | 0.902 | 0.033 |
| Aβ42, Aβ40, AGE, APOE4 | 0.893 | 0.025 |
| Aβ42, Aβ40, Aβ42/Aβ38, Aβ40/Aβ38, PTEDUCAT, PTGENDER, APOE4 | 0.894 | 0.026 |
| Aβ40, Aβ38, Aβ42/Aβ40, Aβ42/Aβ38, Aβ40/Aβ38, AGE, PTEDUCAT, APOE4 | 0.895 | 0.026 |
| Aβ42, Aβ40, Aβ38, Aβ40/Aβ38, AGE, PTEDUCAT | 0.901 | 0.033 |
| Aβ40, Aβ38, Aβ42/Aβ40, Aβ42/Aβ38, AGE, PTGENDER, APOE4 | 0.905 | 0.037 |
| Aβ42, Aβ40, Aβ42/Aβ38, AGE | 0.897 | 0.029 |
| Aβ42, Aβ38, Aβ42/Aβ40, AGE, PTGENDER, APOE4 | 0.902 | 0.033 |
| Aβ40, Aβ38, Aβ42/Aβ40, Aβ42/Aβ38, AGE, PTEDUCAT, APOE4 | 0.901 | 0.033 |
| Aβ38, Aβ42/Aβ40, Aβ42/Aβ38, Aβ40/Aβ38, PTEDUCAT, PTGENDER | 0.884 | 0.016 |
| Aβ42, Aβ38, Aβ42/Aβ40, PTEDUCAT, PTGENDER | 0.901 | 0.034 |
| Aβ42, Aβ40, Aβ42/Aβ38, Aβ40/Aβ38, AGE, PTEDUCAT, PTGENDER | 0.893 | 0.025 |
| Aβ42, Aβ40, Aβ38, Aβ42/Aβ38, Aβ40/Aβ38, AGE, PTEDUCAT, PTGENDER | 0.904 | 0.036 |
| Aβ42, Aβ38, Aβ42/Aβ40, PTGENDER, APOE4 | 0.902 | 0.034 |
| Aβ40, Aβ38, Aβ42/Aβ38, AGE, PTEDUCAT | 0.889 | 0.021 |
| Aβ38, Aβ42/Aβ38, Aβ40/Aβ38 | 0.904 | 0.036 |
| Aβ42, Aβ40, Aβ38, Aβ42/Aβ38, AGE | 0.895 | 0.027 |
| Aβ42, Aβ40, AGE, PTEDUCAT, PTGENDER | 0.894 | 0.026 |
| Aβ42, Aβ40, Aβ38, Aβ42/Aβ38, Aβ40/Aβ38, AGE, APOE4 | 0.900 | 0.033 |
| Aβ42, Aβ38, Aβ42/Aβ40, PTEDUCAT, APOE4 | 0.901 | 0.033 |
| Aβ42, Aβ38, Aβ42/Aβ40, Aβ42/Aβ38, Aβ40/Aβ38, PTEDUCAT, PTGENDER, APOE4 | 0.888 | 0.021 |
| Aβ42, Aβ38, Aβ42/Aβ40, Aβ42/Aβ38, PTEDUCAT, APOE4 | 0.891 | 0.023 |
| Aβ42, Aβ40, Aβ42/Aβ40, Aβ42/Aβ38, Aβ40/Aβ38, AGE, PTEDUCAT, PTGENDER, APOE4 | 0.887 | 0.020 |
| Aβ42, Aβ42/Aβ38, Aβ40/Aβ38 | 0.895 | 0.028 |
| Aβ40, Aβ42/Aβ40, Aβ42/Aβ38, Aβ40/Aβ38, APOE4 | 0.889 | 0.022 |
| Aβ42, Aβ40, Aβ38, Aβ42/Aβ38, AGE, PTEDUCAT, APOE4 | 0.898 | 0.030 |
| Aβ42, Aβ40, Aβ38, Aβ42/Aβ38, Aβ40/Aβ38, AGE, PTEDUCAT, APOE4 | 0.901 | 0.033 |
| Aβ42, Aβ40, Aβ38, Aβ42/Aβ40, PTEDUCAT, PTGENDER | 0.894 | 0.027 |
| Aβ42, Aβ40, Aβ42/Aβ38, Aβ40/Aβ38, AGE, PTGENDER, APOE4 | 0.896 | 0.028 |
| Aβ42, Aβ40, Aβ38, Aβ42/Aβ38, Aβ40/Aβ38, PTEDUCAT, PTGENDER | 0.896 | 0.028 |
| Aβ42, Aβ40, PTGENDER, APOE4 | 0.891 | 0.024 |
| Aβ40, Aβ42/Aβ38, Aβ40/Aβ38, AGE, PTGENDER | 0.893 | 0.026 |
| Aβ42, Aβ40, AGE, PTGENDER | 0.892 | 0.025 |
| Aβ40, Aβ42/Aβ40, Aβ40/Aβ38, PTEDUCAT, APOE4 | 0.898 | 0.031 |
| Aβ40, Aβ38, Aβ42/Aβ40, Aβ42/Aβ38, PTEDUCAT | 0.905 | 0.038 |
| Aβ42, Aβ38, Aβ42/Aβ40, Aβ42/Aβ38, PTEDUCAT, PTGENDER | 0.893 | 0.026 |
| Aβ42, Aβ40, Aβ42/Aβ40, Aβ42/Aβ38, Aβ40/Aβ38, AGE, PTGENDER, APOE4 | 0.884 | 0.018 |
| Aβ38, Aβ42/Aβ40, Aβ40/Aβ38, PTEDUCAT, PTGENDER | 0.900 | 0.033 |
| Aβ38, Aβ42/Aβ40, Aβ42/Aβ38, PTEDUCAT, PTGENDER, APOE4 | 0.885 | 0.018 |
| Aβ42, Aβ38, Aβ40/Aβ38, APOE4 | 0.890 | 0.023 |
| Aβ42, Aβ40, Aβ42/Aβ38, PTEDUCAT, PTGENDER | 0.901 | 0.034 |
| Aβ40, Aβ38, Aβ42/Aβ40, PTEDUCAT, APOE4 | 0.892 | 0.025 |
| Aβ42, Aβ40, Aβ38, APOE4 | 0.892 | 0.025 |
| Aβ40, Aβ42/Aβ38, PTEDUCAT, PTGENDER | 0.900 | 0.034 |
| Aβ40, Aβ38, Aβ42/Aβ38, AGE, PTGENDER, APOE4 | 0.903 | 0.037 |
| Aβ38, Aβ42/Aβ40, Aβ42/Aβ38, AGE | 0.888 | 0.022 |
| Aβ42, Aβ38, Aβ42/Aβ40, Aβ42/Aβ38, AGE, APOE4 | 0.895 | 0.028 |
| Aβ40, Aβ38, Aβ42/Aβ40, PTEDUCAT, PTGENDER | 0.897 | 0.031 |
| Aβ42, Aβ42/Aβ38, Aβ40/Aβ38, PTEDUCAT | 0.897 | 0.031 |
| Aβ42, Aβ40, Aβ38, Aβ42/Aβ38, PTGENDER | 0.887 | 0.021 |
| Aβ38, Aβ42/Aβ38, Aβ40/Aβ38, AGE, APOE4 | 0.908 | 0.042 |
| Aβ42, Aβ40, Aβ42/Aβ40, Aβ40/Aβ38, AGE, PTEDUCAT, PTGENDER, APOE4 | 0.900 | 0.034 |
| Aβ42, Aβ40, Aβ38, Aβ42/Aβ40, PTGENDER, APOE4 | 0.898 | 0.032 |
| Aβ42, Aβ40, Aβ38, Aβ42/Aβ38, Aβ40/Aβ38, AGE, PTGENDER, APOE4 | 0.903 | 0.037 |
| Aβ42, Aβ40, Aβ42/Aβ38, PTGENDER, APOE4 | 0.898 | 0.032 |
| Aβ42, Aβ42/Aβ38, Aβ40/Aβ38, AGE, PTEDUCAT | 0.895 | 0.029 |
| Aβ38, Aβ42/Aβ40, Aβ42/Aβ38, Aβ40/Aβ38, APOE4 | 0.885 | 0.019 |
| Aβ42, Aβ38, Aβ40/Aβ38, PTGENDER | 0.888 | 0.022 |
| Aβ42, Aβ40, Aβ38, Aβ42/Aβ40, Aβ42/Aβ38, PTEDUCAT, PTGENDER, APOE4 | 0.904 | 0.038 |
| Aβ42, Aβ38, Aβ40/Aβ38, AGE, PTGENDER | 0.894 | 0.028 |
| Aβ40, Aβ42/Aβ40, Aβ42/Aβ38, AGE, PTEDUCAT, PTGENDER, APOE4 | 0.897 | 0.031 |
| Aβ42, Aβ40, Aβ42/Aβ40, Aβ42/Aβ38, AGE, PTEDUCAT, PTGENDER, APOE4 | 0.901 | 0.036 |
| Aβ40, Aβ38, Aβ42/Aβ40, Aβ42/Aβ38, Aβ40/Aβ38, PTEDUCAT, APOE4 | 0.895 | 0.029 |
| Aβ40, Aβ38, Aβ42/Aβ38, Aβ40/Aβ38, AGE, PTGENDER | 0.901 | 0.036 |
| Aβ42, Aβ38, Aβ42/Aβ40, Aβ42/Aβ38, AGE, PTGENDER, APOE4 | 0.898 | 0.032 |
| Aβ40, Aβ38, Aβ42/Aβ40, Aβ40/Aβ38, PTEDUCAT | 0.898 | 0.032 |
| Aβ42, Aβ40, Aβ42/Aβ38, PTGENDER | 0.900 | 0.035 |
| Aβ42, Aβ40, Aβ38, Aβ42/Aβ38, PTGENDER, APOE4 | 0.896 | 0.030 |
| Aβ40, Aβ38, Aβ42/Aβ40, AGE, PTEDUCAT, PTGENDER, APOE4 | 0.903 | 0.038 |
| Aβ42, Aβ38, Aβ42/Aβ40, Aβ40/Aβ38, AGE, PTEDUCAT, PTGENDER, APOE4 | 0.899 | 0.034 |
| Aβ42, Aβ40, Aβ38, Aβ40/Aβ38, APOE4 | 0.886 | 0.021 |
| Aβ38, Aβ42/Aβ40, Aβ42/Aβ38, PTGENDER | 0.885 | 0.019 |
| Aβ40, Aβ38, Aβ42/Aβ40, Aβ42/Aβ38, AGE, PTGENDER | 0.890 | 0.025 |
| Aβ40, Aβ38, Aβ42/Aβ40, AGE, PTEDUCAT, APOE4 | 0.902 | 0.036 |
| Aβ42, Aβ38, Aβ42/Aβ40, Aβ42/Aβ38, Aβ40/Aβ38, AGE, PTEDUCAT, PTGENDER | 0.885 | 0.020 |
| Aβ38, Aβ42/Aβ38, Aβ40/Aβ38, PTEDUCAT | 0.905 | 0.040 |
| Aβ40, Aβ38, Aβ42/Aβ40, AGE, PTEDUCAT, PTGENDER | 0.898 | 0.033 |
| Aβ42, Aβ40, Aβ38, Aβ42/Aβ38, AGE, APOE4 | 0.898 | 0.033 |
| Aβ42, Aβ38, Aβ42/Aβ38, Aβ40/Aβ38, AGE, PTEDUCAT, PTGENDER | 0.895 | 0.030 |
| Aβ42, Aβ40, Aβ38, Aβ42/Aβ38, AGE, PTEDUCAT | 0.896 | 0.031 |
| Aβ40, Aβ38, Aβ42/Aβ40, Aβ42/Aβ38, PTGENDER, APOE4 | 0.901 | 0.036 |
| Aβ42, Aβ40, Aβ38, Aβ42/Aβ40, PTEDUCAT, PTGENDER, APOE4 | 0.900 | 0.035 |
| Aβ42, Aβ38, Aβ42/Aβ40, PTEDUCAT, PTGENDER, APOE4 | 0.900 | 0.035 |
| Aβ40, Aβ38, Aβ42/Aβ40, AGE, PTGENDER, APOE4 | 0.901 | 0.036 |
| Aβ42, Aβ42/Aβ38, Aβ40/Aβ38, PTGENDER | 0.899 | 0.034 |
| Aβ38, Aβ42/Aβ40, Aβ40/Aβ38, APOE4 | 0.897 | 0.033 |
| Aβ42, Aβ38, Aβ42/Aβ40, Aβ42/Aβ38, AGE, PTEDUCAT | 0.894 | 0.029 |
| Aβ42, Aβ40, Aβ42/Aβ38, Aβ40/Aβ38, PTEDUCAT, APOE4 | 0.892 | 0.028 |
| Aβ42, Aβ40, Aβ38, Aβ42/Aβ40, Aβ42/Aβ38, PTEDUCAT, PTGENDER | 0.903 | 0.039 |
| Aβ42, Aβ42/Aβ38, Aβ40/Aβ38, AGE, PTGENDER | 0.897 | 0.032 |
| Aβ38, Aβ42/Aβ40, Aβ42/Aβ38 | 0.884 | 0.019 |
| Aβ38, Aβ42/Aβ40, Aβ42/Aβ38, Aβ40/Aβ38, AGE, PTGENDER | 0.886 | 0.021 |
| Aβ42, Aβ40, Aβ38, Aβ42/Aβ38, Aβ40/Aβ38, AGE, PTGENDER | 0.903 | 0.038 |
| Aβ42, Aβ40, Aβ38, Aβ42/Aβ40, Aβ42/Aβ38, AGE, PTEDUCAT, APOE4 | 0.897 | 0.033 |
| Aβ38, Aβ42/Aβ40, Aβ42/Aβ38, AGE, PTGENDER | 0.884 | 0.020 |
| Aβ40, Aβ42/Aβ38, Aβ40/Aβ38, AGE, PTEDUCAT, APOE4 | 0.894 | 0.029 |
| Aβ40, Aβ42/Aβ38, AGE, PTEDUCAT, PTGENDER | 0.898 | 0.034 |
| Aβ38, Aβ42/Aβ40, Aβ42/Aβ38, Aβ40/Aβ38, AGE, APOE4 | 0.888 | 0.024 |
| Aβ42, Aβ40, Aβ38, PTGENDER | 0.892 | 0.028 |
| Aβ40, Aβ38, Aβ42/Aβ38, Aβ40/Aβ38, AGE, PTEDUCAT, PTGENDER | 0.900 | 0.035 |
| Aβ42, Aβ38, Aβ42/Aβ38, Aβ40/Aβ38, PTGENDER, APOE4 | 0.896 | 0.031 |
| Aβ38, Aβ42/Aβ38, Aβ40/Aβ38, AGE, PTEDUCAT, APOE4 | 0.900 | 0.036 |
| Aβ42, Aβ40, Aβ38, Aβ42/Aβ40, Aβ42/Aβ38, PTGENDER | 0.890 | 0.026 |
| Aβ42, Aβ38, Aβ42/Aβ40, Aβ42/Aβ38, PTGENDER, APOE4 | 0.894 | 0.030 |
| Aβ40, Aβ38, Aβ42/Aβ40, AGE, APOE4 | 0.895 | 0.031 |
| Aβ42, Aβ38, Aβ42/Aβ38, Aβ40/Aβ38, PTEDUCAT, PTGENDER, APOE4 | 0.884 | 0.020 |
| Aβ40, Aβ42/Aβ38, AGE, PTEDUCAT, APOE4 | 0.898 | 0.035 |
| Aβ42, Aβ40, Aβ38, AGE, PTEDUCAT | 0.893 | 0.029 |
| Aβ42, Aβ38, Aβ40/Aβ38, AGE, PTEDUCAT, PTGENDER | 0.889 | 0.026 |
| Aβ42, Aβ40, Aβ38, Aβ42/Aβ40, AGE, PTEDUCAT, PTGENDER, APOE4 | 0.900 | 0.036 |
| Aβ42, Aβ40, Aβ38, Aβ42/Aβ40, Aβ42/Aβ38, Aβ40/Aβ38, AGE, PTEDUCAT, APOE4 | 0.890 | 0.027 |
| Aβ38, Aβ42/Aβ40, Aβ40/Aβ38, PTEDUCAT, APOE4 | 0.896 | 0.033 |
| Aβ42, Aβ40, Aβ38, Aβ42/Aβ38 | 0.891 | 0.028 |
| Aβ38, Aβ42/Aβ40, Aβ42/Aβ38, PTEDUCAT, APOE4 | 0.883 | 0.020 |
| Aβ42, Aβ42/Aβ38, Aβ40/Aβ38, AGE, APOE4 | 0.871 | 0.008 |
| Aβ40, Aβ42/Aβ38, Aβ40/Aβ38, AGE, PTGENDER, APOE4 | 0.887 | 0.024 |
| Aβ40, Aβ38, Aβ42/Aβ40, PTEDUCAT, PTGENDER, APOE4 | 0.897 | 0.035 |
| Aβ42, Aβ38, Aβ42/Aβ40, Aβ42/Aβ38, AGE, PTEDUCAT, PTGENDER | 0.894 | 0.031 |
| Aβ40, Aβ38, Aβ42/Aβ40, Aβ42/Aβ38, PTGENDER | 0.882 | 0.019 |
| Aβ38, Aβ42/Aβ40, Aβ42/Aβ38, AGE, PTEDUCAT | 0.892 | 0.030 |
| Aβ42, Aβ38, Aβ42/Aβ40, Aβ42/Aβ38, AGE, PTEDUCAT, APOE4 | 0.896 | 0.033 |
| Aβ38, Aβ42/Aβ40, Aβ42/Aβ38, Aβ40/Aβ38, PTGENDER, APOE4 | 0.882 | 0.019 |
| Aβ42, Aβ40, Aβ42/Aβ38, AGE, PTGENDER, APOE4 | 0.892 | 0.030 |
| Aβ42, Aβ40, Aβ40/Aβ38, AGE, PTEDUCAT, PTGENDER, APOE4 | 0.884 | 0.022 |
| Aβ40, Aβ38, Aβ42/Aβ38, AGE, PTEDUCAT, PTGENDER | 0.896 | 0.034 |
| Aβ42, Aβ40, Aβ38, AGE, PTGENDER | 0.892 | 0.030 |
| Aβ42, Aβ38, Aβ42/Aβ40, Aβ42/Aβ38 | 0.889 | 0.027 |
| Aβ42, Aβ38, Aβ42/Aβ38, Aβ40/Aβ38, AGE, PTEDUCAT, APOE4 | 0.897 | 0.036 |
| Aβ40, Aβ42/Aβ40, Aβ40/Aβ38, AGE, PTEDUCAT, PTGENDER, APOE4 | 0.892 | 0.030 |
| Aβ42, Aβ40, Aβ38, Aβ42/Aβ40, Aβ42/Aβ38, AGE, PTEDUCAT, PTGENDER | 0.897 | 0.035 |
| Aβ42, Aβ40, Aβ38, Aβ42/Aβ40, Aβ42/Aβ38, AGE, PTGENDER, APOE4 | 0.896 | 0.034 |
| Aβ42, Aβ40, Aβ38, Aβ42/Aβ40, Aβ42/Aβ38, Aβ40/Aβ38, AGE, PTGENDER, APOE4 | 0.897 | 0.035 |
| Aβ38, Aβ42/Aβ38, Aβ40/Aβ38, AGE | 0.894 | 0.033 |
| Aβ38, Aβ42/Aβ40, Aβ42/Aβ38, Aβ40/Aβ38, PTEDUCAT, PTGENDER, APOE4 | 0.882 | 0.020 |
| Aβ42, Aβ38, Aβ42/Aβ38, Aβ40/Aβ38, PTEDUCAT, APOE4 | 0.896 | 0.035 |
| Aβ38, Aβ42/Aβ40, Aβ42/Aβ38, Aβ40/Aβ38, AGE, PTGENDER, APOE4 | 0.884 | 0.022 |
| Aβ42, Aβ40, Aβ38, PTGENDER, APOE4 | 0.894 | 0.032 |
| Aβ38, Aβ42/Aβ38, Aβ40/Aβ38, APOE4 | 0.894 | 0.033 |
| Aβ42, Aβ40, Aβ38, Aβ42/Aβ38, Aβ40/Aβ38, AGE, PTEDUCAT | 0.889 | 0.028 |
| Aβ42, Aβ38, Aβ42/Aβ40, Aβ42/Aβ38, Aβ40/Aβ38, AGE, PTEDUCAT, PTGENDER, APOE4 | 0.892 | 0.031 |
| Aβ42, Aβ40, PTEDUCAT, PTGENDER, APOE4 | 0.882 | 0.020 |
| Aβ40, Aβ38, Aβ42/Aβ38, APOE4 | 0.899 | 0.037 |
| Aβ42, Aβ40, Aβ38, AGE, PTEDUCAT, APOE4 | 0.894 | 0.032 |
| Aβ42, Aβ40, Aβ42/Aβ38, AGE, PTEDUCAT, PTGENDER | 0.895 | 0.034 |
| Aβ42, Aβ40, Aβ38, Aβ40/Aβ38, AGE, PTGENDER, APOE4 | 0.892 | 0.031 |
| Aβ38, Aβ42/Aβ38, Aβ40/Aβ38, AGE, PTEDUCAT, PTGENDER | 0.897 | 0.036 |
| Aβ40, Aβ38, Aβ42/Aβ38, AGE | 0.891 | 0.030 |
| Aβ42, Aβ38, Aβ42/Aβ40, AGE, PTEDUCAT, PTGENDER, APOE4 | 0.898 | 0.037 |
| Aβ40, Aβ38, Aβ42/Aβ38, PTGENDER, APOE4 | 0.895 | 0.034 |
| Aβ42, Aβ40, Aβ38, PTEDUCAT, PTGENDER | 0.898 | 0.037 |
| Aβ42, Aβ40, Aβ38, Aβ40/Aβ38, AGE, PTGENDER | 0.889 | 0.028 |
| Aβ42, Aβ38, Aβ42/Aβ40, Aβ42/Aβ38, PTEDUCAT, PTGENDER, APOE4 | 0.890 | 0.029 |
| Aβ40, Aβ38, Aβ42/Aβ40, Aβ42/Aβ38, Aβ40/Aβ38, AGE, PTEDUCAT, PTGENDER, APOE4 | 0.903 | 0.042 |
| Aβ38, Aβ42/Aβ40, Aβ40/Aβ38, AGE, PTGENDER | 0.893 | 0.032 |
| Aβ42, Aβ40, Aβ38, Aβ42/Aβ38, AGE, PTGENDER | 0.893 | 0.033 |
| Aβ42, Aβ40, Aβ38, PTEDUCAT | 0.890 | 0.029 |
| Aβ40, Aβ38, Aβ42/Aβ38, PTGENDER | 0.903 | 0.042 |
| Aβ42, Aβ38, Aβ42/Aβ38, Aβ40/Aβ38, AGE, PTEDUCAT, PTGENDER, APOE4 | 0.900 | 0.039 |
| Aβ38, Aβ42/Aβ40, Aβ42/Aβ38, AGE, PTGENDER, APOE4 | 0.887 | 0.027 |
| Aβ38, Aβ42/Aβ40, Aβ42/Aβ38, Aβ40/Aβ38, AGE, PTEDUCAT, APOE4 | 0.886 | 0.026 |
| Aβ42, Aβ40, Aβ40/Aβ38, AGE, APOE4 | 0.889 | 0.029 |
| Aβ40, Aβ38, Aβ42/Aβ38, Aβ40/Aβ38, AGE, PTEDUCAT, APOE4 | 0.890 | 0.030 |
| Aβ38, Aβ42/Aβ40, Aβ42/Aβ38, PTGENDER, APOE4 | 0.883 | 0.023 |
| Aβ42, Aβ40, Aβ40/Aβ38, PTEDUCAT, PTGENDER, APOE4 | 0.888 | 0.028 |
| Aβ40, Aβ38, Aβ42/Aβ38, PTEDUCAT, PTGENDER, APOE4 | 0.896 | 0.037 |
| Aβ42, Aβ40, Aβ38, Aβ42/Aβ38, PTEDUCAT, APOE4 | 0.892 | 0.033 |
| Aβ38, Aβ42/Aβ38, Aβ40/Aβ38, PTGENDER, APOE4 | 0.903 | 0.043 |
| Aβ42, Aβ40, Aβ38, AGE, APOE4 | 0.893 | 0.034 |
| Aβ42, Aβ42/Aβ38, Aβ40/Aβ38, APOE4 | 0.890 | 0.031 |
| Aβ42, Aβ40, Aβ42/Aβ38, AGE, PTEDUCAT, PTGENDER, APOE4 | 0.896 | 0.037 |
| Aβ42, Aβ40, Aβ40/Aβ38, AGE, PTEDUCAT, APOE4 | 0.883 | 0.024 |
| Aβ38, Aβ42/Aβ40, APOE4 | 0.896 | 0.036 |
| Aβ38, Aβ42/Aβ40, Aβ40/Aβ38, PTEDUCAT | 0.893 | 0.034 |
| Aβ42, Aβ40, Aβ38, Aβ40/Aβ38, AGE, APOE4 | 0.892 | 0.033 |
| Aβ42, Aβ38, Aβ42/Aβ38, Aβ40/Aβ38, APOE4 | 0.890 | 0.031 |
| Aβ38, Aβ42/Aβ40, PTEDUCAT | 0.893 | 0.034 |
| Aβ42, Aβ38, Aβ40/Aβ38, PTEDUCAT, PTGENDER | 0.888 | 0.029 |
| Aβ38, Aβ42/Aβ40, Aβ42/Aβ38, APOE4 | 0.881 | 0.022 |
| Aβ42, Aβ42/Aβ38, Aβ40/Aβ38, PTGENDER, APOE4 | 0.892 | 0.034 |
| Aβ40, Aβ38, Aβ42/Aβ38, Aβ40/Aβ38, PTEDUCAT, PTGENDER | 0.891 | 0.033 |
| Aβ42, Aβ38, Aβ40/Aβ38, AGE, PTGENDER, APOE4 | 0.889 | 0.031 |
| Aβ38, Aβ42/Aβ40, PTGENDER, APOE4 | 0.886 | 0.028 |
| Aβ42, Aβ38, Aβ42/Aβ38, Aβ40/Aβ38, PTGENDER | 0.895 | 0.038 |
| Aβ42, Aβ40, AGE, PTEDUCAT, PTGENDER, APOE4 | 0.890 | 0.032 |
| Aβ38, Aβ42/Aβ38, Aβ40/Aβ38, AGE, PTEDUCAT | 0.905 | 0.047 |
| Aβ42, Aβ40, Aβ42/Aβ38, AGE, PTGENDER | 0.891 | 0.034 |
| Aβ42, Aβ38, Aβ40/Aβ38, PTEDUCAT, PTGENDER, APOE4 | 0.886 | 0.029 |
| Aβ42, Aβ38, Aβ42/Aβ40, Aβ42/Aβ38, AGE, PTEDUCAT, PTGENDER, APOE4 | 0.892 | 0.035 |
| Aβ38, Aβ42/Aβ40, Aβ42/Aβ38, PTEDUCAT | 0.886 | 0.029 |
| Aβ42, Aβ40, Aβ38, Aβ42/Aβ38, PTEDUCAT | 0.889 | 0.032 |
| Aβ38, Aβ42/Aβ40, Aβ40/Aβ38, PTEDUCAT, PTGENDER, APOE4 | 0.891 | 0.034 |
| Aβ42, Aβ38, Aβ42/Aβ38, Aβ40/Aβ38, AGE, PTGENDER | 0.889 | 0.032 |
| Aβ42, Aβ40, Aβ38, AGE, PTEDUCAT, PTGENDER, APOE4 | 0.892 | 0.035 |
| Aβ42, Aβ42/Aβ38, Aβ40/Aβ38, PTEDUCAT, PTGENDER | 0.895 | 0.038 |
| Aβ42, Aβ42/Aβ38, Aβ40/Aβ38, PTEDUCAT, APOE4 | 0.891 | 0.034 |
| Aβ40, Aβ38, Aβ42/Aβ38, AGE, PTGENDER | 0.890 | 0.033 |
| Aβ38, Aβ42/Aβ40, Aβ42/Aβ38, AGE, PTEDUCAT, APOE4 | 0.887 | 0.030 |
| Aβ42, Aβ40, Aβ38, Aβ42/Aβ38, AGE, PTEDUCAT, PTGENDER | 0.893 | 0.037 |
| Aβ40, Aβ38, Aβ42/Aβ38, PTEDUCAT | 0.889 | 0.033 |
| Aβ38, Aβ42/Aβ40, Aβ42/Aβ38, AGE, APOE4 | 0.884 | 0.028 |
| Aβ40, Aβ38, Aβ42/Aβ38, AGE, PTEDUCAT, APOE4 | 0.890 | 0.033 |
| Aβ38, Aβ42/Aβ38, Aβ40/Aβ38, AGE, PTGENDER | 0.903 | 0.046 |
| Aβ38, Aβ42/Aβ40, Aβ40/Aβ38, AGE, APOE4 | 0.888 | 0.033 |
| Aβ38, Aβ42/Aβ40, AGE, PTEDUCAT | 0.892 | 0.037 |
| Aβ40, Aβ38, Aβ42/Aβ38, AGE, PTEDUCAT, PTGENDER, APOE4 | 0.895 | 0.039 |
| Aβ38, Aβ42/Aβ40, Aβ40/Aβ38, AGE, PTEDUCAT, PTGENDER | 0.882 | 0.027 |
| Aβ42, Aβ40, Aβ38, Aβ42/Aβ38, PTEDUCAT, PTGENDER | 0.889 | 0.033 |
| Aβ38, Aβ42/Aβ40, Aβ40/Aβ38, AGE, PTEDUCAT | 0.897 | 0.041 |
| Aβ40, Aβ38, Aβ42/Aβ38, AGE, APOE4 | 0.888 | 0.032 |
| Aβ42, Aβ40, Aβ38, AGE, PTEDUCAT, PTGENDER | 0.889 | 0.034 |
| Aβ42, Aβ40, Aβ38, PTEDUCAT, PTGENDER, APOE4 | 0.888 | 0.033 |
| Aβ42, Aβ42/Aβ38, Aβ40/Aβ38, AGE, PTEDUCAT, APOE4 | 0.889 | 0.034 |
| Aβ42, Aβ38, Aβ40/Aβ38, AGE, PTEDUCAT, APOE4 | 0.889 | 0.034 |
| Aβ42, Aβ38, Aβ42/Aβ38, Aβ40/Aβ38, AGE, APOE4 | 0.891 | 0.036 |
| Aβ38, Aβ42/Aβ40, Aβ42/Aβ38, AGE, PTEDUCAT, PTGENDER, APOE4 | 0.884 | 0.030 |
| Aβ42, Aβ42/Aβ38, Aβ40/Aβ38, AGE, PTEDUCAT, PTGENDER | 0.889 | 0.035 |
| Aβ38, Aβ42/Aβ40 | 0.891 | 0.037 |
| Aβ42, Aβ40, Aβ38, AGE, PTGENDER, APOE4 | 0.889 | 0.035 |
| Aβ42, Aβ40, Aβ38, Aβ42/Aβ38, AGE, PTEDUCAT, PTGENDER, APOE4 | 0.896 | 0.043 |
| Aβ38, Aβ42/Aβ40, Aβ42/Aβ38, Aβ40/Aβ38, AGE, PTEDUCAT, PTGENDER, APOE4 | 0.880 | 0.027 |
| Aβ40, Aβ38, Aβ42/Aβ38, PTEDUCAT, PTGENDER | 0.887 | 0.034 |
| Aβ42, Aβ42/Aβ38, Aβ40/Aβ38, AGE, PTGENDER, APOE4 | 0.890 | 0.037 |
| Aβ38, Aβ42/Aβ40, AGE, PTEDUCAT, APOE4 | 0.891 | 0.038 |
| Aβ38, Aβ42/Aβ38, Aβ40/Aβ38, PTEDUCAT, APOE4 | 0.891 | 0.038 |
| Aβ42, Aβ40, Aβ38, Aβ42/Aβ38, AGE, PTGENDER, APOE4 | 0.889 | 0.037 |
| Aβ42, Aβ42/Aβ38, Aβ40/Aβ38, PTEDUCAT, PTGENDER, APOE4 | 0.891 | 0.038 |
| Aβ42, Aβ38, Aβ42/Aβ38, Aβ40/Aβ38, PTEDUCAT, PTGENDER | 0.887 | 0.035 |
| Aβ38, Aβ42/Aβ40, PTEDUCAT, PTGENDER | 0.889 | 0.037 |
| Aβ38, Aβ42/Aβ40, Aβ40/Aβ38, AGE, PTEDUCAT, APOE4 | 0.888 | 0.036 |
| Aβ40, Aβ38, Aβ42/Aβ38, PTEDUCAT, APOE4 | 0.884 | 0.032 |
| Aβ38, Aβ42/Aβ40, PTEDUCAT, APOE4 | 0.888 | 0.037 |
| Aβ42, Aβ38, Aβ40/Aβ38, AGE, APOE4 | 0.885 | 0.034 |
| Aβ42, Aβ40, Aβ38, Aβ42/Aβ38, PTEDUCAT, PTGENDER, APOE4 | 0.887 | 0.036 |
| Aβ38, Aβ42/Aβ40, PTGENDER | 0.887 | 0.036 |
| Aβ42, Aβ38, Aβ40/Aβ38, PTEDUCAT, APOE4 | 0.885 | 0.035 |
| Aβ38, Aβ42/Aβ38, Aβ40/Aβ38, PTEDUCAT, PTGENDER | 0.885 | 0.035 |
| Aβ42, Aβ38, Aβ42/Aβ38, Aβ40/Aβ38, AGE, PTGENDER, APOE4 | 0.894 | 0.043 |
| Aβ42, Aβ40, Aβ38, Aβ40/Aβ38, AGE, PTEDUCAT, APOE4 | 0.887 | 0.036 |
| Aβ38, Aβ42/Aβ40, Aβ40/Aβ38, AGE, PTGENDER, APOE4 | 0.887 | 0.036 |
| Aβ38, Aβ42/Aβ38, Aβ40/Aβ38, AGE, PTGENDER, APOE4 | 0.897 | 0.047 |
| Aβ42, Aβ42/Aβ38, Aβ40/Aβ38, AGE, PTEDUCAT, PTGENDER, APOE4 | 0.886 | 0.036 |
| Aβ38, Aβ42/Aβ40, AGE | 0.887 | 0.038 |
| Aβ38, Aβ42/Aβ38, Aβ40/Aβ38, PTEDUCAT, PTGENDER, APOE4 | 0.886 | 0.036 |
| Aβ38, Aβ42/Aβ38, Aβ40/Aβ38, AGE, PTEDUCAT, PTGENDER, APOE4 | 0.896 | 0.046 |
| Aβ38, Aβ42/Aβ40, AGE, PTGENDER, APOE4 | 0.880 | 0.032 |
| Aβ38, Aβ42/Aβ40, Aβ40/Aβ38, AGE, PTEDUCAT, PTGENDER, APOE4 | 0.885 | 0.038 |
| Aβ38, Aβ42/Aβ40, Aβ42/Aβ38, AGE, PTEDUCAT, PTGENDER | 0.882 | 0.036 |
| Aβ42, Aβ38, Aβ40/Aβ38, AGE, PTEDUCAT, PTGENDER, APOE4 | 0.885 | 0.039 |
| Aβ40, AGE, PTGENDER, APOE4 | 0.879 | 0.033 |
| Aβ40, AGE, APOE4 | 0.879 | 0.033 |
| Aβ40, Aβ38, Aβ40/Aβ38, PTGENDER | 0.880 | 0.034 |
| Aβ38, Aβ40/Aβ38, PTGENDER, APOE4 | 0.884 | 0.039 |
| Aβ40, AGE, PTEDUCAT, APOE4 | 0.881 | 0.037 |
| Aβ40, Aβ40/Aβ38, PTGENDER, APOE4 | 0.866 | 0.022 |
| Aβ38, Aβ42/Aβ40, AGE, PTGENDER | 0.885 | 0.041 |
| Aβ38, Aβ42/Aβ40, PTEDUCAT, PTGENDER, APOE4 | 0.885 | 0.042 |
| Aβ40, APOE4 | 0.874 | 0.031 |
| Aβ38, Aβ42/Aβ40, AGE, PTEDUCAT, PTGENDER | 0.886 | 0.044 |
| Aβ40, Aβ40/Aβ38, AGE, PTEDUCAT, APOE4 | 0.875 | 0.033 |
| Aβ40, Aβ40/Aβ38, AGE, PTGENDER, APOE4 | 0.874 | 0.033 |
| Aβ40, AGE, PTEDUCAT, PTGENDER, APOE4 | 0.881 | 0.040 |
| Aβ40, AGE | 0.859 | 0.019 |
| Aβ42, Aβ42/Aβ38, APOE4 | 0.885 | 0.045 |
| Aβ38, Aβ42/Aβ40, AGE, PTEDUCAT, PTGENDER, APOE4 | 0.877 | 0.038 |
| Aβ40, Aβ40/Aβ38, AGE | 0.855 | 0.016 |
| Aβ40, Aβ40/Aβ38, AGE, PTEDUCAT | 0.868 | 0.029 |
| Aβ38, Aβ42/Aβ40, AGE, APOE4 | 0.884 | 0.044 |
| Aβ40, Aβ40/Aβ38, PTEDUCAT | 0.868 | 0.029 |
| Aβ40, AGE, PTEDUCAT | 0.870 | 0.031 |
| Aβ40, Aβ38, Aβ40/Aβ38 | 0.872 | 0.033 |
| Aβ42, Aβ38, Aβ42/Aβ38, PTEDUCAT | 0.886 | 0.048 |
| Aβ40, PTGENDER, APOE4 | 0.875 | 0.038 |
| Aβ42, Aβ42/Aβ38 | 0.886 | 0.049 |
| Aβ40, AGE, PTGENDER | 0.865 | 0.029 |
| Aβ40, Aβ40/Aβ38 | 0.864 | 0.028 |
| Aβ42, Aβ38, Aβ42/Aβ38, AGE | 0.883 | 0.047 |
| Aβ42, Aβ42/Aβ38, AGE, PTGENDER | 0.886 | 0.050 |
| Aβ42, Aβ42/Aβ38, PTEDUCAT | 0.886 | 0.051 |
| Aβ40, Aβ40/Aβ38, AGE, APOE4 | 0.871 | 0.035 |
| Aβ40, Aβ38, Aβ40/Aβ38, AGE, PTGENDER | 0.854 | 0.019 |
| Aβ42, Aβ42/Aβ38, AGE, PTEDUCAT, PTGENDER, APOE4 | 0.872 | 0.037 |
| Aβ42, Aβ38, Aβ42/Aβ38 | 0.882 | 0.047 |
| Aβ40, Aβ38, Aβ40/Aβ38, AGE, PTEDUCAT, PTGENDER, APOE4 | 0.864 | 0.030 |
| Aβ42, Aβ38, Aβ42/Aβ38, PTGENDER | 0.887 | 0.053 |
| Aβ42, Aβ38 | 0.881 | 0.047 |
| Aβ40, Aβ38, Aβ40/Aβ38, AGE, PTGENDER, APOE4 | 0.869 | 0.035 |
| Aβ38, Aβ42/Aβ38, PTGENDER | 0.887 | 0.053 |
| Aβ42, Aβ38, Aβ42/Aβ38, APOE4 | 0.876 | 0.043 |
| Aβ42, Aβ42/Aβ38, AGE | 0.885 | 0.052 |
| Aβ40, Aβ40/Aβ38, AGE, PTEDUCAT, PTGENDER | 0.864 | 0.031 |
| Aβ40, Aβ40/Aβ38, PTGENDER | 0.858 | 0.025 |
| Aβ40, Aβ38, Aβ40/Aβ38, APOE4 | 0.872 | 0.038 |
| Aβ38, Aβ42/Aβ38, PTEDUCAT, APOE4 | 0.878 | 0.044 |
| Aβ42, Aβ38, AGE, PTGENDER | 0.876 | 0.043 |
| Aβ40, Aβ38, Aβ40/Aβ38, AGE | 0.870 | 0.037 |
| Aβ38, Aβ42/Aβ38 | 0.884 | 0.051 |
| Aβ42, Aβ38, Aβ42/Aβ38, AGE, APOE4 | 0.877 | 0.044 |
| Aβ38, Aβ40/Aβ38 | 0.862 | 0.030 |
| Aβ40, PTEDUCAT, APOE4 | 0.874 | 0.042 |
| Aβ40, Aβ40/Aβ38, PTEDUCAT, PTGENDER | 0.866 | 0.034 |
| Aβ42, Aβ42/Aβ38, AGE, PTEDUCAT | 0.885 | 0.053 |
| Aβ42, Aβ42/Aβ38, PTEDUCAT, APOE4 | 0.884 | 0.052 |
| Aβ40, AGE, PTEDUCAT, PTGENDER | 0.867 | 0.036 |
| Aβ40, Aβ40/Aβ38, APOE4 | 0.862 | 0.031 |
| Aβ40, PTGENDER | 0.868 | 0.037 |
| Aβ40, Aβ38, AGE | 0.853 | 0.022 |
| Aβ40, Aβ38, PTGENDER, APOE4 | 0.871 | 0.041 |
| Aβ42, Aβ42/Aβ38, PTGENDER | 0.886 | 0.055 |
| Aβ40, Aβ40/Aβ38, PTEDUCAT, APOE4 | 0.865 | 0.035 |
| Aβ40, Aβ38, APOE4 | 0.874 | 0.043 |
| Aβ40, Aβ38, AGE, PTEDUCAT, APOE4 | 0.867 | 0.037 |
| Aβ40, Aβ40/Aβ38, AGE, PTEDUCAT, PTGENDER, APOE4 | 0.875 | 0.045 |
| Aβ38, Aβ40/Aβ38, AGE, PTGENDER, APOE4 | 0.858 | 0.028 |
| Aβ40, Aβ40/Aβ38, PTEDUCAT, PTGENDER, APOE4 | 0.867 | 0.037 |
| Aβ40, PTEDUCAT, PTGENDER, APOE4 | 0.873 | 0.043 |
| Aβ40, Aβ38, AGE, PTGENDER, APOE4 | 0.872 | 0.042 |
| Aβ38, Aβ40/Aβ38, PTEDUCAT, APOE4 | 0.867 | 0.038 |
| Aβ40, Aβ40/Aβ38, AGE, PTGENDER | 0.864 | 0.034 |
| Aβ42, Aβ38, Aβ42/Aβ38, AGE, PTGENDER | 0.881 | 0.052 |
| Aβ38, Aβ40/Aβ38, AGE | 0.857 | 0.028 |
| Aβ40, Aβ38, Aβ40/Aβ38, AGE, PTEDUCAT, APOE4 | 0.868 | 0.039 |
| Aβ42, Aβ42/Aβ38, PTGENDER, APOE4 | 0.886 | 0.057 |
| Aβ38, Aβ40/Aβ38, AGE, APOE4 | 0.859 | 0.030 |
| Aβ38, Aβ42/Aβ38, AGE, PTEDUCAT | 0.879 | 0.050 |
| Aβ42, Aβ38, Aβ42/Aβ38, PTGENDER, APOE4 | 0.874 | 0.045 |
| Aβ42, Aβ38, Aβ42/Aβ38, PTEDUCAT, APOE4 | 0.877 | 0.049 |
| Aβ38, Aβ40/Aβ38, AGE, PTEDUCAT, APOE4 | 0.859 | 0.031 |
| Aβ42, Aβ38, Aβ42/Aβ38, AGE, PTGENDER, APOE4 | 0.874 | 0.046 |
| Aβ38, Aβ42/Aβ38, PTEDUCAT | 0.877 | 0.049 |
| Aβ38, Aβ42/Aβ38, AGE, PTEDUCAT, APOE4 | 0.880 | 0.053 |
| Aβ42, Aβ38, Aβ42/Aβ38, AGE, PTEDUCAT | 0.881 | 0.053 |
| Aβ40, Aβ38, Aβ40/Aβ38, PTEDUCAT, PTGENDER, APOE4 | 0.867 | 0.039 |
| Aβ38, Aβ42/Aβ38, AGE | 0.874 | 0.047 |
| Aβ40 | 0.863 | 0.036 |
| Aβ42, Aβ42/Aβ38, AGE, PTEDUCAT, APOE4 | 0.882 | 0.055 |
| Aβ40, Aβ38, AGE, APOE4 | 0.866 | 0.039 |
| Aβ40, Aβ38, Aβ40/Aβ38, PTEDUCAT | 0.864 | 0.038 |
| Aβ42, Aβ42/Aβ38, AGE, APOE4 | 0.880 | 0.054 |
| Aβ40, Aβ38, Aβ40/Aβ38, PTEDUCAT, APOE4 | 0.869 | 0.042 |
| Aβ38, Aβ40/Aβ38, PTEDUCAT, PTGENDER, APOE4 | 0.869 | 0.043 |
| Aβ42, Aβ42/Aβ38, PTEDUCAT, PTGENDER, APOE4 | 0.883 | 0.057 |
| Aβ42, Aβ38, Aβ42/Aβ38, AGE, PTEDUCAT, APOE4 | 0.875 | 0.049 |
| Aβ38, Aβ40/Aβ38, AGE, PTGENDER | 0.850 | 0.025 |
| Aβ40, Aβ38, AGE, PTEDUCAT, PTGENDER | 0.865 | 0.039 |
| Aβ42, Aβ42/Aβ38, AGE, PTGENDER, APOE4 | 0.881 | 0.056 |
| Aβ42, Aβ42/Aβ38, PTEDUCAT, PTGENDER | 0.883 | 0.058 |
| Aβ38, Aβ42/Aβ38, PTEDUCAT, PTGENDER | 0.875 | 0.050 |
| Aβ42, Aβ38, PTGENDER | 0.878 | 0.053 |
| Aβ38, Aβ40/Aβ38, PTGENDER | 0.868 | 0.043 |
| Aβ42, Aβ38, Aβ42/Aβ38, PTEDUCAT, PTGENDER, APOE4 | 0.873 | 0.048 |
| Aβ42, Aβ38, Aβ42/Aβ38, PTEDUCAT, PTGENDER | 0.879 | 0.054 |
| Aβ38, Aβ42/Aβ38, APOE4 | 0.878 | 0.054 |
| Aβ38, Aβ42/Aβ38, AGE, APOE4 | 0.878 | 0.054 |
| Aβ42, Aβ38, Aβ42/Aβ38, AGE, PTEDUCAT, PTGENDER | 0.879 | 0.056 |
| Aβ40, Aβ38, AGE, PTEDUCAT | 0.864 | 0.041 |
| Aβ40, PTEDUCAT, PTGENDER | 0.866 | 0.043 |
| Aβ40, Aβ38, Aβ40/Aβ38, AGE, PTEDUCAT, PTGENDER | 0.858 | 0.035 |
| Aβ40, PTEDUCAT | 0.865 | 0.042 |
| Aβ42, Aβ42/Aβ38, AGE, PTEDUCAT, PTGENDER | 0.881 | 0.059 |
| Aβ42, Aβ38, Aβ42/Aβ38, AGE, PTEDUCAT, PTGENDER, APOE4 | 0.873 | 0.051 |
| Aβ40, Aβ38, Aβ40/Aβ38, AGE, APOE4 | 0.861 | 0.039 |
| Aβ40, Aβ38, Aβ40/Aβ38, AGE, PTEDUCAT | 0.862 | 0.040 |
| Aβ40, Aβ38, AGE, PTGENDER | 0.862 | 0.040 |
| Aβ40, Aβ38, Aβ40/Aβ38, PTEDUCAT, PTGENDER | 0.863 | 0.042 |
| Aβ42, Aβ38, PTEDUCAT, APOE4 | 0.872 | 0.050 |
| Aβ42, Aβ38, PTGENDER, APOE4 | 0.869 | 0.048 |
| Aβ38, Aβ40/Aβ38, AGE, PTEDUCAT | 0.849 | 0.028 |
| Aβ40, Aβ38, AGE, PTEDUCAT, PTGENDER, APOE4 | 0.866 | 0.045 |
| Aβ38, Aβ42/Aβ38, AGE, PTEDUCAT, PTGENDER | 0.874 | 0.054 |
| Aβ40, Aβ38, PTEDUCAT, PTGENDER, APOE4 | 0.870 | 0.050 |
| Aβ42, Aβ38, AGE | 0.869 | 0.049 |
| Aβ42, Aβ38, AGE, PTEDUCAT | 0.872 | 0.052 |
| Aβ42, Aβ38, APOE4 | 0.853 | 0.034 |
| Aβ40, Aβ38, PTEDUCAT, APOE4 | 0.866 | 0.047 |
| Aβ42, Aβ38, AGE, PTGENDER, APOE4 | 0.863 | 0.045 |
| Aβ38, Aβ42/Aβ38, AGE, PTEDUCAT, PTGENDER, APOE4 | 0.865 | 0.047 |
| Aβ38, Aβ40/Aβ38, AGE, PTEDUCAT, PTGENDER, APOE4 | 0.863 | 0.046 |
| Aβ38, Aβ42/Aβ38, AGE, PTGENDER | 0.868 | 0.050 |
| Aβ40, Aβ38, PTEDUCAT | 0.862 | 0.045 |
| Aβ38, AGE, APOE4 | 0.853 | 0.036 |
| Aβ38, AGE, PTEDUCAT, APOE4 | 0.867 | 0.050 |
| Aβ40, Aβ38 | 0.860 | 0.043 |
| Aβ42, Aβ38, AGE, APOE4 | 0.864 | 0.048 |
| Aβ38, Aβ42/Aβ38, PTGENDER, APOE4 | 0.870 | 0.054 |
| Aβ38, Aβ40/Aβ38, AGE, PTEDUCAT, PTGENDER | 0.846 | 0.030 |
| Aβ40, Aβ38, Aβ40/Aβ38, PTGENDER, APOE4 | 0.864 | 0.048 |
| Aβ38, Aβ42/Aβ38, AGE, PTGENDER, APOE4 | 0.865 | 0.050 |
| Aβ40, Aβ38, PTGENDER | 0.859 | 0.043 |
| Aβ42, Aβ38, PTEDUCAT | 0.867 | 0.052 |
| Aβ38, Aβ40/Aβ38, APOE4 | 0.860 | 0.047 |
| Aβ38, Aβ42/Aβ38, PTEDUCAT, PTGENDER, APOE4 | 0.873 | 0.059 |
| Aβ40, Aβ38, PTEDUCAT, PTGENDER | 0.859 | 0.046 |
| Aβ42, Aβ38, AGE, PTEDUCAT, APOE4 | 0.871 | 0.058 |
| Aβ38, Aβ40/Aβ38, PTEDUCAT, PTGENDER | 0.857 | 0.044 |
| Aβ38, APOE4 | 0.853 | 0.044 |
| Aβ38, Aβ40/Aβ38, PTEDUCAT | 0.858 | 0.049 |
| Aβ42, Aβ38, AGE, PTEDUCAT, PTGENDER | 0.866 | 0.057 |
| Aβ42, Aβ38, PTEDUCAT, PTGENDER, APOE4 | 0.867 | 0.060 |
| Aβ38, PTGENDER, APOE4 | 0.862 | 0.057 |
| Aβ42, Aβ38, PTEDUCAT, PTGENDER | 0.863 | 0.058 |
| Aβ38, PTEDUCAT, APOE4 | 0.861 | 0.058 |
| Aβ38, AGE, PTGENDER, APOE4 | 0.848 | 0.045 |
| Aβ38, AGE, PTEDUCAT, PTGENDER, APOE4 | 0.863 | 0.061 |
| Aβ42, Aβ38, AGE, PTEDUCAT, PTGENDER, APOE4 | 0.866 | 0.065 |
| Aβ38, PTEDUCAT, PTGENDER, APOE4 | 0.859 | 0.059 |
| Aβ38, AGE, PTGENDER | 0.847 | 0.049 |
| Aβ38, PTEDUCAT | 0.844 | 0.047 |
| Aβ38, PTGENDER | 0.849 | 0.052 |
| Aβ38, AGE, PTEDUCAT | 0.847 | 0.052 |
| Aβ38, AGE | 0.845 | 0.049 |
| Aβ38 | 0.846 | 0.051 |
| Aβ38, AGE, PTEDUCAT, PTGENDER | 0.846 | 0.053 |
| Aβ38, PTEDUCAT, PTGENDER | 0.847 | 0.057 |
| Aβ42/Aβ38, Aβ40/Aβ38, PTGENDER, APOE4 | 0.707 | 0.034 |
| Aβ42/Aβ40, Aβ42/Aβ38, Aβ40/Aβ38, PTEDUCAT | 0.697 | 0.046 |
| Aβ42/Aβ40, Aβ40/Aβ38, PTGENDER, APOE4 | 0.687 | 0.051 |
| Aβ42/Aβ38, Aβ40/Aβ38, PTEDUCAT, PTGENDER, APOE4 | 0.670 | 0.035 |
| Aβ42/Aβ40, Aβ42/Aβ38, Aβ40/Aβ38 | 0.670 | 0.036 |
| Aβ42/Aβ40, Aβ42/Aβ38, Aβ40/Aβ38, PTGENDER | 0.667 | 0.037 |
| Aβ42/Aβ38, Aβ40/Aβ38 | 0.664 | 0.035 |
| Aβ42/Aβ40, Aβ42/Aβ38, Aβ40/Aβ38, PTGENDER, APOE4 | 0.664 | 0.038 |
| Aβ42/Aβ40, PTEDUCAT | 0.644 | 0.020 |
| Aβ42/Aβ38, Aβ40/Aβ38, AGE, APOE4 | 0.658 | 0.035 |
| Aβ42/Aβ40, AGE, PTGENDER | 0.659 | 0.039 |
| Aβ42/Aβ38, Aβ40/Aβ38, PTGENDER | 0.658 | 0.038 |
| Aβ42/Aβ38, Aβ40/Aβ38, APOE4 | 0.663 | 0.044 |
| Aβ42/Aβ40, Aβ42/Aβ38, Aβ40/Aβ38, APOE4 | 0.667 | 0.049 |
| Aβ42, APOE4 | 0.636 | 0.018 |
| Aβ42, PTEDUCAT, APOE4 | 0.639 | 0.022 |
| Aβ42, AGE, PTEDUCAT, PTGENDER | 0.645 | 0.028 |
| Aβ42, AGE, PTEDUCAT | 0.648 | 0.033 |
| Aβ42, Aβ40/Aβ38, PTEDUCAT, APOE4 | 0.648 | 0.032 |
| Aβ42, Aβ40/Aβ38, PTEDUCAT, PTGENDER, APOE4 | 0.642 | 0.028 |
| Aβ42/Aβ40, Aβ42/Aβ38, Aβ40/Aβ38, AGE, PTEDUCAT | 0.656 | 0.042 |
| Aβ42/Aβ40, Aβ40/Aβ38, PTEDUCAT | 0.668 | 0.055 |
| Aβ42/Aβ40, Aβ40/Aβ38, AGE, PTGENDER, APOE4 | 0.660 | 0.047 |
| Aβ42/Aβ40, PTEDUCAT, PTGENDER | 0.656 | 0.043 |
| Aβ42/Aβ38, Aβ40/Aβ38, AGE, PTEDUCAT | 0.663 | 0.051 |
| Aβ42, Aβ40/Aβ38, AGE, PTEDUCAT | 0.654 | 0.043 |
| Aβ42, Aβ40/Aβ38, AGE, PTEDUCAT, APOE4 | 0.663 | 0.051 |
| Aβ42/Aβ40, Aβ40/Aβ38, APOE4 | 0.678 | 0.067 |
| Aβ42, Aβ40/Aβ38, AGE, PTEDUCAT, PTGENDER, APOE4 | 0.660 | 0.050 |
| Aβ42, Aβ40/Aβ38, AGE, PTEDUCAT, PTGENDER | 0.648 | 0.038 |
| Aβ42, PTEDUCAT, PTGENDER, APOE4 | 0.632 | 0.022 |
| Aβ42/Aβ40, Aβ40/Aβ38, PTEDUCAT, PTGENDER, APOE4 | 0.658 | 0.048 |
| Aβ42/Aβ38, Aβ40/Aβ38, PTEDUCAT | 0.670 | 0.060 |
| Aβ42/Aβ40, Aβ42/Aβ38, Aβ40/Aβ38, PTEDUCAT, PTGENDER | 0.659 | 0.050 |
| Aβ42/Aβ40, Aβ40/Aβ38, PTEDUCAT, PTGENDER | 0.656 | 0.048 |
| Aβ42/Aβ40, Aβ40/Aβ38, AGE, PTGENDER | 0.625 | 0.018 |
| Aβ42/Aβ40, Aβ42/Aβ38, PTEDUCAT | 0.655 | 0.048 |
| Aβ42/Aβ38, Aβ40/Aβ38, AGE | 0.684 | 0.078 |
| Aβ42/Aβ38, Aβ40/Aβ38, AGE, PTGENDER | 0.684 | 0.078 |
| Aβ42/Aβ40, Aβ42/Aβ38, PTEDUCAT, PTGENDER | 0.653 | 0.047 |
| Aβ42, PTEDUCAT | 0.633 | 0.027 |
| Aβ42, Aβ40/Aβ38 | 0.687 | 0.082 |
| Aβ42/Aβ38 | 0.641 | 0.036 |
| Aβ42/Aβ40, Aβ42/Aβ38, Aβ40/Aβ38, AGE | 0.670 | 0.065 |
| Aβ42, Aβ40/Aβ38, AGE, PTGENDER | 0.642 | 0.037 |
| Aβ42, Aβ40/Aβ38, PTEDUCAT | 0.650 | 0.046 |
| Aβ42, PTGENDER, APOE4 | 0.633 | 0.030 |
| Aβ42/Aβ40, Aβ42/Aβ38, Aβ40/Aβ38, AGE, APOE4 | 0.635 | 0.033 |
| Aβ42, AGE, PTEDUCAT, PTGENDER, APOE4 | 0.647 | 0.046 |
| Aβ42/Aβ40, Aβ42/Aβ38, Aβ40/Aβ38, AGE, PTGENDER | 0.653 | 0.052 |
| Aβ42/Aβ38, Aβ40/Aβ38, PTEDUCAT, PTGENDER | 0.634 | 0.033 |
| Aβ42, Aβ40/Aβ38, PTGENDER | 0.681 | 0.081 |
| Aβ42/Aβ40, Aβ40/Aβ38, AGE, PTEDUCAT, PTGENDER | 0.621 | 0.021 |
| Aβ42, AGE, PTGENDER | 0.630 | 0.031 |
| Aβ42/Aβ40, Aβ42/Aβ38, Aβ40/Aβ38, PTEDUCAT, APOE4 | 0.655 | 0.056 |
| Aβ42/Aβ40, Aβ42/Aβ38, Aβ40/Aβ38, AGE, PTGENDER, APOE4 | 0.625 | 0.027 |
| Aβ42/Aβ40, Aβ42/Aβ38, AGE, PTEDUCAT, PTGENDER | 0.660 | 0.061 |
| Aβ42, AGE, PTEDUCAT, APOE4 | 0.657 | 0.059 |
| Aβ42/Aβ40, Aβ40/Aβ38, AGE | 0.660 | 0.062 |
| Aβ42/Aβ40, Aβ42/Aβ38, AGE, PTEDUCAT | 0.659 | 0.061 |
| Aβ42/Aβ40, PTGENDER | 0.629 | 0.031 |
| Aβ42/Aβ40, Aβ40/Aβ38 | 0.643 | 0.046 |
| Aβ42, AGE, PTGENDER, APOE4 | 0.648 | 0.051 |
| Aβ42, Aβ40/Aβ38, PTEDUCAT, PTGENDER | 0.644 | 0.046 |
| Aβ42/Aβ38, Aβ40/Aβ38, AGE, PTGENDER, APOE4 | 0.651 | 0.054 |
| Aβ42/Aβ38, Aβ40/Aβ38, AGE, PTEDUCAT, PTGENDER | 0.650 | 0.054 |
| Aβ42, Aβ40/Aβ38, APOE4 | 0.678 | 0.082 |
| Aβ42/Aβ40, Aβ42/Aβ38, Aβ40/Aβ38, AGE, PTEDUCAT, PTGENDER | 0.671 | 0.076 |
| Aβ40/Aβ38, PTGENDER | 0.654 | 0.059 |
| Aβ42/Aβ38, Aβ40/Aβ38, AGE, PTEDUCAT, PTGENDER, APOE4 | 0.651 | 0.056 |
| Aβ42/Aβ40 | 0.634 | 0.039 |
| Aβ42/Aβ40, Aβ40/Aβ38, AGE, PTEDUCAT, APOE4 | 0.649 | 0.054 |
| Aβ42/Aβ40, Aβ40/Aβ38, AGE, PTEDUCAT, PTGENDER, APOE4 | 0.664 | 0.069 |
| Aβ42/Aβ38, Aβ40/Aβ38, PTEDUCAT, APOE4 | 0.637 | 0.043 |
| Aβ42, AGE | 0.636 | 0.042 |
| Aβ42/Aβ38, AGE, PTGENDER, APOE4 | 0.652 | 0.059 |
| Aβ42, Aβ40/Aβ38, PTGENDER, APOE4 | 0.685 | 0.093 |
| Aβ42, Aβ40/Aβ38, AGE, APOE4 | 0.637 | 0.045 |
| Aβ42, Aβ40/Aβ38, AGE | 0.637 | 0.045 |
| Aβ42/Aβ38, PTEDUCAT, PTGENDER | 0.652 | 0.060 |
| Aβ42, AGE, APOE4 | 0.668 | 0.076 |
| Aβ40/Aβ38, PTEDUCAT, APOE4 | 0.618 | 0.027 |
| Aβ40/Aβ38, APOE4 | 0.640 | 0.050 |
| Aβ42/Aβ40, Aβ42/Aβ38, PTEDUCAT, PTGENDER, APOE4 | 0.651 | 0.061 |
| Aβ42/Aβ40, AGE, PTEDUCAT | 0.663 | 0.074 |
| Aβ42/Aβ40, Aβ40/Aβ38, AGE, PTEDUCAT | 0.669 | 0.080 |
| Aβ42/Aβ40, Aβ42/Aβ38, PTEDUCAT, APOE4 | 0.649 | 0.060 |
| Aβ42/Aβ40, AGE, PTEDUCAT, PTGENDER | 0.659 | 0.071 |
| Aβ42/Aβ38, PTEDUCAT, PTGENDER, APOE4 | 0.650 | 0.062 |
| Aβ42, PTEDUCAT, PTGENDER | 0.637 | 0.050 |
| Aβ42/Aβ40, Aβ40/Aβ38, PTEDUCAT, APOE4 | 0.646 | 0.060 |
| Aβ42/Aβ40, Aβ40/Aβ38, PTGENDER | 0.654 | 0.069 |
| Aβ42, Aβ40/Aβ38, AGE, PTGENDER, APOE4 | 0.640 | 0.058 |
| Aβ42/Aβ38, PTEDUCAT | 0.651 | 0.069 |
| Aβ42/Aβ40, Aβ42/Aβ38, AGE, PTEDUCAT, PTGENDER, APOE4 | 0.658 | 0.075 |
| Aβ42/Aβ40, Aβ42/Aβ38, Aβ40/Aβ38, PTEDUCAT, PTGENDER, APOE4 | 0.649 | 0.067 |
| AGE, PTGENDER | 0.637 | 0.056 |
| Aβ42/Aβ40, AGE, APOE4 | 0.621 | 0.041 |
| Aβ42/Aβ40, AGE, PTGENDER, APOE4 | 0.655 | 0.074 |
| Aβ42/Aβ40, Aβ42/Aβ38, AGE, PTEDUCAT, APOE4 | 0.657 | 0.077 |
| Aβ42/Aβ38, Aβ40/Aβ38, AGE, PTEDUCAT, APOE4 | 0.646 | 0.066 |
| Aβ42/Aβ40, APOE4 | 0.609 | 0.029 |
| Aβ42/Aβ40, AGE | 0.620 | 0.042 |
| Aβ40/Aβ38, AGE, PTEDUCAT, PTGENDER | 0.659 | 0.081 |
| Aβ42/Aβ40, Aβ40/Aβ38, AGE, APOE4 | 0.646 | 0.069 |
| Aβ42/Aβ40, PTEDUCAT, PTGENDER, APOE4 | 0.646 | 0.069 |
| Aβ40/Aβ38, AGE, APOE4 | 0.659 | 0.083 |
| Aβ42/Aβ40, Aβ42/Aβ38, AGE | 0.631 | 0.055 |
| Aβ42/Aβ40, AGE, PTEDUCAT, PTGENDER, APOE4 | 0.657 | 0.082 |
| Aβ42/Aβ40, AGE, PTEDUCAT, APOE4 | 0.661 | 0.087 |
| Aβ42/Aβ40, Aβ42/Aβ38, PTGENDER | 0.616 | 0.042 |
| Aβ42/Aβ40, Aβ42/Aβ38, AGE, PTGENDER, APOE4 | 0.661 | 0.087 |
| Aβ42/Aβ38, PTEDUCAT, APOE4 | 0.646 | 0.073 |
| Aβ42/Aβ40, Aβ42/Aβ38, AGE, PTGENDER | 0.626 | 0.053 |
| Aβ42/Aβ40, Aβ42/Aβ38, Aβ40/Aβ38, AGE, PTEDUCAT, PTGENDER, APOE4 | 0.647 | 0.074 |
| Aβ42/Aβ40, Aβ42/Aβ38, Aβ40/Aβ38, AGE, PTEDUCAT, APOE4 | 0.651 | 0.079 |
| Aβ42/Aβ38, AGE, PTEDUCAT | 0.654 | 0.081 |
| Aβ42/Aβ40, Aβ42/Aβ38 | 0.618 | 0.047 |
| Aβ42/Aβ40, PTEDUCAT, APOE4 | 0.621 | 0.049 |
| Aβ40/Aβ38, PTEDUCAT, PTGENDER, APOE4 | 0.616 | 0.045 |
| Aβ42/Aβ38, APOE4 | 0.621 | 0.050 |
| Aβ42 | 0.610 | 0.040 |
| Aβ42/Aβ38, AGE | 0.610 | 0.040 |
| Aβ42/Aβ38, AGE, APOE4 | 0.615 | 0.047 |
| Aβ42/Aβ40, PTGENDER, APOE4 | 0.634 | 0.066 |
| Aβ42/Aβ38, AGE, PTEDUCAT, PTGENDER | 0.645 | 0.078 |
| Aβ42, PTGENDER | 0.615 | 0.048 |
| Aβ42/Aβ38, PTGENDER, APOE4 | 0.606 | 0.042 |
| Aβ42/Aβ40, Aβ42/Aβ38, PTGENDER, APOE4 | 0.631 | 0.067 |
| Aβ42/Aβ40, Aβ42/Aβ38, AGE, APOE4 | 0.642 | 0.078 |
| Aβ40/Aβ38, AGE, PTGENDER, APOE4 | 0.622 | 0.064 |
| Aβ40/Aβ38, AGE | 0.641 | 0.083 |
| Aβ40/Aβ38, AGE, PTGENDER | 0.613 | 0.056 |
| Aβ42/Aβ38, AGE, PTGENDER | 0.606 | 0.049 |
| Aβ40/Aβ38, AGE, PTEDUCAT | 0.653 | 0.096 |
| Aβ42/Aβ38, PTGENDER | 0.611 | 0.055 |
| Aβ40/Aβ38, PTEDUCAT, PTGENDER | 0.624 | 0.070 |
| Aβ40/Aβ38, PTGENDER, APOE4 | 0.591 | 0.037 |
| Aβ42/Aβ38, AGE, PTEDUCAT, APOE4 | 0.650 | 0.100 |
| Aβ42/Aβ40, Aβ42/Aβ38, APOE4 | 0.612 | 0.063 |
| Aβ40/Aβ38, PTEDUCAT | 0.619 | 0.070 |
| Aβ40/Aβ38 | 0.626 | 0.079 |
| Aβ40/Aβ38, AGE, PTEDUCAT, APOE4 | 0.656 | 0.109 |
| Aβ42/Aβ38, AGE, PTEDUCAT, PTGENDER, APOE4 | 0.645 | 0.098 |
| AGE, PTGENDER, APOE4 | 0.570 | 0.040 |
| Aβ40/Aβ38, AGE, PTEDUCAT, PTGENDER, APOE4 | 0.643 | 0.122 |
| AGE, APOE4 | 0.565 | 0.052 |
| PTEDUCAT, PTGENDER, APOE4 | 0.543 | 0.036 |
| PTEDUCAT | 0.517 | 0.010 |
| AGE, PTEDUCAT | 0.579 | 0.076 |
| PTEDUCAT, PTGENDER | 0.549 | 0.051 |
| AGE, PTEDUCAT, PTGENDER | 0.561 | 0.066 |
| AGE, PTEDUCAT, APOE4 | 0.593 | 0.100 |
| AGE | 0.560 | 0.068 |
| PTEDUCAT, APOE4 | 0.573 | 0.088 |
| AGE, PTEDUCAT, PTGENDER, APOE4 | 0.569 | 0.093 |
| PTGENDER | 0.521 | 0.055 |
| PTGENDER, APOE4 | 0.516 | 0.064 |
| APOE4 | 0.532 | 0.081 |
