# Supplementary material for: Soluble amyloid-beta isoforms predict downstream Alzheimer’s disease pathology
Source: Cell Biosci. 2021 Dec 11;11:204. doi: 10.1186/s13578-021-00712-3 (PMC8665586; doi:10.1186/s13578-021-00712-3)
Supplement: Supplementary file 3 — Additional file 3. Differentially expressed proteins (DEPs) in the cerebrospinal fluid (CSF) of cognitively unimpaired (CU) tau pathology positive (T+) compared to negative (T−) subjects. Table containing Protein ID, p-value, adjusted p-value, t-value and logFC for differentially expressed proteins in the cerebrospinal fluid of cognitively unimpaired tau pathology positive compared to negative subjects. [file 13578_2021_712_MOESM3_ESM.docx]

Additional file 3

Differentially expressed proteins (DEPs) in the cerebrospinal fluid (CSF) of cognitively unimpaired (CU) tau pathology positive (T+) compared to negative (T−) subjects.

| **Protein ID** | **adj. p-value** | **p-value** | **t-value** | **logFC** |
| --- | --- | --- | --- | --- |
| KPYM.LDIDSPPITAR | 2.35E-07 | 1.71E-09 | 6.8301 | 0.6305 |
| AATC.LALGDDSPALK | 3.52E-07 | 5.14E-09 | 6.5760 | 0.6473 |
| FABPH.SLGVGFATR | 3.94E-07 | 8.64E-09 | 6.4554 | 0.5487 |
| NCAM1.GLGEISAASEFK | 7.64E-07 | 2.62E-08 | 6.1952 | 0.5483 |
| ALDOA.ALQASALK | 7.64E-07 | 3.08E-08 | 6.1567 | 0.5446 |
| NCAM2.ASGSPEPAISWFR | 7.64E-07 | 3.89E-08 | 6.1021 | 0.5255 |
| SPON1.VTLSAAPPSYFR | 7.64E-07 | 3.9E-08 | 6.1010 | 0.5366 |
| TNR21.ASNLIGTYR | 1.39E-06 | 8.89E-08 | 5.9050 | 0.5111 |
| FAM3C.SPFEQHIK | 1.39E-06 | 9.11E-08 | 5.8992 | 0.5679 |
| NEO1.DVVASLVSTR | 1.73E-06 | 1.4E-07 | 5.7962 | 0.4964 |
| CMGA.SGEATDGARPQALPEPMQESK | 1.73E-06 | 1.49E-07 | 5.7807 | 0.7549 |
| OSTP.AIPVAQDLNAPSDWDSR | 1.73E-06 | 1.51E-07 | 5.7776 | 0.4463 |
| CAD13.YEVSSPYFK | 2.29E-06 | 2.17E-07 | 5.6900 | 0.5877 |
| VASN.YLQGSSVQLR | 2.62E-06 | 2.68E-07 | 5.6389 | 0.4485 |
| NRCAM.VFNTPEGVPSAPSSLK | 2.93E-06 | 3.21E-07 | 5.5951 | 0.4953 |
| NICA.APDVTTLPR | 3.39E-06 | 3.96E-07 | 5.5436 | 0.4560 |
| VGF.AYQGVAAPFPK | 3.74E-06 | 4.65E-07 | 5.5045 | 0.7294 |
| CA2D1.FVVTDGGITR | 4.62E-06 | 6.07E-07 | 5.4387 | 0.5654 |
| CYTC.ALDFAVGEYNK | 4.72E-06 | 6.55E-07 | 5.4200 | 0.4144 |
| A4.THPHFVIPYR | 5.38E-06 | 7.86E-07 | 5.3748 | 0.5145 |
| NELL2.FTGSSWIK | 5.98E-06 | 9.16E-07 | 5.3369 | 0.5821 |
| PLDX1.LYGPSEPHSR | 6.01E-06 | 9.65E-07 | 5.3239 | 0.5471 |
| BACE1.SIVDSGTTNLR | 6.5E-06 | 1.09E-06 | 5.2933 | 0.7765 |
| SODC.GDGPVQGIINFEQK | 6.68E-06 | 1.2E-06 | 5.2686 | 0.3944 |
| PTPRN.AEAPALFSR | 6.68E-06 | 1.22E-06 | 5.2657 | 0.5008 |
| SE6L1.ETGTPIWTSR | 7.72E-06 | 1.47E-06 | 5.2195 | 0.4966 |
| I18BP.LWEGSTSR | 9.08E-06 | 1.79E-06 | 5.1693 | 0.4153 |
| UBB.TITLEVEPSDTIENVK | 9.08E-06 | 1.86E-06 | 5.1602 | 0.4312 |
| PTGDS.AQGFTEDTIVFLPQTDK | 9.46E-06 | 2E-06 | 5.1411 | 0.2716 |
| LPHN1.LVVSQLNPYTLR | 1.02E-05 | 2.29E-06 | 5.1076 | 0.4690 |
| KLK6.YTNWIQK | 1.02E-05 | 2.32E-06 | 5.1044 | 0.3426 |
| NCAN.APVLELEK | 1.53E-05 | 3.58E-06 | 4.9938 | 0.5338 |
| AMD.IVQFSPSGK | 1.71E-05 | 4.13E-06 | 4.9573 | 0.5055 |
| MOG.VVHLYR | 1.93E-05 | 4.85E-06 | 4.9156 | 0.3514 |
| DAG1.LVPVVNNR | 1.93E-05 | 4.92E-06 | 4.9119 | 0.3459 |
| PVRL1.ITQVTWQK | 1.99E-05 | 5.23E-06 | 4.8964 | 0.3945 |
| SCG2.IILEALR | 2.46E-05 | 6.64E-06 | 4.8348 | 0.5100 |
| CD59.AGLQVYNK | 2.59E-05 | 7.19E-06 | 4.8141 | 0.3542 |
| L1CAM.WRPVDLAQVK | 2.64E-05 | 7.51E-06 | 4.8027 | 0.4681 |
| B3GN1.TALASGGVLDASGDYR | 2.77E-05 | 8.08E-06 | 4.7838 | 0.2947 |
| NEGR1.SSIIFAGGDK | 3.02E-05 | 9.03E-06 | 4.7547 | 0.4024 |
| AATM.FVTVQTISGTGALR | 3.36E-05 | 1.03E-05 | 4.7204 | 0.4437 |
| CUTA.TQSSLVPALTDFVR | 3.44E-05 | 1.08E-05 | 4.7078 | 0.3996 |
| APOE.LGPLVEQGR | 3.68E-05 | 1.18E-05 | 4.6838 | 0.8539 |
| CNTN1.TTKPYPADIVVQFK | 4.02E-05 | 1.32E-05 | 4.6551 | 0.3649 |
| APLP2.HYQHVLAVDPEK | 4.16E-05 | 1.4E-05 | 4.6399 | 0.3872 |
| LAMB2.AQGIAQGAIR | 5.19E-05 | 1.78E-05 | 4.5756 | 0.4294 |
| NBL1.LALFPDK | 5.67E-05 | 1.99E-05 | 4.5464 | 0.3580 |
| NPTX1.FQLTFPLR | 6.69E-05 | 2.39E-05 | 4.4964 | 0.4558 |
| CSTN1.GNLAGLTLR | 7.28E-05 | 2.69E-05 | 4.4650 | 0.3942 |
| CNTP2.HELQHPIIAR | 7.28E-05 | 2.71E-05 | 4.4630 | 0.4193 |
| B2MG.VNHVTLSQPK | 8.4E-05 | 3.19E-05 | 4.4191 | 0.3026 |
| IGSF8.VVAGEVQVQR | 8.82E-05 | 3.41E-05 | 4.4006 | 0.3314 |
| NPTXR.ELDVLQGR | 0.0001 | 3.95E-05 | 4.3606 | 0.4226 |
| CADM3.EGSVPPLK | 0.0001 | 4.55E-05 | 4.3220 | 0.3690 |
| NRX3A.SDLSFQFK | 0.0001 | 5.03E-05 | 4.2945 | 0.4698 |
| PCSK1.GEAAGAVQELAR | 0.0001 | 5.63E-05 | 4.2637 | 0.5209 |
| NRX2A.LSALTLSTVK | 0.0001 | 5.71E-05 | 4.2598 | 0.4297 |
| ENOG.GNPTVEVDLYTAK | 0.0001 | 6.42E-05 | 4.2271 | 0.3397 |
| CNDP1.WNYIEGTK | 0.0002 | 7.08E-05 | 4.2002 | 0.3970 |
| LRC4B.LTTVPTQAFEYLSK | 0.0002 | 9.98E-05 | 4.1040 | 0.3094 |
| SORC1.TIAVYEEFR | 0.0003 | 0.0001 | 4.0594 | 0.4358 |
| NEUS.ALGITEIFIK | 0.0003 | 0.0002 | 3.9879 | 0.3718 |
| BTD.LSSGLVTAALYGR | 0.0004 | 0.0002 | 3.9503 | 0.2825 |
| SPRL1.VLTHSELAPLR | 0.0004 | 0.0002 | 3.9251 | 0.2374 |
| CNTN2.TTGPGGDGIPAEVHIVR | 0.0004 | 0.0002 | 3.9014 | 0.3339 |
| SCG3.FQDDPDGLHQLDGTPLTAEDIVHK | 0.0004 | 0.0002 | 3.8803 | 0.3521 |
| EXTL2.VIVVWNNIGEK | 0.0006 | 0.0003 | 3.8019 | 0.3763 |
| DIAC.ATYIQNYR | 0.0008 | 0.0004 | 3.6823 | 0.2369 |
| NRX1A.SDLYIGGVAK | 0.0009 | 0.0004 | 3.6717 | 0.3652 |
| SCG1.NYLNYGEEGAPGK | 0.0012 | 0.0006 | 3.5595 | 0.3288 |
| PPN.VHQSPDGTLLIYNLR | 0.0015 | 0.0008 | 3.4955 | 0.3540 |
| CCKN.AHLGALLAR | 0.0019 | 0.0010 | 3.4136 | 0.4164 |
| CH3L1.ILGQQVPYATK | 0.0020 | 0.0011 | 3.4026 | 0.4079 |
| GOLM1.QQLQALSEPQPR | 0.0021 | 0.0012 | 3.3749 | 0.3138 |
| PDYN.FLPSISTK | 0.0022 | 0.0012 | 3.3581 | 0.2754 |
| PEDF.TVQAVLTVPK | 0.0038 | 0.0021 | 3.1791 | 0.1865 |
| FBLN3.SGNENGEFYLR | 0.0056 | 0.0032 | 3.0444 | 0.2896 |
| COCH.GVISNSGGPVR | 0.0057 | 0.0033 | 3.0330 | 0.3318 |
| CD14.SWLAELQQWLKPGLK | 0.0060 | 0.0035 | 3.0136 | 0.2318 |
| MUC18.GATLALTQVTPQDER | 0.0060 | 0.0035 | 3.0105 | 0.2175 |
| PRDX1.DISLSDYK | 0.0062 | 0.0037 | 2.9942 | 0.3289 |
| FBLN1.TGYYFDGISR | 0.0075 | 0.0045 | 2.9234 | 0.1904 |
| CSTN3.ESLLLDTTSLQQR | 0.0097 | 0.0060 | 2.8285 | 0.2271 |
| KLK10.ALQLPYR | 0.0113 | 0.0070 | 2.7705 | 0.4083 |
| CATL1.VFQEPLFYEAPR | 0.0121 | 0.0076 | 2.7412 | 0.1930 |
| GRIA4.LQNILEQIVSVGK | 0.0123 | 0.0078 | 2.7300 | 0.2165 |
| NPTX2.LESLEHQLR | 0.0126 | 0.0081 | 2.7182 | 0.3354 |
| SLIK1.SLPVDVFAGVSLSK | 0.0163 | 0.0106 | 2.6198 | 1.2332 |
| CLUS.VTTVASHTSDSDVPSGVTEVVVK | 0.0198 | 0.0130 | 2.5417 | 0.2042 |
| MIME.ETVIIPNEK | 0.0254 | 0.0168 | 2.4430 | 0.2621 |
| AFAM.FLVNLVK | 0.0370 | 0.0249 | -2.2882 | -0.3535 |
| IBP2.LIQGAPTIR | 0.0374 | 0.0254 | 2.2799 | 0.1711 |
| BASP1.ETPAATEAPSSTPK | 0.0389 | 0.0267 | 2.2590 | 0.9811 |
| GFAP.ALAAELNQLR | 0.0402 | 0.0279 | 2.2409 | 0.2440 |
| CO3.TELRPGETLNVNFLLR | 0.0414 | 0.0290 | 2.2244 | 0.2497 |
| TIMP1.GFQALGDAADIR | 0.0702 | 0.0497 | 1.9935 | 0.1569 |
| ENPP2.SYPEILTLK | 0.0828 | 0.0593 | -1.9145 | -0.0935 |
| TRFM.ADTDGGLIFR | 0.0871 | 0.0630 | 1.8866 | 0.1859 |
| PRDX3.HLSVNDLPVGR | 0.0928 | 0.0677 | 1.8528 | 1.0124 |
| APOD.VLNQELR | 0.1049 | 0.0773 | 1.7901 | 0.1565 |
| PIMT.VQLVVGDGR | 0.1298 | 0.0966 | 1.6820 | 0.1761 |
| PRDX2.GLFIIDGK | 0.1444 | 0.1086 | 1.6234 | 0.5079 |
| ITIH5.SYLEITPSR | 0.1681 | 0.1276 | 1.5400 | 0.1250 |
| CO2.HAIILLTDGK | 0.1828 | 0.1401 | 1.4907 | 0.1601 |
| FMOD.YLPFVPSR | 0.1999 | 0.1547 | 1.4373 | 0.2048 |
| C1QB.LEQGENVFLQATDK | 0.2058 | 0.1607 | 1.4162 | 0.1350 |
| CATD.LVDQNIFSFYLSR | 0.2187 | 0.1724 | -1.3771 | -0.0742 |
| PGRP2.AGLLRPDYALLGHR | 0.2530 | 0.2013 | 1.2888 | 0.2014 |
| TTHY.TSESGELHGLTTEEEFVEGIYK | 0.3755 | 0.3015 | -1.0401 | -0.0738 |
| AACT.ADLSGITGAR | 0.3847 | 0.3117 | 1.0184 | 0.1251 |
| TGFB1.LLAPSDSPEWLSFDVTGVVR | 0.4377 | 0.3578 | 0.9251 | 0.2861 |
