# Supplementary material for: Soluble amyloid-beta isoforms predict downstream Alzheimer’s disease pathology
Source: Cell Biosci. 2021 Dec 11;11:204. doi: 10.1186/s13578-021-00712-3 (PMC8665586; doi:10.1186/s13578-021-00712-3)
Supplement: Supplementary file 4 — Additional file 4. Differentially expressed proteins (DEPs) in the cerebrospinal fluid (CSF) of cognitively unimpaired (CU) neurodegeneration positive (N+) compared to negative (N−) subjects. Table containing Protein ID, p-value, adjusted p-value, t-value and logFC for differentially expressed proteins in the cerebrospinal fluid of cognitively unimpaired neurodegeneration positive compared to negative subjects [file 13578_2021_712_MOESM4_ESM.docx]

Additional file 4

Differentially expressed proteins (DEPs) in the cerebrospinal fluid (CSF) of cognitively unimpaired (CU) neurodegeneration positive (N+) compared to negative (T−) subjects.

| **Protein ID** | **adj. p-value** | **p-value** | **t-value** | **logFC** |
| --- | --- | --- | --- | --- |
| FABPH.SLGVGFATR | 3.50E-08 | 4.20E-10 | 7.1509 | 0.6041 |
| KPYM.LDIDSPPITAR | 3.50E-08 | 5.15E-10 | 7.1041 | 0.6670 |
| ALDOA.ALQASALK | 9.95E-08 | 2.57E-09 | 6.7350 | 0.5962 |
| FAM3C.SPFEQHIK | 9.95E-08 | 2.93E-09 | 6.7054 | 0.6382 |
| AATC.LALGDDSPALK | 1.97E-07 | 8.59E-09 | 6.4556 | 0.6605 |
| NCAM1.GLGEISAASEFK | 1.97E-07 | 8.68E-09 | 6.4531 | 0.5816 |
| NCAM2.IIELSQTTAK | 3.97E-07 | 2.04E-08 | 6.2527 | 0.5980 |
| NEO1.DVVASLVSTR | 4.43E-07 | 2.61E-08 | 6.1956 | 0.5365 |
| SPON1.VTLSAAPPSYFR | 4.83E-07 | 3.19E-08 | 6.1476 | 0.5569 |
| OSTP.AIPVAQDLNAPSDWDSR | 8.00E-07 | 5.88E-08 | 6.0027 | 0.4732 |
| CMGA.SGEATDGARPQALPEPMQESK | 9.12E-07 | 7.38E-08 | 5.9488 | 0.7950 |
| CAD13.YEVSSPYFK | 1.55E-06 | 1.37E-07 | 5.8004 | 0.6151 |
| AMD.IVQFSPSGK | 1.81E-06 | 1.73E-07 | 5.7448 | 0.5814 |
| PLDX1.LYGPSEPHSR | 2.13E-06 | 2.23E-07 | 5.6829 | 0.5920 |
| DAG1.LVPVVNNR | 2.13E-06 | 2.35E-07 | 5.6700 | 0.3970 |
| UBB.TITLEVEPSDTIENVK | 2.33E-06 | 2.88E-07 | 5.6210 | 0.4739 |
| MOG.VVHLYR | 2.33E-06 | 2.91E-07 | 5.6182 | 0.4005 |
| NELL2.FTGSSWIK | 2.38E-06 | 3.30E-07 | 5.5878 | 0.6212 |
| VGF.NSEPQDEGELFQGVDPR | 2.38E-06 | 3.32E-07 | 5.5857 | 0.7869 |
| SE6L1.ETGTPIWTSR | 2.42E-06 | 3.56E-07 | 5.5687 | 0.5374 |
| NCAN.APVLELEK | 3.07E-06 | 4.81E-07 | 5.4952 | 0.5915 |
| NRCAM.VFNTPEGVPSAPSSLK | 3.07E-06 | 4.97E-07 | 5.4870 | 0.5044 |
| NICA.ALADVATVLGR | 3.40E-06 | 5.75E-07 | 5.4513 | 0.5920 |
| CYTC.ALDFAVGEYNK | 3.83E-06 | 6.77E-07 | 5.4113 | 0.4275 |
| CA2D1.FVVTDGGITR | 4.13E-06 | 7.72E-07 | 5.3786 | 0.5791 |
| CNDP1.ALEQDLPVNIK | 4.13E-06 | 7.89E-07 | 5.3734 | 0.6474 |
| I18BP.LWEGSTSR | 4.65E-06 | 9.23E-07 | 5.3343 | 0.4389 |
| CNTN1.TTKPYPADIVVQFK | 5.02E-06 | 1.03E-06 | 5.3063 | 0.4162 |
| SODC.GDGPVQGIINFEQK | 6.43E-06 | 1.37E-06 | 5.2357 | 0.4055 |
| AATM.FVTVQTISGTGALR | 6.70E-06 | 1.52E-06 | 5.2100 | 0.4938 |
| CD59.AGLQVYNK | 6.70E-06 | 1.53E-06 | 5.2089 | 0.3882 |
| PCSK1.GEAAGAVQELAR | 6.84E-06 | 1.61E-06 | 5.1955 | 0.6270 |
| VASN.YLQGSSVQLR | 7.74E-06 | 1.88E-06 | 5.1566 | 0.4340 |
| PTGDS.AQGFTEDTIVFLPQTDK | 8.78E-06 | 2.21E-06 | 5.1152 | 0.2796 |
| PTPRN.AEAPALFSR | 8.78E-06 | 2.26E-06 | 5.1101 | 0.5057 |
| NBL1.LALFPDK | 8.85E-06 | 2.38E-06 | 5.0970 | 0.4037 |
| BACE1.SIVDSGTTNLR | 8.85E-06 | 2.41E-06 | 5.0935 | 0.7791 |
| KLK6.YTNWIQK | 8.85E-06 | 2.47E-06 | 5.0873 | 0.3530 |
| CSTN1.GNLAGLTLR | 9.09E-06 | 2.61E-06 | 5.0740 | 0.4493 |
| NPTXR.ELDVLQGR | 1.01E-05 | 2.96E-06 | 5.0414 | 0.4885 |
| CUTA.TQSSLVPALTDFVR | 1.03E-05 | 3.17E-06 | 5.0240 | 0.4337 |
| L1CAM.WRPVDLAQVK | 1.03E-05 | 3.18E-06 | 5.0230 | 0.5001 |
| TNR21.ASNLIGTYR | 1.07E-05 | 3.37E-06 | 5.0082 | 0.4685 |
| LPHN1.LVVSQLNPYTLR | 1.13E-05 | 3.66E-06 | 4.9873 | 0.4756 |
| IGSF8.VVAGEVQVQR | 1.29E-05 | 4.28E-06 | 4.9474 | 0.3750 |
| SCG2.IILEALR | 1.31E-05 | 4.43E-06 | 4.9385 | 0.5352 |
| APLP2.HYQHVLAVDPEK | 1.36E-05 | 4.70E-06 | 4.9234 | 0.4186 |
| NEGR1.SSIIFAGGDK | 2.21E-05 | 7.81E-06 | 4.7920 | 0.4181 |
| CADM3.EGSVPPLK | 2.39E-05 | 8.61E-06 | 4.7667 | 0.4117 |
| APOE.LGPLVEQGR | 2.88E-05 | 1.06E-05 | 4.7122 | 0.8856 |
| A4.THPHFVIPYR | 2.89E-05 | 1.08E-05 | 4.7067 | 0.4807 |
| ENOG.GNPTVEVDLYTAK | 3.90E-05 | 1.49E-05 | 4.6220 | 0.3767 |
| LAMB2.AQGIAQGAIR | 4.01E-05 | 1.56E-05 | 4.6097 | 0.4460 |
| LRC4B.HLEILQLSK | 5.26E-05 | 2.09E-05 | 4.5324 | 0.4445 |
| CNTP2.HELQHPIIAR | 7.22E-05 | 2.92E-05 | 4.4426 | 0.4313 |
| NRX2A.LSALTLSTVK | 9.13E-05 | 3.76E-05 | 4.3741 | 0.4532 |
| PEDF.TVQAVLTVPK | 9.58E-05 | 4.01E-05 | 4.3561 | 0.2516 |
| SCG3.FQDDPDGLHQLDGTPLTAEDIVHK | 9.87E-05 | 4.21E-05 | 4.3430 | 0.3988 |
| EXTL2.VIVVWNNIGEK | 0.0001 | 4.34E-05 | 4.3348 | 0.4329 |
| NEUS.ALGITEIFIK | 0.0001 | 5.69E-05 | 4.2604 | 0.4052 |
| NPTX1.FQLTFPLR | 0.0002 | 8.15E-05 | 4.1606 | 0.4421 |
| B2MG.VNHVTLSQPK | 0.0002 | 0.0001 | 4.0722 | 0.2925 |
| PVRL1.ITQVTWQK | 0.0003 | 0.0001 | 4.0534 | 0.3505 |
| NRX3A.SDLSFQFK | 0.0003 | 0.0001 | 4.0449 | 0.4619 |
| CCKN.AHLGALLAR | 0.0003 | 0.0001 | 4.0400 | 0.4959 |
| CNTN2.TTGPGGDGIPAEVHIVR | 0.0003 | 0.0002 | 3.9543 | 0.3487 |
| FBLN1.TGYYFDGISR | 0.0005 | 0.0003 | 3.8397 | 0.2494 |
| PPN.VHQSPDGTLLIYNLR | 0.0005 | 0.0003 | 3.8392 | 0.3959 |
| SORC1.TIAVYEEFR | 0.0005 | 0.0003 | 3.8227 | 0.4280 |
| SCG1.NYLNYGEEGAPGK | 0.0005 | 0.0003 | 3.8104 | 0.3598 |
| PRDX1.DISLSDYK | 0.0007 | 0.0004 | 3.7361 | 0.4120 |
| B3GN1.TALASGGVLDASGDYR | 0.0007 | 0.0004 | 3.7165 | 0.2480 |
| SPRL1.VLTHSELAPLR | 0.0007 | 0.0004 | 3.7096 | 0.2339 |
| NRX1A.SDLYIGGVAK | 0.0008 | 0.0005 | 3.6623 | 0.3763 |
| BTD.LSSGLVTAALYGR | 0.0010 | 0.0005 | 3.6159 | 0.2708 |
| GOLM1.QQLQALSEPQPR | 0.0013 | 0.0007 | 3.5241 | 0.3364 |
| COCH.GVISNSGGPVR | 0.0015 | 0.0008 | 3.4764 | 0.3863 |
| CD14.SWLAELQQWLKPGLK | 0.0023 | 0.0013 | 3.3301 | 0.2616 |
| DIAC.ATYIQNYR | 0.0023 | 0.0014 | 3.3236 | 0.2240 |
| CSTN3.ESLLLDTTSLQQR | 0.0026 | 0.0016 | 3.2809 | 0.2677 |
| MIME.ETVIIPNEK | 0.0026 | 0.0016 | 3.2770 | 0.3531 |
| GRIA4.LQNILEQIVSVGK | 0.0040 | 0.0024 | 3.1407 | 0.2536 |
| IBP2.LIQGAPTIR | 0.0060 | 0.0037 | 2.9956 | 0.2270 |
| MUC18.GATLALTQVTPQDER | 0.0063 | 0.0039 | 2.9755 | 0.2223 |
| PDYN.LSGSFLK | 0.0070 | 0.0044 | 2.9367 | 0.2532 |
| TIMP1.GFQALGDAADIR | 0.0090 | 0.0057 | 2.8440 | 0.2256 |
| KLK10.ALQLPYR | 0.0144 | 0.0092 | 2.6709 | 0.4076 |
| SLIK1.SLPVDVFAGVSLSK | 0.0160 | 0.0103 | 2.6282 | 1.2766 |
| CH3L1.ILGQQVPYATK | 0.0181 | 0.0118 | 2.5782 | 0.3283 |
| CATL1.VFQEPLFYEAPR | 0.0257 | 0.0170 | 2.4397 | 0.1791 |
| PIMT.VQLVVGDGR | 0.0363 | 0.0243 | 2.2974 | 0.2446 |
| BASP1.ETPAATEAPSSTPK | 0.0412 | 0.0279 | 2.2415 | 1.0052 |
| TRFM.ADTDGGLIFR | 0.0454 | 0.0310 | 2.1969 | 0.2217 |
| NPTX2.LESLEHQLR | 0.0457 | 0.0316 | 2.1895 | 0.2833 |
| CATD.LVDQNIFSFYLSR | 0.0587 | 0.0411 | -2.0768 | -0.1138 |
| FMOD.YLPFVPSR | 0.0587 | 0.0415 | 2.0734 | 0.3008 |
| GFAP.ALAAELNQLR | 0.0598 | 0.0426 | 2.0611 | 0.2328 |
| CLUS.VTTVASHTSDSDVPSGVTEVVVK | 0.0605 | 0.0436 | 2.0516 | 0.1726 |
| PGRP2.AGLLRPDYALLGHR | 0.0993 | 0.0723 | 1.8222 | 0.2909 |
| PRDX3.HLSVNDLPVGR | 0.1182 | 0.0869 | 1.7340 | 0.9805 |
| CO2.SSGQWQTPGATR | 0.1298 | 0.0964 | 1.6830 | 0.1966 |
| ITIH5.SYLEITPSR | 0.1482 | 0.1112 | 1.6114 | 0.1349 |
| FBLN3.LTIIVGPFSF | 0.1801 | 0.1364 | 1.5050 | 0.1326 |
| CO6.SEYGAALAWEK | 0.2015 | 0.1547 | 1.4373 | 0.2248 |
| NGF.SAPAAAIAAR | 0.2015 | 0.1556 | 1.4340 | 0.1765 |
| KAIN.FYYLIASETPGK | 0.2037 | 0.1602 | 1.4180 | 0.2415 |
| CO5.VFQFLEK | 0.2037 | 0.1602 | 1.4179 | 0.2596 |
| HEMO.NFPSPVDAAFR | 0.2132 | 0.1693 | 1.3873 | 0.2636 |
| CO3.TELRPGETLNVNFLLR | 0.2502 | 0.2006 | 1.2910 | 0.1527 |
| CATA.LFAYPDTHR | 0.2590 | 0.2101 | 1.2638 | 0.4714 |
| CERU.NNEGTYYSPNYNPQSR | 0.2590 | 0.2114 | 1.2601 | 0.1870 |
| ENPP2.SYPEILTLK | 0.2646 | 0.2179 | -1.2421 | -0.0635 |
| SIAE.ELSNTAAYQSVR | 0.2717 | 0.2257 | -1.2212 | -0.1350 |
| AFAM.FLVNLVK | 0.3214 | 0.2694 | -1.1123 | -0.1818 |
| CO4A.GSFEFPVGDAVSK | 0.3293 | 0.2785 | 1.0914 | 0.1882 |
| AACT.ADLSGITGAR | 0.3373 | 0.2877 | 1.0705 | 0.1357 |
| ITIH1.EVAFDLEIPK | 0.3725 | 0.3205 | 0.9999 | 0.2246 |
| TGFB1.LLAPSDSPEWLSFDVTGVVR | 0.3803 | 0.3300 | 0.9803 | 0.3127 |
| TTHY.TSESGELHGLTTEEEFVEGIYK | 0.3919 | 0.3429 | -0.9543 | -0.0700 |
| THRB.YGFYTHVFR | 0.4345 | 0.3834 | 0.8766 | 0.1333 |
| A1AT.SVLGQLGITK | 0.4484 | 0.3989 | 0.8482 | 0.1325 |
| PLMN.EAQLPVIENK | 0.4739 | 0.4252 | 0.8017 | 0.1581 |
| CO8B.YEFILK | 0.4812 | 0.4352 | 0.7844 | 0.1567 |
